# Cascade *N*-Alkylation/Hemiacetalization for Facile Construction of the Spiroketal Skeleton of Acortatarin Alkaloids with Therapeutic Potentiality in Diabetic Nephropathy

**DOI:** 10.1007/s13659-014-0049-8

**Published:** 2014-12-16

**Authors:** Pei Cao, Zhen-Jie Li, Wen-Wu Sun, Shashwat Malhotra, Yuan-Liang Ma, Bin Wu, Virinder S. Parmar

**Affiliations:** 1 State Key Laboratory of Phytochemistry and Plant Resources in West China, Kunming Institute of Botany, Chinese Academy of Sciences, Kunming, 650201 People’s Republic of China; 2 University of Chinese Academy of Sciences, Beijing, 100049 People’s Republic of China; 3 Bioorganic Laboratory, Department of Chemistry, University of Delhi, Delhi, 110007 India; 4 Institute of Chemistry and Biochemistry, Freie Universität Berlin, Takustrasse 3, 14195 Berlin, Germany

**Keywords:** Acortatarin alkaloids, Diabetic nephropathy, *N-*alkylation/hemiacetalization, Halomethylation, Chiral lactones

## Abstract

The concise building of the spiroketal core of acortatarin-type alkaloids as potential therapeutic agents in diabetic nephropathy was established in four steps, through a tandem *N*-alkylation/hemiacetalization between pyrrole units and the corresponding halo alcohols generated by convenient halomethylation of chiral lactones from natural aldoses.

The Acortatarin alkaloids exhibit a promising perspective against diabetic nephropathy and other ROS-linked diseases. Facile construction of the dioxaspirocycle motif was realized through tandem *N-*alkylation/hemiacetalization between pyrrole units and the corresponding halo alcohols, which were derived in turn from convenient halomethylation of chiral lactones in natural carbohydrates pool.

**Electronic supplementary material:**

The online version of this article (doi:10.1007/s13659-014-0049-8) contains supplementary material, which is available to authorized users.

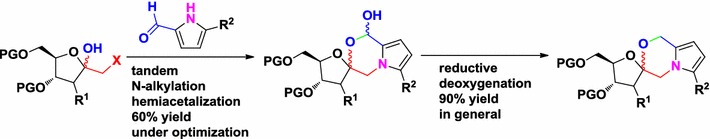

## Introduction

**Fig. 1:**
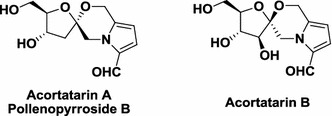
Revised structures of acortatarins A and B

Acortatarins A and B along with their structurally related analogues were independently isolated by three groups in 2010, from different natural sources used as traditional folk medicinal substances against a variety of ailments, including central nervous system disorders [1], prostatitis [2] and rheumatics [3]. These compounds with the unique pyrrole-fused morpholine spiroketal architecture, possess a small group of scaffolds containing 2,5-bis-substituted pyrrole unit. Hou and Cheng demonstrated that acortatarins A and B have significant antioxidant activity in a renal cell model for high-glucose-stimulated production of reactive oxygen species (ROS). Their results will provide a new template for further therapeutic intervention in diabetic nephropathy (DN) and other ROS-linked diseases [1]. Though they display promising and wide ranging biological properties, yet the strenuous extraction and purification procedures with very less yields of these special secondary metabolites from natural sources restrict their further biological investigation. Thus, considerable synthetic efforts have evoked to develop novel strategies for acortatarin analogues Fig 1.

Sudhakar [4] disclosed the first total syntheses of acortatarins A and B followed by structural revisions of absolute configurations on both. Subsequently, Brimble [5] and Aponick [6] also finished the synthesis of acortatarin A. The wide-spread utility of these synthetic methodologies for acortatarins is limited because of tedious multi-steps (13 or more), low overall yields and disappointing or even undesired diastereoselectivity for spiroketalization. Compared superior to these methods, Tan et al. [7] developed an elegant, stereocontrolled syntheses of acortatarins A and B, however starting from expensive d-thymidine, using stoichiometric amounts of toxic mercury salt in the key step for acortatarin A. Recently, Kuwahara and coworkers [8] reported their expeditious route for acortatarin A derived from elaborately protected chiral olefin, nevertheless, associated with higher cost and harsher deprotection/cyclization steps. This particular architecture which appears simple comprises a significant and attractive synthetic challenge indeed. Herein, we report our efforts towards facile building of the spiroketal core by extending the application of halo alcohols, which were generated by convenient halomethylation of chiral lactones from natural aldoses. An interesting tandem *N-*alkylation/hemiacetalization with pyrrole units was established to provide an alternative route for the target compounds.

## Results and Discussion

**Scheme 1:**
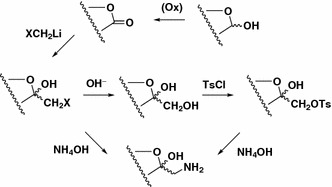
Transformations among halomethyl, hydroxymethyl and aminomethyl groups in ketose

**Scheme 2:**
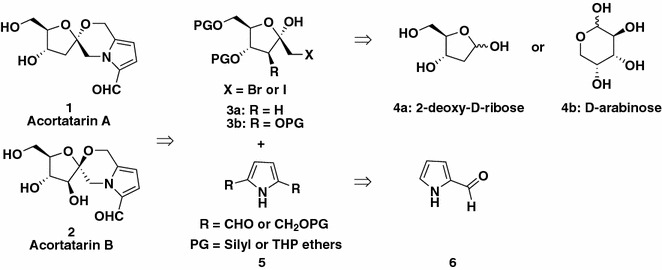
Retrosynthetic route through key halomethylation of lactones from natural carbohydrates

The synthetic entries mentioned above are mainly based on S_N_2 reaction between the cyclic or acyclic ketose-type derivatives (epoxides [4], bromides [6, 8] or iodides [7]) and the pyrrole fragments, only with one example employing Maillard-type condensation [5]. Based upon the proposed biogenesis of acortatarins [2], we envisaged the chemoselective Amadori rearrangement to get the furanoid amino sugars, but our attempts did not give acceptable preparative yields (see supporting information). The work of Morin [9] and Wadouachi [10] made us recognize that the iodomethyl group acts as an interchanging hydroxymethyl or aminomethyl synthetic equivalent (Scheme 1). At the meanwhile, we noticed that the regioselective protection of fructofuranosyl acceptors directly from d-fructose needs time-consuming long process [11]. Araújo [12] described an efficient approach for the furanoside originating from arabinose, taking advantage of the fixed stereochemistry of the arabinolactone, which was easily prepared by the oxidation of the anomeric position with bromine in water. The one-carbon elongation of aldoses to ketoses in these cases used lithium reagent for the key homologation step. Enlightened by Araújo’s successful access to furanoside [12], an alternative retrospective route was outlined to obtain the key intermediates 3**a** and 3**b** from the readily available 2-deoxy-d-ribose (**4a**) [13] and d-arabinose (**4b**) [14], respectively. Similar to the protocol of Tan [7], the subsequently direct S_N_2 coupling with pyrrole partner **5** [10] could bridge the two subunits (Scheme 2). We reasoned that this plan might offer additional strategic advantages in that all the requisite stereochemistry of the hydroxyl groups for **1** and **2** would be attained or kept along with the initial natural aldoses from the beginning to the end. The versatility of assembling other unnatural acortatarin-type alkaloids was to succeed in conjunction with the implementation of one-step haloalkylation on natural chirons.

**Scheme 3:**
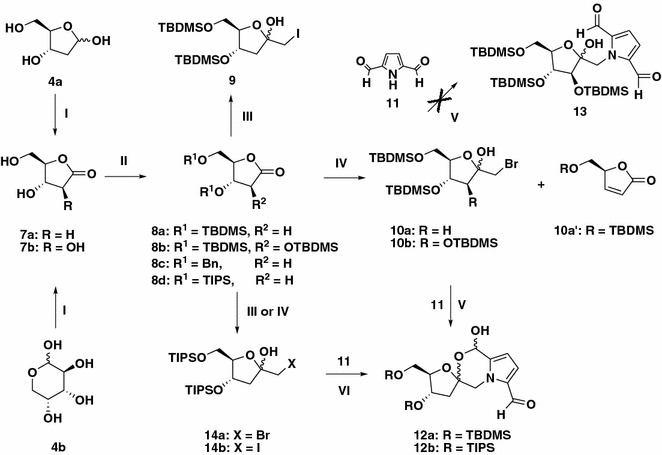
Unsatisfactory experimental trials with modification. (I) K_2_CO_3_, H_2_O, Br_2_, 0 °C to rt. (II) a TBDMSCl, imidazole, DMAP, DMF, 0 °C to rt, 75 % over two steps for **4a** to **8a** through **7a**; **4b** to **8b** through **7b**; b 2,4,6-tris(benzyloxy)-1,3,5-triazine, TfOH, dioxane, 0 °C to rt, 65 % over two steps for **4a** to **8c** through **7a**; c TIPSOTf, lutidine, DMAP, DMF, 0 °C to rt, 70 % over two steps for **4a** to **8d** through **7a**. (III) CH_2_I_2_, *n*-BuLi, toluene, −82 °C, 70 % for **8d** to **14b**. (IV) CH_2_Br_2_, *n*-BuLi, THF, −78 °C, 31 % for **8a** to **10a**; 36 % for **8a** to **10a**′; 71 % for **8b** to **10b**; 36 % for **8d** to **14a**. (V) NaH, THF, 0 °C to rt, 40 % for **10a** to **12a**. (VI) Cs_2_CO_3_, MeCN, rt, 60 % for **14b** to **12b**

With the goal of devising a concise synthesis of this uncommon core, the exploration was initiated with the preparation of lactones **7a** and **7b** by the oxidation of **4a** and **4b** buffered in an aqueous K_2_CO_3_ solution (Scheme 3) [14], then subjected to one-pot protection without further purification to get TBDMS ethers **8a** and **8b** in approximately 75 % overall yield for both (Scheme 3) [15]. Previously employed iodomethylation [9] for the conversion of **8a** to **9** was proved to be unfavorable in this case, because it was very difficult to separate the iodide **9** from starting material recovered by chromatography along with liable decomposition in chloroform (or CDCl_3_). The focus shifted to the more stable bromomethylating compounds prepared by BrCH_2_Li (in situ generation from CH_2_Br_2_ and *n*-BuLi in the presence of LiBr) [16]. Unfortunately, an uncharacterized side product was observed after purification with slight decomposition by treatment of **8a** with these combinational reagents. We were gratified to really furnish **10a** as inseparable anomeric isomers (about 1:1 ratio) after venturously removing the additive LiBr. The yield (31 %) was modest, and the major byproduct, dihydrofuranone **10a′** [17] was delivered in 36 % yield. All attempts to enhance the ratio by changing the solvent (THF, toluene, DME), the amount of BrCH_2_Li (1, 1.5, 2 equivalents), reaction temperature (−65 or −78 °C), running-time (0.5, 1, 2 h), or work-up in acidic medium (quenching by saturated ammonium chloride solution or equivalent acetic acid) failed (Scheme 3).

**Scheme 4:**
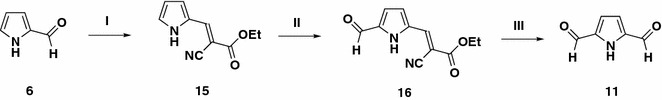
Synthesis of pyrrole-2,5-dicarbaldehyde. (I) CNCH_2_CO_2_Et, piperidine, EtOH, reflux. (II) oxalyl chloride − DMF, MW, 0–100 °C. (III) 3 M NaOH, reflux, 45 % over three steps for 6 to 11

Pleasingly, **10b** could be obtained in 71 % yield from **8b**. With bromides **10a** and **10b** in hand to advance the synthesis, we decided to test the outcome of S_N_2 coupling with bisformyl pyrrole **11**. The three-step sequence [18] with assistance of microwave for the Vilsmeier–Haack formylation [19] proceeded well to afford **11** in 45 % yield (Scheme 4). To our delight, **12a** (nearly 1/1 diastereoisomers) was generated directly in about 40 % yield using NaH [10] with unanticipated formation of semiacetal ring which was confirmed by 1D and 2D NMR. From the more bulky **10b**, reaction did not afford **13** at room temperature. Moreover, decomposition happened under reflux condition (Scheme 3). The replacement of NaH/THF system by Cs_2_CO_3_/MeCN [6], NaOH/toluene/H_2_O [7] did not lead to any improvements because of the inhibition of lactol formation.

The detailed functional compatibility resulted in the improvement for preparation of benzyl ethers (Scheme 3). Some problems were associated with the conventional *O*-benzylation methods in our hands, such as low yield for normal or mildly basic Williamson conditions (NaH/BnBr [4] or Ag_2_O/BnBr [9] ), tedious purification steps for benzyl 2,2,2-trichloroacetimidate (BTCAI) methodology [12, 20]. Better result was achieved finally to get benzyl ether **8c** through 2,4,6-tris(benzyloxy)-1,3,5-triazine (TriBOT) [21] catalyzed by TfOH in one-pot (65 % total yield without further optimization) (Scheme 3). All the glitches with other protecting protocols including acetalization, ketalization, disilyl (TIPDSCl), THP, MOM, allyl ethers were tried, among which, excellent regioselectivity was realized to afford (3*S,*5*R*)-5-*O*-allyl-3-hydroxy-d-ribonolactone with moderate yield, by 2,4,6-tris(allyloxy)-1,3,5-triazine (TriAOT) (see supporting information). Due to the epimerization of debenzylation accomplished by TiCl_4_ [4, 6], we still wanted to make progress towards the appropriate silyl groups whose deprotection was much milder. Sauve [15] showed that the β-elimination in electrophilic fluorination of 2-deoxyribonolactone can be successfully mitigated by steric triisopropylsilyl (TIPS) bulk at the susceptible β-position. So TIPS-protected lactone **8d** [15] was introduced and used for bromomethylation [16]. This precursor was unproductive to completely inhibit β-elimination with the major unsaturated dihydrofuranone easily monitored by basic KMnO_4_ solution. The isolated yield of **14a** (nearly 1/1 diastereoisomers) was frustrating again (about 36 %) (Scheme 3). The iodomethylation of **8d** with CH_2_I_2_ and *n*-BuLi (even 1.2/1 equivalents) was very complicated in THF. Changing the solvent to toluene [9] gave very slow conversion to **14b** even after 3 h, as shown by the crude NMR of the reaction mixture (see supporting information). However, we noticed that the β-elimination becomes negligible at low temperature (−82 °C). We increased the ratio of CH_2_I_2_ and *n*-BuLi gradually. Finally, about 70 % isolated yield for **14b** (nearly 1/1 diastereoisomers) was realized with a slight excess of CH_2_I_2_ and *n*-BuLi (3/2.8 equivalents) after running the reaction for 3 h under −82 °C (Scheme 3).

**Scheme 5:**
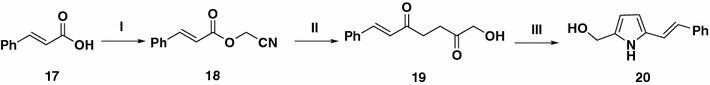
Synthesis of bis-substituted pyrroles. (I) Et_3_N, ClCH_2_CN, dichloroethane, 0 °C to rt, 75 %. (II) EtMgBr, Ti(O-*i*-Pr)_4_, diethyl ether, 0 °C to rt, 70 %. (III) NH_4_OAc, NaOAc, EtOH, MW, 40 °C, quantitative conversion

**Scheme 6:**
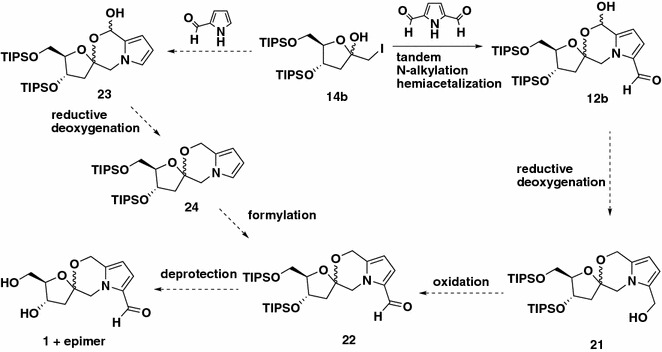
On-going synthetic perspectives of acortatarin A

Till this point, the unexpected tandem *N*-alkylation and concomitant semiacetalization for the lactol ring formation was the main problem left. 1,5-Disubstituted 5-hydroxy-1,4-diketone **19** [22] was introduced to allow the construction of bis-substituted pyrrole **20** with aldehyde group masked as olefin (Scheme 5). Both **18** and **19** were obtained as reported with moderate yields in two steps [22]. Paal–Knorr condensation of diketones **19** with NH_4_OAc were carried out under a variety of buffered or acidic conditions (HOAc-NaOAc, *p*-TsOH, CSA, I_2_). Only polymeric products or decomposition resulted, probably because the acid-catalyzed decomposition of the pyrrolic products is competitive with their formation. Ultimately, after screening dioxane, DME, THF, MeCN, EtOH, MeOH and H_2_O as solvents, one mild neutral buffering system worked only in EtOH (NH_4_OAc/NaOAc = 3.5/1.2 equivalents) to afford pyrrole unit **20** quantitatively. The direct coupling between iodide **14b** and **20** proved ineffective. Moreover, the unpredictability of oxidative cleavage of olefins to aldehydes and the additive protection/deprotection for hydroxyl group in **20** put us in a dilemma. At this juncture, the reductive deoxygenation of lactol to benzopyran achieved by exposure to NaBH_4_ [23] or triethylsilane (Et_3_SiH) [24] in the presence of TFA attracted our attention. Thus we could continue and complete the expected total synthesis of acortatarin A as illustrated in Scheme 6.

*N*-alkylation of pyrrole **11** with iodide **14b** could deliver hemiacetal **12b** (Cs_2_CO_3_/MeCN [6], nearly 1/1 diastereoisomers, 60 % yield without further optimization). The reduction of lactol **12b** to create morpholine motif **21** would not be problematic though the free aldehyde could not be kept. The oxidation to **22** and deprotection of silyl ether for acortatarin A and its epimer could be finished according to the known literature methods. To minimize the synthesis steps and maximize the overall efficiency of our strategy, one alternative route was proposed to introduce the second aldehyde group by formylation of **24** at a later stage as depicted.

## Conclusion

We hypothesized that the thermodynamic stability of the final products as the driving force in the cascade *N*-alkylation and concomitant semiacetalization. The spontaneous spiro-hemiacetalization was induced by the deprotonation of hydroxyl group adhering to the anomeric carbon at the ring junction position, followed by sequential attack of the resulting oxygen anion aiming at neighboring aldehyde group. This crucial semiketal carbon serves as one incorporating “middle hinge” necessary for the simultaneous shutting of the crab-claw-shaped morpholine ring (Scheme 6). It may supply one alternative route for the troublesome coupling of pyrrole units bearing sterically hindered THP or TBDMS protected hydroxyl groups [4].

For the importance of deoxyribonucleotide chemistry, 2-deoxy-d-ribose derivatives were found to be more advantageous. But the stepwise improved halomethylating condition is adaptable within wider scope. The present experiences were envisioned to allow the assembly of the target molecules efficiently, including the construction of all the subunits in the near future. The key elements in this synthesis sequence include the optimized halomethylation for lactones and tandem nucleophilic substitution. The other important features lie in prompting the utility of natural sugars as the chiral pool inspired by the biogenetic hypothesis. Obviously, when combined with the unnatural formylated pyrrole analogues with *para*-substitutions (C-2/5) (Scheme 5), such a synthetic strategy is also readily performed for other novel acortatarins. The pursuit towards these goals is currently underway, and the results will be reported in due course.

## Experiment Section

Melting points were measured on an SGW X-4 melting instrument and uncorrected. MS were recorded on a Waters AutoSpec Premier P776 or API STAR Pulsar instrument. NMR spectra were recorded on a Bruker AM-400 or DRX-500 spectrometer and calibrated using residual solvent peaks as internal reference, for example CDCl_3_ solutions. TLC analyses were performed on commercial glass plates bearing 0.25 mm layer of Merck Silica gel 60F_254_. Silica gel (200–300 mesh, Qingdao Marine Chemical Co. Ltd., People’s Republic of China) was used for general column chromatography. Spots on TLC plates were detected under UV radiation and by heating after spraying with 10 % H_2_SO_4_ in EtOH or dilute solutions of KMnO_4_. Microwave irradiation reactions were carried out in a CEM Discover SP system. Tetrahydrofuran (THF), diethyl ether and toluene were distilled over sodium/benzophenone. Other reagents were obtained from commercial sources and used without further purification unless otherwise stated.

### 2-Deoxy-3,5-di-*O*-(*tert*-butyldimethylsilyl)-d-ribonolactone (**8a**)

To a solution of 2-deoxy-d-ribose **4a** (1.0 g, 7.5 mmol) in 6 mL water, K_2_CO_3_ (1.2 g, 8.9 mmol) was added in portions. After the clear aqueous solution was cooled to 0 °C, Br_2_ (1.3 g, 8.2 mmol) was added dropwise. The resulting orange solution was warmed to rt and stirred for 10 h. The reaction mixture was quenched and neutralized by the addition of 88 % formic acid, the solvent from the aqueous fraction was evaporated to give a pale brown syrup **7a**, which was heated at 50 °C under reduced pressure for 1 h. EtOH (20 mL) was added, and the solvent was repeatedly evaporated after each addition of EtOH. Without further purification, the crude product **7a** was dissolved in 20 mL of anhydrous DMF, to which imidazole (2.5 g, 37.3 mmol) and *tert*-butyldimethylsilyl chloride (4.5 g, 29.8 mmol) were added. The resulting solution was stirred at rt for 24 h and quenched by addition of water. The water layer was extracted with ethyl acetate (30 mL × 3), and the organic layers were combined, washed with brine, and dried over anhydrous Na_2_SO_4_. The crude product was concentrated in vacuo. Flash chromatography afforded **8a** as a white solid: mp 72–73 °C. Yield: 2.03 g (75 % over two steps) [14, 15].

^1^H NMR (400 MHz, CDCl_3_) *δ* 4.49 (dt, *J* = 6.8, 2.0 Hz, 1H), 4.33 (dd, *J* = 4.7, 2.3 Hz, 1H), 3.80 (dd, *J* = 11.5, 3.2 Hz, 1H), 3.75 (dd, *J* = 11.5, 2.4 Hz, 1H), 2.81 (dd, *J* = 17.6, 6.8 Hz, 1H), 2.38 (dd, *J* = 17.6, 2.4 Hz, 1H), 0.88 (s, 18H), 0.08 (s, 6H), 0.07 (s, 3H), 0.05 (s, 3H). ^13^C NMR (100 MHz, CDCl_3_) *δ* 175.98, 88.22, 69.71, 62.53, 39.13, 25.87, 25.73, 18.31, 17.99, −4.69, −4.76, −5.45, −5.57.

**ESI-MS***m/z* (rel int): (pos) 383 ([M + Na]^+^, 100).

### 2,3,5-Tri-*O*-(*tert*-butyldimethylsilyl)-d-arabino-1,4-lactone (**8b**)

The synthesis of lactone repeated the known procedure [14]. The *O*-silylation followed the literature method [15]. **8b** was afforded as colorless oil. Yield: 368 mg (75 % over two steps).

^1^H NMR (400 MHz, CDCl_3_) *δ* 4.34 (dd, *J* = 15.6, 7.1 Hz, 1H), 4.30 (d, *J* = 7.1 Hz, 1H), 4.06 (dt, *J* = 6.3, 3.1 Hz, 1H), 3.90 (dd, *J* = 12.0, 2.9 Hz, 1H), 3.76 (dd, *J* = 12.0, 3.3 Hz, 1H), 0.92–0.88 (overlapping s, 27H), 0.16–0.08 (overlapping s, 18H). ^13^C NMR (100 MHz, CDCl_3_) *δ* 173.38, 82.21, 76.35, 74.03, 60.46, 25.92, 25.87, 25.81, 18.40, 18.33, 17.94, −4.11, −4.25, −4.61, −4.72, −5.21, −5.30.

**ESI-MS***m/z* (rel int): (pos) 514 ([M + Na]^+^, 100).

### 2,4,6-Tris(benzyloxy)-1,3,5-triazine (TriBOT)

The synthesis of this reagent followed the known procedure, but using 1.2 equivalents NaH instead of NaOH and 1.1 equivalents BnOH in THF, respectively. White solid: mp 106–108 °C. Yield: 638 mg (80 %) [21].

^1^H NMR (500 MHz, CDCl_3_) *δ* 7.45–7.32 (m, 15H), 5.45 (s, 6H). ^13^C NMR (100 MHz, CDCl_3_) *δ* 173.12, 135.41, 128.69, 128.55, 128.42, 70.04.

### 3,5-Di-*O*-benzyl-d-ribonolactone (**8c**)

The synthesis of lactone repeated the known procedure [14]. The *O*-benzylation followed the literature method [21]. **8c** was afforded as colorless oil. Yield: 101 mg (65 % over two steps) [20].

^1^H NMR (400 MHz, CDCl_3_) *δ* 7.39–7.26 (m, 10H), 4.64 (dd, *J* = 4.8, 2.9 Hz, 1H), 4.52 (m, 4H), 4.28 (dt, *J* = 6.8, 1.9 Hz, 1H), 3.65 (qd, *J* = 10.7, 3.1 Hz, 2H), 2.87 (dd, *J* = 18.1, 6.9 Hz, 1H), 2.58 (dd, *J* = 18.1, 2.2 Hz, 1H). ^13^C NMR (100 MHz, CDCl_3_) *δ* 175.73, 137.43, 137.13, 128.72, 128.66, 128.22, 128.08, 127.88, 127.74, 84.14, 76.19, 73.82, 71.31, 69.70, 35.86.

**EI-MS***m/z* (rel int): 312 (M^+^), 221 (M^+^−91), 107, 91 (100).

### 2-Deoxy-3,5-di-*O*-(triisopropylsilyl)-d-ribonolactone (**8d**)

The synthesis of lactone repeated the known procedure [14]. The *O*-silylation followed the literature method, however, with slight modification by combinational TIPSOTf, lutidine (instead of TBDMSCl, imidazole) and DMAP in DMF [15]. **8d** was afforded as colorless oil. Yield: 1.0 g (75 % over two steps).

^1^H NMR (400 MHz, CDCl_3_) *δ* 4.65 (d, *J* = 6.4 Hz, 1H), 4.40 (s, 1H), 3.92 (dd, *J* = 11.3, 2.9 Hz, 1H), 3.86 (dd, *J* = 11.6, 2.0 Hz, 1H), 2.88 (dd, *J* = 17.6, 6.5 Hz, 1H), 2.42 (d, *J* = 17.6 Hz, 1H), 1.04 (s, 42H). ^13^C NMR (100 MHz, CDCl_3_) *δ* 175.97, 88.71, 69.93, 63.19, 39.54, 17.82, 11.89, 11.91.

**ESI-MS***m/z* (rel int): (pos) 467 ([M + Na]^+^, 100).

### 1-Bromo-1,3-dideoxy-4,6-di-*O*-(*tert*-butyldimethylsilyl)-d-fructofuranose (**10a**)

Under nitrogen protection, to a mixed solution of lactone **8a** (360 mg, 1.0 mmol) and dibromomethane (348 mg, 2.0 mmol) in dry THF (10 mL) cooled to −78 °C was added dropwise a solution of *n*-butyllithium in hexane (2.4 M solution, 0.6 mL, 1.5 mmol). The solution was maintained at −78 °C for 1 h then quenched with a saturated solution of ammonium chloride in water (about 4 mL). The organic layer was decanted, and the aqueous layer was extracted with diethyl ether (10 mL × 2), the combined organic layers were dried over anhydrous Na_2_SO_4_ and the solvents evaporated under reduced pressure. Flash chromatography gave bromo alcohol **10a** as pale yellow oil [9, 16]. Yield: 141 mg (31 %).

Isomer A (54 %) ^1^H NMR (400 MHz, CD_3_COCD_3_) *δ* 4.90 (br.s, 1H, OH), 4.41 (dt, *J* = 6.7, 4.4 Hz, 1H), 4.05 (q, *J* = 4.4 Hz, 1H), 3.68–3.66 (m, 2H), 3.49 (d, *J* = 2.1 Hz, 2H), 2.49 (dd, *J* = 13.3, 6.9 Hz, 1H), 1.99 (dd, *J* = 13.3, 4.4 Hz, 1H), 0.91 (s, 18H), 0.12–0.08 (overlapping s, 12H). ^13^C NMR (100 MHz, CD_3_COCD_3_) *δ* 104.94, 87.34, 73.57, 63.42, 44.84, 38.51, 26.30, 26.12, 18.89, 18.45, −4.58, −4.67, −5.22.

Isomer B (46 %) ^1^H NMR (400 MHz, CD_3_COCD_3_) *δ* 5.10 (br.s, 1H, OH), 4.50 (dt, *J* = 5.8, 4.8 Hz, 1H), 3.87 (dd, *J* = 9.2, 4.8 Hz, 1H), 3.68–3.66 (m, 2H), 3.65 (d, *J* = 1.7 Hz, 1H), 3.56 (d, *J* = 10.3 Hz, 1H), 2.25 (qd, *J* = 13.3, 5.5 Hz, 2H), 0.91 (s, 18H), 0.12–0.08 (overlapping s, 12H). ^13^C NMR (100 MHz, CD_3_COCD_3_) *δ* 105.58, 88.67, 73.79, 64.55, 44.44, 39.66, 26.30, 26.12, 18.89, 18.45, −4.58, −4.67, −5.22.

**ESI-MS***m/z* (rel int): (pos) 477 ([M + Na]^+^).

### (*5S*)-5-(*tert*-Butyldimethylsilanyloxymethyl)-5H-furan-2-one (**10a′**)

The modified procedure for bromomethylation was as same as that of **10a** [9, 16], the side-product dihydrofuranone **10a′** was afforded as pale yellow oil. Yield: 82 mg (36 %) [17].

^1^H NMR (400 MHz, CDCl_3_) *δ* 7.49 (dd, *J* = 5.7, 1.0 Hz, 1H), 6.16 (dd, *J* = 5.7, 1.6 Hz, 1H), 5.05 (t, *J* = 4.7 Hz, 1H), 3.92 (dd, *J* = 10.8, 4.4 Hz, 1H), 3.80 (dd, *J* = 10.8, 5.3 Hz, 1H), 0.87 (s, 9H), 0.06 (s, 6H). ^13^C NMR (100 MHz, CDCl_3_) *δ* 173.15, 154.45, 122.64, 83.47, 63.03, 25.99, 22.83, 14.27, −5.38.

### 1-Bromo-1-deoxy-3,4,6-tri-*O*-(*tert*-butyldimethylsilyl)-d-fructofuranose (**10b**)

The modified procedure for bromomethylation was as same as that of **10a** [9, 16], **10b** was afforded as colorless oil. Yield: 416 mg (71 %).

Isomer A (57 %) ^1^H NMR (500 MHz, CD_3_COCD_3_) *δ* 4.49 (d, *J* = 1.9 Hz, 1H), 4.27–4.26 (m, 1H), 4.22–4.21 (m, 1H), 3.83–3.79 (m, 1H), 3.71 (d, *J* = 6.0 Hz, 1H), 3.69 (d, *J* = 10.5 Hz, 1H), 3.66 (d, *J* = 6.0 Hz, 1H), 3.58 (d, *J* = 10.5 Hz, 1H), 0.98–0.82 (overlapping s, 27H), 0.24–0.09 (overlapping s, 18H). ^13^C NMR (125 MHz, CD_3_COCD_3_) *δ* 105.78, 87.11, 79.60, 79.51, 64.51, 36.41, 26.37, 26.33, 26.28, 26.12, 26.08, 18.88, 18.77, 18.54, 18.38, −3.80, −4.22, −4.30, −4.34, −4.45, −4.52, −4.68, −5.10, −5.17.

Isomer B (43 %) ^1^H NMR (500 MHz, CD_3_COCD_3_) *δ* 4.67 (d, *J* = 1.0 Hz, 1H), 4.24–4.23 (m, 1H), 4.10–4.09 (m, 1H), 4.08–4.07 (m, 1H), 3.71 (d, *J* = 6.0 Hz, 1H), 3.66 (d, *J* = 6.0 Hz, 1H), 3.54 (dd, *J* = 10.3, 1.1 Hz, 1H), 3.47 (d, *J* = 10.3 Hz, 1H), 0.98–0.82 (overlapping s, 27H), 0.24–0.09 (overlapping s, 18H). ^13^C NMR (125 MHz, CD_3_COCD_3_) *δ* 106.75, 87.92, 82.58, 79.64, 64.38, 34.88, 26.37, 26.33, 26.28, 26.12, 26.08, 18.88, 18.77, 18.54, 18.38, −3.80, −4.22, −4.30, −4.34, −4.45, −4.52, −4.68, −5.10, −5.17.

**ESI-MS***m/z* (rel int): (pos) 609 ([M + Na]^+^, 100).

## 1H-Pyrrole-2,5-dicarbaldehyde (**11**)

A solution of pyrrole-2-aldehyde **6** (0.48 g, 5 mmol), ethyl cyanoacetate (0.68 g, 6 mmol) and piperidine (0.05 mL) in ethanol (5 mL) was stirred at rt for 1 h then heated to reflux for 2 h under the protection of nitrogen. The reactants were cooled to rt, the orange solids precipitated were filtrated and washed by petroleum ether, after removal of the volatiles in vacuo, **15** was obtained in quantitative yield. 3 mL DMF was cooled below 5 °C in a round-bottom flask equipped with a stir bar, under protection of nitrogen, oxalyl chloride (0.76 g, 6 mmol) was added under vigorous stirring, the cooling bath was removed, 30 min later, the mixture was cooled to 0 °C again. A solution of compound **15** in 2 mL of DMF was added and the resulting mixture was allowed to stir in an ice bath for 45 min and at rt for 2 h. The flask was then quickly fitted with a short condenser pipe protected by calcium chloride drying tube and heated by microwaves at 100 °C for 12 min in a CEM-microwave reactor. After cooling to rt, a solution of saturated aqueous NaHCO_3_ was added carefully to adjust the pH to slightly basic, and the mixture was heated for 15–30 min under reflux. After cooling, the crude precipitate **16** was filtered off and added to a solution of 15 mL 3 M NaOH, the mixture was refluxed for 2 h under nitrogen, cooled, neutralized with 6 M hydrochloride acid in an ice bath, the precipitate resulted was filtered. The aqueous phase was extracted by ethyl acetate (20 mL × 3), the total organic level was dried over anhydrous Na_2_SO_4_, after removal of the solvents in vacuo, the solids filtrated previously and the organic phase were combined and subjected to flash column chromatography on silica gel to afford **11** as pale yellow solid: mp 120–122 °C. Yield: 0.32 g (52 % over three steps) [18, 19].

^1^H NMR (400 MHz, CDCl_3_) *δ* 10.49 (br.s, 1H). 9.79 (s, 2H), 7.02 (d, *J* = 2.2 Hz, 2H). ^13^C NMR (100 MHz, CDCl_3_) *δ* 181.62, 135.83, 119.69.

**ESI-MS***m/z* (rel int): (neg) 122.0 ([M–H]^−^, 100).

### (4*S*,5*R*)-1′-Hydroxy-4-((*tert*-butyldimethylsilyl)oxy)-5-(((*tert*-butyldimethylsilyl)oxy)-methyl)-1′,4,4′,5-tetrahydro-3*H*-spiro[furan-2,3′-pyrrolo[2,1-*c*] [1,4] oxazine]-6′-carbaldehyde (**12a**)

To a solution of 1H-Pyrrole-2,5-dicarbaldehyde **11** (15 mg, 0.12 mmol) in dry THF (2 mL) under argon at 0 °C, sodium hydride 60 % in oil (4 mg, 0.1 mmol) was added. After 45 min at rt, the orange solution was cooled to 0 °C, and a solution of bromo alcohol **10a** (68 mg, 0.15 mmol) in dry THF (2 mL) was cautiously added. After 4 h at rt, the mixture was quenched with a saturated solution of NH_4_Cl, extracted with CH_2_Cl_2_ (4 mL × 2). The combined organic phase was washed with brine, dried over Na_2_SO_4_, filtered and evaporated to dryness. After purification by flash chromatography on silica gel, the lactol **12a** (nearly 1/1 diastereoisomers) was provided as pale yellow oil [10]. Yield: 22 mg (42 %).

Isomer A (54 %) ^1^H NMR (500 MHz, CD_3_COCD_3_) *δ* 11.23 (br.s, 1H), 9.55 (s, 1H), 6.94 (d, *J* = 2.6 Hz, 1H), 6.43 (dd, *J* = 3.5, 2.3 Hz, 1H), 6.02 (s, 1H), 4.52 (td, *J* = 5.7, 4.0 Hz, 1H), 4.15 (d, *J* = 8.5 Hz, 1H), 3.99 (d, *J* = 8.7 Hz, 1H), 3.93 (m, 1H), 3.69 (dd, *J* = 5.4, 1.3 Hz, 2H), 2.58–2.55 (m, 1H), 2.21–2.16 (m, 1H), 0.92–0.88 (overlapping s, 18H), 0.16–0.08 (overlapping s, 12H). ^13^C NMR (100 MHz, CD_3_COCD_3_) *δ* 180.01, 137.15, 134.71, 120.34, 112.89, 111.08, 98.09, 88.48, 74.72, 73.15, 64.43, 43.15, 26.28, 26.25, 26.13, 26.10, 18.90, 18.54, 18.47, −4.49, −4.57, −4.68, −4.69, −5.07, −5.17.

Isomer B (46 %) ^1^H NMR (400 MHz, CD_3_COCD_3_) *δ* 11.25 (br.s, 1H), 9.55 (s, 1H), 6,95 (d, *J* = 2.3 Hz, 1H), 6.45 (dd, *J* = 3.7, 2.3 Hz, 1H), 6.03 (s, 1H), 4.40 (dt, *J* = 8.1, 5.0 Hz, 1H), 4.10 (d, *J* = 8.5 Hz, 1H), 3.96–3.94 (m, 1H), 3.93 (m, 1H), 3.81 (dd, *J* = 11.5, 3.1 Hz, 1H), 3.75 (dd, *J* = 11.5, 3.9 Hz, 1H), 2.62–2.59 (m, 1H), 2.26–2.22 (m, 1H), 0.92–0.88 (overlapping s, 18H), 0.16–0.08 (overlapping s, 12H). ^13^C NMR (100 MHz, CD_3_COCD_3_) *δ* 180.01, 137.15, 134.71, 120.34, 112.43, 110.99, 98.17, 87.38, 74.29, 71.99, 63.05, 43.62, 26.28, 26.25, 26.13, 26.10, 18.90, 18.54, 18.47, −4.49, −4.57, −4.68, −4.69, −5.07, −5.17.

**ESI-MS***m/z* (rel int): (pos) 520 ([M + Na]^+^, 100).

### (4*S*,5*R*)-1′-Hydroxy-4-((triisopropylsilyl)oxy)-5-(((triisopropylsilyl)oxy)-methyl)-1′,4,4′,5-tetrahydro-3*H*-spiro[furan-2,3′-pyrrolo[2,1-*c*] [1,4] oxazine]-6′-carbaldehyde (**12b**)

The S_N_2 coupling between **11** and **14b** was similar to the above formation of **12a**, however, using Cs_2_CO_3_ as mild base [6]. **12b** (nearly 1/1 diastereoisomers) was afforded as colorless oil. Yield: 35 mg (60 %).

^1^H NMR (500 MHz, CDCl_3_) *δ* 9.53 (s, 1H), 9.40 (s, 1H), 6.91 (s, 1H), 6.40 (d, *J* = 2.7 Hz, 1H), 6.04 (s, 1H), 4.60–4.57 (m, 1H), 4.14–3.97 (m, 3H), 3.87–3.64 (m, 2H), 2.55–2.10 (m, 2H), 1.06 (s, 42H). Isomer A ^13^C NMR (100 MHz, CDCl_3_) *δ* 181.27, 132.86, 119.60, 110.68, 107.14, 97.02, 88.65, 75.44, 72.80, 63.92, 41.71, 18.07, 12.19, 12.00. Isomer B ^13^C NMR (100 MHz, CDCl_3_) *δ* 179.73, 135.64, 120.96, 112.54, 110.68, 107.14, 97.02, 88.20, 75.35, 65.39, 43.18, 18.07, 12.19, 12.00.

Assigned by comparison with compound **12a**, part of assignments were in some cases interchangeable.

### 1-Bromo-1,3-dideoxy-4,6-di-*O*-(triisopropylsilyl)-d-fructofuranose (**14a**)

The modified procedure for bromomethylation was as same as that of **10a** [9, 16], **14a** (nearly 1/1 diastereoisomers) was afforded as colorless oil. Yield: 32 mg (36 %).

Isomer A ^1^H NMR (400 MHz, CD_3_COCD_3_) *δ* 4.85 (br.s, 1H, OH), 4.57–4.52 (m, 1H), 4.10 (q, *J* = 4.4 Hz, 1H), 3.74–3.71 (m, 2H), 3.45 (d, *J* = 3.1 Hz, 2H), 2.50–2.44 (m, 1H), 2.03–1.99 (m, 1H), 1.03 (overlapping s, 42H). ^13^C NMR (100 MHz, CD_3_COCD_3_) *δ* 105.14, 88.06, 74.10, 64.04, 45.11, 38.30, 18.35, 12.62.

Isomer B ^1^H NMR (400 MHz, CD_3_COCD_3_) *δ* 5.11 (br.s, 1H, OH), 4.62–4.58 (m, 1H), 3.94 (q, *J* = 4.4 Hz, 1H), 3.74–3.71 (m, 2H), 3.67 (m, 1H), 3.52 (dd, *J* = 10.1, 8.2 Hz, 1H), 2.37–2.32 (m, 1H), 2.25–2.17 (m, 1H), 1.03 (overlapping s, 42H).^13^C NMR (100 MHz, CD_3_COCD_3_) *δ* 106.00, 89.20, 74.54, 65.11, 44.55, 39.86, 18.35, 12.76.

Assigned by comparison with compound **10a**, part of assignments were in some cases interchangeable.

### 1,3-Dideoxy-1-iodo-4,6-di-*O*-(triisopropylsilyl)-d-fructofuranose (**14b**)

The procedure for iodomethylation was similar to the reported one [9, 10], **14b** (nearly 1/1 diastereoisomers) was afforded as colorless oil. Yield: 41 mg (70 %).

Isomer A ^1^H NMR (400 MHz, CD_3_COCD_3_) *δ* 4.77 (br.s, 1H, OH), 4.59–4.55 (m, 1H), 4.10 (q, *J* = 4.4 Hz, 1H), 3.75–3.73 (m, 2H), 3.36 (s, 2H), 2.49–2.44(m, 1H), 2.14–2.10 (m, 1H), 1.06 (overlapping s, 42H). ^13^C NMR (100 MHz, CD_3_COCD_3_) *δ* 104.71, 88.36, 74.62, 64.18, 45.92, 18.26, 15.41, 12.75, 12.63.

Isomer B ^1^H NMR (400 MHz, CD_3_COCD_3_) *δ* 5.11 (br.s, 1H, OH), 4.62–4.58 (m, 1H), 3.98 (q, *J* = 4.4 Hz, 1H), 3.75–3.73 (m, 2H), 3.72 (m, 1H), 3.53 (q, *J* = 9.7 Hz, 1H), 2.40–2.36 (m, 1H), 2.30–2.26 (m, 1H), 1.06 (overlapping s, 42H). ^13^C NMR (100 MHz, CD_3_COCD_3_) *δ* 105.49, 89.26, 74.67, 65.02, 45.49, 18.26, 12.88, 12.75, 12.63.

Assigned by comparison with compound **10a**, part of assignments were in some cases interchangeable.

### Cyanomethyl Cinnamate (**18**)

To a solution of cinnamic acid (1.48 g, 10 mmol) in CH_2_Cl_2_ (4 mL) cooled to 0 °C, was added triethylamine (2.02 g, 20 mmol). After stirring 10 min at rt, chloroacetonitrile (1.13 g, 15 mmol) was added and the mixture was stirred overnight. The reaction mixture was quenched with ice-H_2_O mixture (3 mL) and the aqueous phase was extracted with CH_2_Cl_2_ (4 mL × 2). Combined organic layers were dried over anhydrous Na_2_SO_4_ and filtered. After evaporation of the solvents, the residue was purified by flash column chromatography to afford **18** as colorless oil [22]. Yield: 1.4 g (75 %).

^1^H NMR (400 MHz, CDCl_3_) *δ* 7.80 (d, *J* = 16.0 Hz, 1H), 7.62–7.35 (m, 5H), 6.46 (d, *J* = 16.0 Hz, 1H), 4.86 (s, 2H). ^13^C NMR (100 MHz, CDCl_3_) *δ* 165.26, 147.90, 133.77, 131.25, 129.17, 128.53, 115.35, 48.54.

### (*E*)-1-Hydroxy-7-phenylhept-6-ene-2,5-dione (**19**)

To a solution of the cyanomethylbenzoate **18** (187 mg, 1 mmol) and Ti(O-*i*-Pr)_4_ (313 mg, 1.1 mmol) in dry Et_2_O (5 mL) was added a solution of EtMgBr (0.7 mL, 2.1 mmol, 3 M in Et_2_O) dropwise at 0 °C under nitrogen. After the addition of Grignard reagent, the mixture was allowed to warm up to rt. About 1 h later, the turbid yellow mixture was quenched with water (1 mL). EtOAc (10 mL) and 1 M HCl were added carefully to obtain two clear phases. The aqueous phase was extracted with EtOAc (5 mL × 2). The combined organic phases were washed with saturated aqueous NaHCO_3_ and dried by Na_2_SO_4_. After evaporation of the solvents, the residue was purified by flash chromatography to afford **19** as white solid: mp 72–74 °C [22]. Yield: 153 mg (70 %).

^1^H NMR (400 MHz, CDCl_3_) *δ* 7.59 (d, *J* = 16.3 Hz, 1H), 7.57–7.54 (m, 2H), 7.41–7.40 (m, 3H), 6.76 (d, *J* = 16.2 Hz, 1H), 4.39 (d, *J* = 4.7 Hz, 2H), 3.11 (t, *J* = 6.2 Hz, 2H), 3.06 (t, *J* = 4.8 Hz, 1H), 2.75 (t, *J* = 6.2 Hz, 2H).^13^C NMR (100 MHz, CDCl_3_) *δ* 208.88, 198.00, 143.45, 134.35, 130.84, 129.13, 128.49, 125.60, 68.43, 34.33, 31.99.

### (*E*)-(5-Styryl-1H-pyrrol-2-yl)methanol (**20**)

A microwave reaction vessel was equipped with a stir bar and charged with diketone **19** (44 mg, 0.2 mmol), NaOAc (20 mg, 0.24 mmol) and NH_4_OAc (54 mg, 0.7 mmol) in 1 mL of EtOH, the resulting mixture in the reaction vessel was sealed and allowed to stir for 5 min at rt, then heated by microwaves at 40 °C for 12 min in a CEM-microwave reactor. The reactants were concentrated in vacuo, 1 mL H_2_O were added, the aqueous phase was extracted with EtOAc (3 mL × 2), the combined organic layers were dried over anhydrous Na_2_SO_4_ and filtered. After evaporation of the solvents, the pure residue **20** was checked by NMR directly. Yield: quantitative conversion.

^1^H NMR (400 MHz, CDCl_3_) *δ* 9.17 (s, 1H), 7.41–7.26 (m, 5H), 6.91 (d, *J* = 16.4 Hz, 1H), 6.69 (d, *J* = 16.4 Hz, 1H), 6.23 (s, 1H), 6.11 (s, 1H), 4.61 (s, 2H), 4.19 (s, 1H). ^13^C NMR (100 MHz, CDCl_3_) *δ* 137.60, 132.69, 131.67, 128.75, 127.01, 125.95, 123.66, 119.11, 109.32, 108.77, 58.03.

## Electronic supplementary material

The biogenesis studies with troublesome Amadori rearrangement, protecting groups screened for lactones and related trials with glitches, general procedure for the synthesis of key intermediates and their characterization data for NMR (^1^H and ^13^C NMR, DPET, HSQC and HMBC) and MS spectra along with corresponding copies.

Below is the link to the electronic supplementary material.

Supplementary material 2 (DOC 14363 kb)
