# Supplementary material for: Cascade N-Alkylation/Hemiacetalization for Facile Construction of the Spiroketal Skeleton of Acortatarin Alkaloids with Therapeutic Potentiality in Diabetic Nephropathy
Source: Nat Prod Bioprospect. 2014 Dec 16;5(1):37–45. doi: 10.1007/s13659-014-0049-8 (PMC4327998; doi:10.1007/s13659-014-0049-8)
Supplement: Supplementary file 1 — Supplementary material 2 (DOC 14363 kb) [file 13659_2014_49_MOESM1_ESM.doc]

***Supporting Information***

**Cascade N-alkylation/hemiacetalization for Facile Construction of**

**the Spiroketal Skeleton of Acortatarin Alkaloids**

**with Therapeutic Potentiality in Diabetic Nephropathy**

**Pei Cao,^a^ Zhen-Jie Li,^a^ Wen-Wu Sun,^a,b^ Shashwat Malhotra,^c,d^ Yuan-Liang Ma,^a^**

**Bin Wu,***^a^ Virinder S. Parmar***^c^**

^a^ State Key Laboratory of Phytochemistry and Plant Resources in West China, Kunming Institute of Botany,

Chinese Academy of Sciences, Kunming 650201, P. R. China.

^b^ University of Chinese Academy of Sciences, Beijing 100049, P. R. China.

^c^ Bioorganic Laboratory, Department of Chemistry, University of Delhi, Delhi 110007, India.

^d^ Institute of Chemistry and Biochemistry, Freie Universität Berlin, Takustrasse 3, Berlin 14195, Germany.

Fax:+86-871-6521-6960; E-mail: wubin@mail.kib.ac.cn.

E-mail: virparmar@gmail.com.

**Table of Contents**

Biogenesis studies with troublesome Amadori rearrangement S3

Protecting groups screened for lactones and related trials S6

Synthetic procedures of key intermediates and their NMR/MS data S8

Copies of NMR/MS spectra for key intermediates S15

**Biogenesis studies with troublesome Amadori rearrangement**

Triggered by the proposed biogenesis of acortatarins, disconnection of the pyrrole ring would give rise to amino sugar intermediates **S3** and 1,4-diketo **S5**. We tried to firstly access the building block **S3a** and **S3b** through Amadori rearrangement [1a] of 3-Deoxy-D-glucosone (**S4a**) [2a] and D-glucose (**S4b**), further selective protection of free hydroxyl groups if necessary, and Paal–Knorr cyclization with 1,4-dicarbonyl unit **S5 [**3a] to construct the pyrrole motif, final screening of the conditions besides classical acidic catalyst for the crucial spiroketalization at the late stage (Scheme S1). Since the large scale-adaptable protocols are described for the straightforward conversion of HMF **S6** to versatile 1,4-diketo **S5** [3a], it seems that this route would go smoothly if we could obtain **S3a** and **S3b** in hand. Unluckily, after an overview of the Amadori rearrangement (AR), it allows the formation of 1-aminodeoxyketoses as a mixture of pyranose and furanose salt forms [1b,1c] **S3a/S3a’** and **S3b/S3b’** from the respective aldose **S4a** and **S4b** (in accordance with the isomers of acortatarin A isolated in nature), or occurs as the sole pyranoid isomer **S3a’** and **S3b’** [1d]. A very valuable reaction sequence can not be optimized without the requirement for *O*-protecting-group manipulations, for primary and secondary [1e] hydroxyl groups attached at C-6 or C-4, 6 of aldoses **S4a** and **S4b** to orchestrate furanoid conformation at C-5, which is also not feasible with excellent preparative yields.

**Scheme S1** Proposed biogenesis of acortatarins A and B *via* Amadori rearrangement and Paal-Knorr cyclization.

To quickly assess the viability of our proposed synthesis, we launched a SciFinder search for related natural products. Deoxy sugars are important components of a variety of small-molecule therapeutics [2b]. Astonishingly, chemoenzymatic method for a wide range of substituted monosaccharides still resorted to the traditional Barton-McCombie deoxygenation protocol [2a,2b]. 3-Deoxy-D-glucose **S4a** could be generated from the hydrolysis and rearrangement of diacetone-3-deoxy-α-D-glucofuranose **S7** which was in turn obtained from radical-based reduction of thionocarbonate **S8** [2a], in general, also adaptable to the iodide **S9** [2c]. Costly and highly toxic organotin hydrides were often used as hydrogen donors associated with substantial drawbacks. Considerable efforts have been made to devise more acceptable replacements such as silanes [2c] or organoboranes [2d]. The catalytic phosphoramidite transfer [2e] and visible-light-mediated photoredox catalysis [2f] have pioneered to highlight the significant challenges in this region. Since the efficient and cost-effective deoxygenation processes are still in demand especially for preparing bulk chemicals avoiding scale-up problems [2j], we preferred iodide **S9** to thionocarbonate **S8** as key intermediate.

(I) H_2_SO_4_, CuSO_4_, acetone, 0 °C to rt, 73%. (II) PPh_3_-I_2_-imidazole, toluene, reflux, 25%. (III) (CF_3_SO_2_)_2_O or MsCl, Et_3_N, DMAP, CHCl_3_, 0 °C to rt. (IV) *n*-Bu_4_NI, toluene, reflux, 66% over two steps for **S10** to **S9** through **S11a**. (V) H_2_, Pd/C, EtOH, rt to 50 °C, 88% for **S9** to **S13**, 94% for **S14** to **S15**. (VI) 0.1% H_2_SO_4_/H_2_O, reflux. (VII) DBU, rt to 100 °C, 71% over two steps for **S10** to **S14** through **S11b**.

**Scheme S2** The facile synthesis tried for 3-deoxy sugars from D-glucose.

We began our synthesis with D-glucose **S4b** as the starting material which was converted to its diacetonide **S10** with 73% yield using concentrated H_2_SO_4_ [4a]. The straightforward iodination subjected to PPh_3_-I_2_-imidazole system gave iodide **S9** in low yield (25%), similar to that reported [5a]. Two-step conversion through the S_N_2 reaction between triflate **S11a** and *n*-Bu_4_NI was adopted in 66% overall yield [5b]. However, less active mesylate **S11b** failed to furnish iodide **S9** with the change of solvents (toluene, THF, DME, DMF) and temperature (reflux at different boiling points) along with salts (LiI, NaI, KI, *n*-Bu_4_NI). Because reductive dehalogenation utilizes dissolved H_2_ as an electron donor in the presence of a commercial palladium catalyst progress rapidly [6a], we firstly tried the deiodination of **S9** by Pd/C-H_2_ system from room temperature to 50 °C for 12 hours. Surprisingly, the crude NMR of reaction exhibited signals of one product with almost quantitatively regioselective monohydrolysis, which was confirmed to be **S13** after further purification. As one special complement to the selective cleavage of 5,6-acetonide in weak acid, this may be induced by the HI released. At this point, we found that Et_3_N had already been brought to scavenge the HI byproduct during the Pd/C-catalyzed conversion of iodide **S9** to **S7** successfully [6b]. We anticipated that either **S13** or **S7** could be delivered easily with subsequent hydrolysis/rearrangement [2a] to finish **S4a** ultimately. Then we turned to explore the hydrogenation of olefin **S14** for the synthesis of diastereomer **S16 [**7]. As slight modifications to the procedures published, triflate **S11a** [5b] was substituted by readily accessible mesylate **S11b**, though the following E2 elimination could not be effected nicely by treatment with DBU at room temperature. To our delight, full transformation to olefin **S14 [**7] did occur at 100 °C overnight, which was verified by the crude NMR of reaction only with usual organic/aqueous extraction. After further purification by short flash column, the saturation of olefinic double bond in **S14** uniformly provided pure diacetone-3-deoxy-α-D-gulofuranose **S15** (about 70% yield over three steps) [7]. The final conventional hydrolysis [2a] to **S16** would not be difficult. Hereto, our methodology of deiodination sought and olefination optimized were expected for the application in extensive synthesis of related deoxy sugars in near future (Scheme S2).

The technically viable reaction channels from carbohydrates to nitrogen heterocycles have been exploited [3a]. To appraise the practicability of our speculation for biomass derived 1,4-diketo **S5**, direct transformation of D-fructose **S17** to HMF **S6** performed exceptionally well with catalytical NaHSO_4_ [3a] without more optimization. The hydroxymethyl group in HMF **S6** was protected in one-pot operation as silyl ether **S18** with 45% yield [3b]. Reduction of the aldehyde followed by protection of the resulting alcohol **S20** to afford tetrahydropyranyl ether **S21** [3b], and oxidation of the furan **S21** with anhydrous *m*-CPBA ensured the *cis*-enone **S22** in 51% yield over two steps [3a,3b]. The final hydrogenation of *cis*-enone **S22** was implemented quantitatively by Pd/C-H_2_ [7] or TiCl_3_ [3a] to give unsymmetrical 1,4-diketone **S23**, which was confirmed by HPLC and NMR data. In contrast, reduction with Zn/AcOH [3b] proceeded slowly. However, due to the volatility of **S23**, we could not collect the pure form even with modest yield at the last stage (Scheme S3).

(I) NaHSO_4_, DMSO, 110 °C. (II) TBDMSCl or BzCl, Et_3_N, DMAP, CHCl_3_, 0 °C to rt, 45% over two steps for **S17** to **S18** through **S6**. (III) (a) NaBH_4_, MeOH, 0 °C to rt; (b) DHP, CHCl_3_, PPTS, rt, 90% over two steps for **S18** to **S21** through **S20**. (IV) *m*-CPBA, CH_2_Cl_2_, rt, 51%. (V) H_2_, Pd/C, EtOH, rt. (VI) Ethylene glycol, tartaric acid, MgSO_4_, toluene, reflux, quantitative conversion.

**Scheme S3** The convenient synthesis tested for 1,4-diketones from D-fructose.

Thus, more stable benzoyl ester **S19** was introduced. In consideration of the atom economy during the course of transformation of aldehyde group, we decided to retain it as cyclic acetal in furan **S24** employing tartaric acid as mild catalyst [4b]. The oxidation of **S24** with *m*-CPBA finished enedione **S25** [3a,3b]. It was facile to get 1,4-diketone **S26** by the testified Pd/C-H_2_ system [7] ultimately. In all the key oxidation steps mentioned above, other oxidant systems such as Br_2_-acetone-H_2_O [3a], NaClO_2_-NaH_2_PO_4_-*t*-BuOH–H_2_O [3c], CAN-MeCN-H_2_O [3d] were also screened, they gave similar or less conversion monitored by TLC (Scheme S3).

So far, we could deduce from the experiments for the synthesis of 3-deoxy sugars **S4a/S16** derived from D-glucose **S4b** and 1,4-diketones **S23/S26** generated from D-fructose **S17** that it is possible to produce them only from the natural carbohydrates. But the chemoselective Amadori rearrangements require further studies in detail. We stopped our biogenesis investigation temporarily when we were attracted by the short cut to acortatarins A and B designed by Tan, which urged us to devise a more full-fledged synthetic plan.

**Protecting groups screened for lactones and related trials**

We attempted to focus the synthesis of acortatarin A with the real system involving determination of the optimal protecting groups for the bromomethylation and N-alkylation. D-ribono-1,4-lactone **7a** underwent ring expansion during the acetalization and kelalzation induced by concentrated HCl [4c]. The mild acidic media (*p*-TsOH [4d,4e], CSA [4f], I_2_ [4f]) combined with acetone, 2,2-dimethoxypropane and benzaldehyde dimethylacetal were tested with negligible *O*-isopropylidenation. However, uncyclic acetal **S27** was isolated in reasonable yield over two steps from **4a** catalyzed by I_2_ only in refluxing THF. Lactone **7a** was also converted to disilyl ether **S28** upon treatment with commercially available 1,3-dichloro-1,1,3,3-tetraisopropyldisiloxane (TIPDSCl), and THP ether **S29** by 3,4-dihydro-2H-pyran in the presence of catalytic PPTS [3b] in good overall yields (each about 70%), respectively. However, both of these two groups were cleaved fully under the standard bromomethylation (Scheme S4).

(I) Benzaldehyde dimethylacetal, I_2_, THF, reflux, 60% over two steps for **4a** to **S27** through **7a**. (II) TIPDSCl, imidazole, DMAP, DMF, 0 °C to rt, 70% over two steps. (III) DHP, THF, PPTS, rt, 70% over two steps. (IV) MOMCl, DIPEA or DBU, *n*-Bu_4_NI, THF, 0 °C to rt, 63% over two steps. (V) 2,4,6-tris(allyloxy)-1,3,5-triazine, TfOH, dioxane, rt to 100 °C, 60% over two steps. (VI) TIPSOTf, lutidine, DMAP, DMF, 0 °C to rt, 70% over two steps. (VII) CH_2_Br_2_ or CH_2_I_2_, *n*-BuLi, THF or toluene, −82 °C, 36% for **8d** to **14a**, 70% for **8d** to **14b**. (VIII) Cs_2_CO_3_, MeCN, rt, 60%. (IX) NaBH_4_, MeOH, 0 °C to rt, quantitative conversion.

**Scheme S4** Protecting groups screened for lactones and related trials

While the unsatisfactory trials established preliminarily that one less-hindered protection group in **7a** and **7b** with stability against strong bases would be beneficial, the functional compatibility of the protecting groups for this aim was considered to be ethers which in general are stable to basic conditions, including methoxymethyl (MOM), benzyl and allyl ethers. In fact, the desired MOM ether **S30** was contaminated by a small amount of isomer which was probably due to the partial racemization in the case of either DIPEA or DBU [8]. The simple protocol for decarboxylative allylation of **7a** by allyl-*t*-butylcarbonate combined with Pd(OAc)_2_/PPh_3_ [9a] or Pd(PPh_3_)_4_ [9b] only afforded complex mixtures of products. As compared to similar protection with 2,4,6-tris(benzyloxy)-1,3,5-triazine (TriBOT), *O*-allylation with 2,4,6-tris(allyloxy)-1,3,5-triazine (TriAOT) was somewhat slower with unexpected excellent regioselectivity and moderate yield to afford mono allylated ether **S31** (no bis-allylated products appeared even when heated to 100 °C in dioxane under microwave condition, and very less possible racemic/isomeric impurities produced could be removed by recrystallization).

We anticipated that **3a** and **3b** in the context as the halomethylated equivalents of S**3a** and S**3b** would offer us with the other choice, to generate S**3a** and S**3b** by aminolysis for the Paal–Knorr cyclization with diketone **S5**. However, the aminolysis of **14a** and **14b** in saturated or diluted aqueous ammonia was conducted without any success to give amino alcohol **S32**, either under pressure in a sealed vessel or by conventional/microwave-assisted conditions at different temperatures (rt to 100 °C) with several co-solvents (THF, MeCN, dioxane). Lactol **12b** was obtained similar to **12a** (Cs_2_CO_3_/MeCN, 60% yield without further optimization). The reduction of aldhyde group in **12b** to alcohol **S33** by NaBH_4_ proceeded very smoothly. The following lactol ring-cleavage and rearrangement to morpholine motif **22** was examined fruitlessly through a variety of Lewis acids (*p*-TsOH, CSA, TFA, I_2_) (Scheme S4).

**References:**

1 (a) K. Gallas, G. Pototschnig, F. Adanitsch, A. E. Stütz and T. M. Wrodnigg, *Beilstein J. Org. Chem.* 2012, **8**, 1619; (b) W. W. Weeks, M. P. Campos and S. Moldoveanu, *J. Agric. Food. Chem.* 1995, **43**, 2247; (c) J. Levi, Z. Cheng, O. Gheysens, M. Patel, C. T. Chan, Y. Wang, M. Namavari and S. S. Gambhir, *Bioconjugate Chem.* 2007, **18**, 628; (d) H. Tian, X. She, L. Shu, H. Yu and Y. Shi, *J. Am. Chem. Soc.* 2000, **122**, 11551; (e) H. E. Zaugg, *J. Org. Chem.* 1961, **26**, 603.

2 (a) S. V. Chetyrkin, W. Zhang, B. G. Hudson, A. S. Serianni and P. A. Voziyan, *Biochemistry* 2008**,** **47**, 997; (b) J. C. Lewis, S. Bastian, C. S. Bennett, Y. Fu, Y. Mitsuda, M. M. Chen, W. A. Greenberg, C. Wong and F. H. Arnold, *Proc. Natl. Acad. Sci. U.S.A.* 2009, **106**, 16550; (c) A. Studer, S. Amrein, *Angew. Chem., Int. Ed. Engl.* 2000, **39**, 3080; (d) D. A. Spiegel, K. B. Wiberg, L. N. Schacherer, M. R. Medeiros and J. L. Wood, *J. Am. Chem. Soc.* 2005, **127**, 12513; (e) P. A. Jordan and S. J. Miller, *Angew. Chem., Int. Ed. Engl.* 2012, **51**, 2907; (f) J. D. Nguyen, E. M. D’Amato, J. M. R. Narayanam and C. R. Stephenson, *J. Nat. Chem.* 2012, **4**, 854; (j) H. S. Park, H. Y. Lee and Y. H. Kim, *Org. Lett.* 2005, **7**, 3187.

3 (a) F. W. Lichtenthaler, A. Brust and E. Cuny, *Green Chem*. 2001, **3**, 201; (b) T. Okada, K. Sakaguchi, T. Shinada and Y. Ohfune, *Tetrahedron Lett.* 2011, **52**, 5744; (c) S. P. Annangudi, M. Sun and R. G. Salomon, *Synlett* 2005, 1468; (d) B. Alcaide, P. Almendros, R. Carrascosa and M. R. Torres, *Eur. J. Org. Chem.* 2010, 823.

4 (a) C. Adelwöhrer, T. Takano, F. Nakatsubo and T. Rosenau, *Biomacromolecules* 2009, **10**, 2817; (b) T.-J. Lu, J.-F. Yang and L.-J. Sheu, *J. Org. Chem.* 1995, **60**, 2931; (c) S.-Y. Han, M. M. Joullié, N. A. Petasis, J. Bigorra, J. Corbera, J. Font and R. M. Ortuño, *Tetrahedron* 1993, **49**, 349; (d) A. R. Chamberlin, M. Dezube, S. H. Reich and D. J. Sal1, *J. Am. Chem. Soc.* 1989, **111**, 6247; (e) L. S. Hegedus, L. Geisler, A. G. Riches, S. S. Salman and G. Umbricht, *J. Org. Chem.* 2002, **67**, 7649; (f) R. Panchadhayee and A. K. Misra, *J. Carbohydr. Chem.* 2008, **27**, 148.

5 (a) J. Boivin and V. T. Nguyen, *Beilstein J. Org. Chem.* 2007, **3**, No. 45; (b) G.-X. Yu, D. R. Tyler and B. P. Branchaud, *J. Org. Chem.* 2001, **66**, 5687.

6 (a) W. W. Jr. McNab and R. Ruiz, M. Reinhard, *Environ. Sci. Technol.* 2000, **34**, 149; (b) M. Hamerníková, J. Havlíček, H. Votavová and K. Kefurt, *Collect. Czech. Chem. Commun.* 2002, **67**, 622.

7 V. U. Pawar, S. Ghosh, B. A. Chopade and V. S. Shinde, *Bioorg. Med. Chem. Lett.* 2010, **20**, 7243.

8 K. Y. Tsang, M. A. Brimble and J, B. Bremner, *Org. Lett.* 2003, **5**, 4425.

9 (a) E. J. Stoner, M. J. Peterson, M. S. Allen, J. A. DeMattei, A. R. Haight, M. R. Leanna, S. R. Patel, D. J. Plata, R. H. Premchandran and M. Rasmussen, *J. Org. Chem.* 2003, **68**, 8847; (b) J. M. Kraus, H. C. Gits and R. B. Silverman, *Tetrahedron Lett.* 2012, **53**, 1319.

- - - 1. **Experimental section:**

General procedures for the synthesis of key intermediates and their characterization data for NMR and MS spectra.

**1-Bromo-1,3-dideoxy-4,6-di-*O*-(*tert-*butyldimethylsilyl)-D-fructofuranose (10a).**

Table S1. Key coorelations of HSQC and HMBC for compound **10a**.

| No. | δ_C_ | δ_H_ (HSQC) | HMBC |
| --- | --- | --- | --- |
| A |  | -OH, 4.90 (br.s, 1H) | 104.94, 44.84, 38.51 |
|  | 104.94 |  |  |
|  | 87.34 | 4.05 (q, *J* = 4.4 Hz, 1H) | 104.94, 73.57, 44.84 |
|  | 73.57 | 4.41 (dt, *J* = 6.7, 4.4 Hz, 1H) | 104.94, 63.42 |
|  | 63.42 | 3.68-3.66 (m, 2H) | 104.94, 87.34, 73.57, 44.84 |
|  | 44.84 | 2.49 (dd, *J* = 13.3, 6.9 Hz, 1H), 1.99 (dd, *J* = 13.3, 4.4 Hz, 1H) | 104.94, 87.34, 73.57, 38.51 |
|  | 38.51 | 3.49 (d, *J* = 2.1 Hz, 2H) | 104.94, 44.84 |
| B |  | -OH, 5.10 (br.s, 1H) | 105.58, 44.44, 39.66 |
|  | 105.58 |  |  |
|  | 88.67 | 3.87 (dd, J = 9.2, 4.8 Hz, 1H) | 105.58, 73.79, 44.44 |
|  | 73.79 | 4.50 (dt, J = 5.8, 4.8 Hz, 1H) | 105.58, 64.55 |
|  | 64.55 | 3.68-3.66 (m, 2H) | 105.58, 88.67, 73.79, 44.44 |
|  | 44.44 | 2.25 (qd, J = 13.3, 5.5 Hz, 2H) | 105.58, 88.67, 73.79, 39.66 |
|  | 39.66 | 3.65 (d, J = 1.7 Hz, 1H), 3.56 (d, J = 10.3 Hz, 1H) | 105.58, 44.44 |

**1-Bromo-1-deoxy-3,4,6-tri-*O*-(*tert-*butyldimethylsilyl)-D-fructofuranose (10b).**

Table S2. Key coorelations of HSQC and HMBC for compound **10b**.

| No. | δ_C_ | δ_H_ (HSQC) | HMBC |
| --- | --- | --- | --- |
| A |  | -OH, 4.49 (d, *J* = 1.9 Hz, 1H) | 105.78, 79.60, 36.41 |
|  | 105.78 |  |  |
|  | 87.11 | 3.83-3.79 (m, 1H) | 105.78, 79.60, 79.51, 64.51 |
|  | 79.60 | 4.22-4.21 (m, 1H) | 105.78, 87.11, 79.51, 36.41 |
|  | 79.51 | 4.27-4.26 (m, 1H) | 105.78, 87.11, 79.60, 64.51 |
|  | 64.51 | 3.71 (d, *J* = 6.0 Hz, 1H), 3.66 (d, *J* = 6.0 Hz, 1H) | 105.78, 87.11, 79.51 |
|  | 36.41 | 3.69 (d, *J* = 10.5 Hz, 1H), 3.58 (d, *J* = 10.5 Hz, 1H) | 105.78, 79.60 |
| B |  | -OH, 4.67 (d, *J* = 1.0 Hz, 1H) | 106.75, 82.58, 34.88 |
|  | 106.75 |  |  |
|  | 87.92 | 4.08-4.07 (m, 1H) | 106.75, 82.58, 79.64, 64.38 |
|  | 82.58 | 4.10-4.09 (m, 1H) | 106.75, 87.92, 79.64, 64.38 |
|  | 79.64 | 4.24-4.23 (m, 1H) | 106.75, 87.92, 82.58, 64.38 |
|  | 64.38 | 3.71 (d, *J* = 6.0 Hz, 1H), 3.66 (d, *J* = 6.0 Hz, 1H) | 106.75, 87.92, 79.64 |
|  | 34.88 | 3.54 (dd, *J* = 10.3, 1.1 Hz, 1H), 3.47 (d, *J* = 10.3 Hz, 1H) | 106.75, 82.58 |

**(4*S*,5*R*)-1’-Hydroxy-4-((*tert-*butyldimethylsilyl)oxy)-5-(((*tert-*butyldimethylsilyl)oxy)-methyl)-1’,4,4’,5-tetrahydro-3*H*-spiro[furan-2,3’-pyrrolo[2,1-*c*][1,4]oxazine]-6’-carbaldehyde (12a).**

Table S3. Key coorelations of HSQC and HMBC for compound **12a**.

| No. | δ_C_ | δ_H_ (HSQC) | HMBC |
| --- | --- | --- | --- |
| A |  | -OH, 11.23 (br.s, 1H) |  |
|  | 180.01 | 9.55 (s, 1H) | 134.71 |
|  | 137.15 |  |  |
|  | 134.71 |  |  |
|  | 120.34 | 6.94 (d, *J* = 2.6 Hz, 1H) | 180.01, 137.15, 134.71, 111.08 |
|  | 112.89 |  |  |
|  | 111.08 | 6.43 (dd, *J* = 3.5, 2.3 Hz, 1H) | 137.15, 134.71, 120.34, 98.09 |
|  | 98.09 | 6.02 (s, 1H) | 137.15, 111.08 |
|  | 88.48 | 3.93 (m, 1H) | 112.89, 73.15, 64.43, 43.15 |
|  | 74.72 | 4.15 (d, *J* = 8.5 Hz, 1H), 3.99 (d, *J* = 8.7Hz, 1H) | 112.89, 98.09, 43.15 |
|  | 73.15 | 4.52 (td, *J* = 5.7, 4.0 Hz, 1H) | 112.89, 64.43 |
|  | 64.43 | 3.69 (dd, *J* = 5.4, 1.3 Hz, 2H) | 88.48, 73.15 |
|  | 43.15 | 2.58-2.55 (m, 1H), 2.21-2.16 (m, 1H) | 112.89, 88.48, 74.72, 73.15 |
| B |  | -OH, 11.25 (br.s, 1H) |  |
|  | 180.01 | 9.55 (s, 1H) | 134.71 |
|  | 137.15 |  |  |
|  | 134.71 |  |  |
|  | 120.34 | 6,95 (d, J = 2.3 Hz, 1H) | 180.01, 137.15, 134.71, 110.99 |
|  | 112.43 |  |  |
|  | 110.99 | 6.45 (dd, J = 3.7, 2.3 Hz, 1H) | 137.15, 134.71, 120.34, 98.17 |
|  | 98.17 | 6.03 (s, 1H) | 137.15, 110.99 |
|  | 87.38 | 3.96-3.94 (m, 1H) | 112.43, 71.99, 63.05, 43.62 |
|  | 74.29 | 4.10 (d, J = 8.5 Hz, 1H), 3.93 (m, 1H) | 112.43, 98.17, 87.38, 43.62 |
|  | 71.99 | 4.40 (dt, J = 8.1, 5.0 Hz, 1H) | 112.43, 63.05 |
|  | 63.05 | 3.81 (dd, J = 11.5, 3.1 Hz, 1H), 3.75 (dd, J = 11.5, 3.9 Hz, 1H) | 87.38, 71.99 |
|  | 43.62 | 2.62-2.59 (m, 1H), 2.26-2.22 (m, 1H) | 112.43, 87.38, 74.29, 71.99 |

**1,2:5,6-Di-*O*-isopropylidene-α-D-glucofuranose (S10).**

D-Glucose **S4b** (2.0 g, 11.1 mmol) was stirred in dry acetone (50 mL) with CuSO_4_ (5.0 g) and a catalytic amount of sulfuric acid for 24 h. The solution was neutralized with concentrated aqueous ammonia and filtered. The remainder was washed thoroughly with acetone and dichloromethane. The combined organic phases were concentrated to about 15 mL in vacuo, and water (100 mL) was added. The mixture was stirred vigorously for 5 min and repeatedly extracted with dichloromethane to obtain the crude diacetonide **S10**. After purification of the crude diacetonide by recrystallization in petroleum ether and EtOAc, pure compound **S10** was obtained (2.1g, 73% yield) [4a].

^1^H NMR (500 MHz, CDCl_3_) δ 5.95 (d, *J* = 3.5 Hz, 1H), 4.54 (d, *J* = 3.5 Hz, 1H), 4.33 (ddd, *J* = 7.8, 6.1, 5.4 Hz, 1H), 4.31 (d, *J* = 2.9 Hz, 1H), 4.17 (dd, *J* = 9.2, 6.3 Hz, 1H), 4.07 (dd, *J* = 7.6, 2.6 Hz, 1H), 3.98 (dd, *J* = 8.6, 5.4 Hz, 1H), 2.54 (d, *J* = 3.3 Hz, 1H), 1.50 (s, 3H), 1.44 (s, 3H), 1.36 (s, 3H), 1.32 (s, 3H).

**3-Deoxy-3-iodo-1,2:5,6-di-*O*-isopropylidene-α-D-glucofuranose (S9).**

A mixture of diacetonide **S10** (0.52 g, 2.0 mmol), triphenylphosphine (5.74 g, 2.2 mmol) and imidazole (0.27 g, 4.0 mmol) in toluene (10 mL) was heated to 80°C with stirring under nitrogen. Iodine (0.56 g, 2.2 mmol) was added slowly and the reaction mixture was stirred at reflux for 1.5 h. The hot mixture was poured into a flask containing saturated aqueous NaHSO_3_ solution (4 mL) and stirred for 10 min. EtOAc (20 mL) was added, and the organic layer was washed with brine, dried over Na_2_SO_4_, and concentrated. Silica gel column chromatography purification afforded iodide **S9** (0.18 g, 25% yield) [5a].

Iodide **S9** was also obtained for two steps by the alternative method reported using triflate **S11a** and *n*-Bu_4_NI in refluxing anhydrous toluene (0.24 g, 66% overall yield for two steps) [5b].

^1^H NMR (500 MHz, CDCl_3_): δ 5.81 (d, *J* = 3.4 Hz, 1H), 4.60 (t, *J* = 3.8 Hz, 1H), 4.32 (m, 1H), 4.25 (dd, *J* = 10.0, 3.7 Hz, 1H), 4.13 (dd, *J* = 8.2, 6.0 Hz, 1H), 4.06 (dd, *J* = 8.2, 6.9 Hz, 1H), 3.76 (dd, *J* = 10.0, 4.4 Hz, 1H), 1.56 (s, 3H), 1.49 (s, 3H), 1.37 (s, 6H).

**3-*O*-Trifluoromethanesulfonyl-1,2:5,6-di-*O*-isopropylidene-α-**D**-allofuranoside (S11a).**

To a 250 mL three-neck-round-bottom flask was equipped with a magnetic stir bar, pyridine (1.34 g, 17.0 mmol) and 100 mL of CH_2_Cl_2_ were added. The solution was cooled to −10 °C (ice-acetone bath). Triflic anhydride (2.70 mL, 4.16 g, 15.4 mmol) dissolved in 20 mL of CH_2_Cl_2_, was added from one dropping funnel over a period of 20 min. A thick white suspension was formed and shaking of the reaction vessel by hand was necessary. After the addition of triflic anhydride, the reaction mixture was allowed to stir 15 more min, then diacetonide **S10** (2.0 g, 7.7 mmol) dissolved in 20 mL of CH_2_Cl_2_ was added dropwise via the other dropping funnel over a period of 30 min. The reaction mixture was then stirred for 2 h. The slurry was poured into 100 mL ice-H_2_O mixture. The organic layer was separated and the aqueous portion was extracted twice with CH_2_Cl_2_ (50 mL x 2). The combined organic portions were dried over anhydrous Na_2_SO_4_ and the solvent was removed by rotary evaporation. Due to the instability of sugar triflate intermediate **S11a**, the thick colorless liquid obtained above was used for the next reaction immediately [5b].

**3-Deoxy-l,2-*O*-isopropylidene-α-D-gIucofuranose (S13).**

A solution of iodide **S9** (64 mg, 0.2 mmol) and 5% Pd/C (12 mg, 0.006 mmol) in 2 ml EtOH was stirred at rt for 1 h then heated to 50 °C overnight under H_2_ atmosphere (at balloon pressure). After completion of the reaction, it was filtered through a celite pad and washed with MeOH. Solvent evaporation under reduced pressure and flash column purification afforded the pure pale yellow syrup **S13** (36 mg, 88% yield) [6a, 6b].

^1^H NMR (400 MHz, CDCl_3_) δ 5.86 (d, *J* = 3.6 Hz, 1H), 4.77 (t, *J* = 4.0 Hz, 1H), 4.28 (dt, *J* = 10.8, 4.2 Hz, 1H), 4.04 (dt, *J* = 7.0, 3.7 Hz, 1H), 3.79 (dd, *J* = 11.3, 3.3 Hz, 1H), 3.65 (dd, *J* = 11.3, 6.8 Hz, 1H), 3.48 (s, 1H), 2.10 (s, 1H), 2.06 (dd, *J* = 13.5, 4.5 Hz, 1H), 1.88 (ddd, *J* = 13.5, 11.1, 4.7 Hz, 1H), 1.50 (s, 3H), 1.32 (s, 3H).

**3-Deoxy-1,2;5,6-di-*O*-isopropylidene-α-D-erythro-hex-3-enofuranose (S14).**

To the oily crude methanesulfonate **S11b** (170 mg, 0.5 mmol) [5b] in round-bottom flask was added 1,8-Diazabicyclo[5.4.0]undec-7-ene (DBU, 380 mg, 2.5 mmol)) dropwise, the solution was stirred at rt for 1 h then heated to 100 °C for 24 to 36 h. After that, 2 mL H_2_O and 10 mL diethyl ether were added to the reaction system and stirred vigorously for 15 minutes, the upper ether phase was divided, the aqueous phase was extracted with diethyl ether twice again (5 mL x 2). The combined organic phases were washed with brine and dried over anhydrous Na_2_SO_4_. The solution was concentrated in vacuo followed by purification using flash silica gel chromatography to give the desired olefin **S14** (86.1 mg, 71% yield over two steps) [7].

^1^H NMR (400 MHz, CDCl_3_) δ 6.08 (d, *J* = 5.3 Hz, 1H), 5.30 (m, 1H), 5.24 (m, 1H), 4.58 (dd, *J* = 6.7, 5.8 Hz, 1H), 4.15 (dd, *J* = 8.4, 6.8 Hz, 1H), 3.97 (dd, *J* = 8.4, 5.7 Hz, 1H), 1.47 (s, 6H), 1.44 (s, 3H), 1.39 (s, 3H).

**3-Deoxy-1,2:5,6-di-*O*-isopropylidene-α-D-gulo-1,4-furanose (S15).**

A solution of olefin **S14** (96 mg, 0.4 mmol) and 5% Pd/C (29 mg, 0.014 mmol) in 3 ml MeOH was stirred overnight under H_2_ atmosphere (at balloon pressure). After completion of the reaction, it was filtered through a celite pad and washed with ethyl acetate. Solvent evaporation under reduced pressure afforded white crystalline solid **S15**, mp 72-74°C (90 mg, 94% yield) [7].

^1^H NMR (400 MHz, CDCl_3_) δ 5.76 (d, *J* = 3.6 Hz, 1H), 4.69 (d, *J* = 8.9 Hz, 1H), 4.40 (dd, *J* = 14.8, 6.9 Hz, 1H), 4.07 (td, *J* = 8.4, 3.9 Hz, 1H), 4.01 (t, *J* = 7.3 Hz, 1H), 3.57 (t, *J* = 7.5 Hz, 1H), 2.21-2.14 (m, 1H), 1.78 (dd, *J* = 14.2, 3.4 Hz, 1H), 1.53 (s, 3H), 1.41 (s, 3H), 1.33 (s, 3H), 1.29 (s, 3H).

**5-((*tert*-butyldimethylsilyloxy)methyl)furan-2-carbaldehyde (S18).**

D-Fructose **S17** (1.8 g, 10 mmol) and NaHSO_4_ (0.6 g, 0.5 mmol) were mixed in 10 mL DMSO. The mixture was heated at 120 °C for 2 h. The color of the mixture turned to dark brown. The reactants were cooled to rt, quenched with water, extracted with methyl isobutyl ketone (15 mL x 2), The MIBK layer was washed with brine then dried over anhydrous Na_2_SO_4_, filtered, and concentrated under reduced pressure. The crude frualdehyde **S6** was put for next step without purification. To a solution of 5-HMF **S6** in CHCl_3_ (20 mL) were added TBDMSCl (1.6 g, 10 mmol), imidazole (1.4 g, 20 mmol) and DMAP (122 mg, 1 mmol) at 0 °C. The reaction mixture was stirred at rt for 12 h, quenched with saturated NH_4_Cl and extracted with EtOAc. The organic layer was washed with brine, dried over anhydrous Na_2_SO_4_ and filtered. The filtrate was concentrated in vacuo and purified by flash chromatography to give **S18** (1.1 g, 45% yield) [3a, 3b].

^1^H NMR (400 MHz, CDCl_3_) δ 9.58 (s, 1H), 7.20 (d, *J* = 3.5 Hz, 1H), 6.47 (d, *J* = 3.5 Hz, 1H), 4.73 (s, 2H), 0.91 (s, 9H), 0.10 (s, 6H). ^13^C NMR (100 MHz, CDCl_3_) δ 177.69, 161.60, 152.30, 109.55, 58.75, 25.91, 18.47, –5.25.

**(5-((*tert-*butyldimethylsilyloxy)methyl)furan-2-yl)methanol (S20).**

To the solution of **S18** (240 mg, 1 mmol) in 3 mL MeOH/THF (1/2) was added NaBH_4_ (23 mg, 0.6 mmol) at 0 °C. After stirring at rt for 1 h, excessive NaBH_4_ was decomposed with a few drops of AcOH. The residue was concentrated in vacuo, which was added saturated NaHCO_3_ and extracted with EtOAc. The organic layer was washed with brine, dried over anhydrous Na_2_SO_4_ and filtered. The filtrate was concentrated in vacuo to give the pure residue **S20**, which was used for the next step immediately [3b].

^1^H NMR (400 MHz, CDCl_3_) δ 6.22 (d, *J* = 2.8 Hz, 1H), 6.17 (d, *J* = 2.8 Hz, 1H), 5.30 (s, 1H), 4.62 (s, 2H), 4.57 (s, 2H), 0.90 (s, 9H), 0.08 (s, 6H). ^13^C NMR (100 MHz, CDCl_3_) δ 154.54, 153.69, 108.61, 108.11, 58.38, 57.73, 26.02, 18.57, –5.09.

***tert*-Butyldimethyl((5-((tetrahydro-2H-pyran-2-yloxy)methyl)furan-2-yl)methoxy)silane (S21).**

To a solution of the residue **S20** in CH_2_Cl_2_ (2 mL) were added DHP (0.44 mL, 4.8 mmol) and PPTS (25 mg, 0.1 mmol) at 0 °C. After stirring at rt for 8 h, the mixture was quenched with saturated NaHCO_3_ and extracted with CH_2_Cl_2_. The organic layer was washed with brine, dried over anhydrous Na_2_SO_4_ and filtered. The filtrate was concentrated in vacuo to give a crude residue, which was purified by flash column chromatography to give **S21** as oily compound (293 mg, 90% yield for two steps) [3b].

^1^H NMR (500 MHz, CDCl_3_) δ 6.25 (d, *J* = 3.0 Hz, 1H), 6.17 (d, *J* = 3.0 Hz, 1H), 4.72 (t, *J* = 3.4 Hz, 1H), 4.63 (m, 3H), 4.46 (d, *J* = 12.9 Hz, 1H), 3.93-3.88 (m, 1H), 3.56-3.52 (m, 1H), 1.85-1.51 (m, 6H), 0.90 (s, 9H), 0.08 (s, 6H). ^13^C NMR (125 MHz, CDCl_3_) δ 154.84, 151.46, 110.06, 107.96, 97.39, 62.11, 60.89, 58.50, 30.56, 26.03, 25.63, 19.34, 18.54, –5.08.

**(*Z*)-1-(*tert*-Butyldimethylsilyloxy)-6-(tetrahydro-2H-pyran-2-yloxy)hex-3-ene-2,5-dione (S22).**

To a solution of **S21** (260 mg, 0.8 mmol) in CH_2_Cl_2_ (3 mL) was added 70% *m*-CPBA (246 mg, 1 mmol). After stirring at rt for 10 h, the mixture was filtered through a thin silica gel pad to remove the 3-chlorobenzoic acid. The mixture was washed with saturated NaHCO_3_, brine, dried over anhydrous MgSO_4_ and filtered. The filtrate was concentrated in vacuo and purified by flash column chromatography to give **S22** as colorless syrup (195 mg, 51% yield) [3b].

^1^H NMR (400 MHz, CDCl_3_) δ 6.52-6.26 (m, 2H), 5.58-5.47 (m, 4H), 4.96 (m, 1H), 3.83 (t, *J* = 10.1 Hz, 1H), 3.58-3.52 (m, 1H), 1.86-1.55 (m, 6H), 0.90 (s, 9H), 0.15 (s, 6H). ^13^C NMR (100 MHz, CDCl_3_) δ 199.62, 197.31, 130.12, 130.07, 96.95, 85.72, 85.14, 62.29, 29.98, 25.69, 25.29, 18.74, 1.17, –5.08.

**1-(*tert-*Butyldimethylsilyloxy)-6-(tetrahydro-2H-pyran-2-yloxy)hexane-2,5-dione (S23).**

A solution of olefin **S22** (171 mg, 0.5 mmol) and 5% Pd/C (58 mg, 0.028 mmol) in 3 ml MeOH was stirred overnight under H_2_ atmosphere (at balloon pressure). After completion of the reaction, it was filtered through celite pad and washed with ethyl acetate. Solvent evaporation under reduced pressure afforded the diketone **S23** which was too volatile to be collected, only crude ^1^H NMR was checked [3b].

^1^H NMR (400 MHz, CDCl_3_) δ 5.43-5.41 (m, 4H), 4.93 (s, 1H), 3.87-3.81 (m, 1H), 3.58-3.52 (m, 1H), 2.67 (s, 4H), 1.88-1.61 (m, 6H), 0.90 (s, 9H), 0.13 (s, 6H).

**(*3S,5R*)-3-Hydroxy-5-*O*-(methoxy(phenyl)methyl)-D-ribonolactone (S27).**

The synthesis of lactone followed the modified precedure in the text [Araújo, A. C.; Rauter, A. P.; Nicotra, F.; Airoldi, C.; Costa, B.; Cipolla, L. *J. Med. Chem.* **2011**, *54*, p1266. & Wichai, U.; Woski, S. A. *Org. Lett.* **1999**, *1*, p1173. & Block, E.; Dikarev, E.V.; Glass, R. S.; Jin, J.; Li, B.; Li X.; Zhang, S.-Z. *J. Am. Chem. Soc.* **2006**, *128*, p14949]. The ketalization followed the literature procedure in refluxing THF [4f]. The oily compound was afforded (151 mg, 60% yield over two steps).

^1^H NMR (400 MHz, CDCl_3_) δ 7.46 (d, *J* = 6.0 Hz, 2H), 7.25 (d, *J* = 6.0 Hz, 3H), 5.52 (s, 1H), 4.31 (dd, *J* = 10.4, 4.7 Hz, 1H), 4.07-4.02 (m, 1H), 3.73 (s, 3H), 3.71-3.59 (m, 2H), 2.91 (dd, *J* = 15.9, 5.5 Hz, 1H), 2.75 (dd, *J* = 15.8, 6.6 Hz, 1H). ^13^C NMR (100 MHz, CDCl_3_) δ 172.46, 137.47, 129.15, 128.41, 126.22, 101.14, 78.19, 71.35, 66.26, 52.30, 38.39.

**EI-MS** *m/z* (rel int): 252 (M^+^, 100).

**3,5-*O*-((1,1,3,3-Tetraisopropyl)disiloxanediyl)-2-deoxy-D-ribono-1,4-lactone (S28).**

The synthesis of lactone followed the modified precedure in the text [Araújo, A. C.; Rauter, A. P.; Nicotra, F.; Airoldi, C.; Costa, B.; Cipolla, L. *J. Med. Chem.* **2011**, *54*, p1266. & Wichai, U.; Woski, S. A. *Org. Lett.* **1999**, *1*, p1173. & Block, E.; Dikarev, E.V.; Glass, R. S.; Jin, J.; Li, B.; Li X.; Zhang, S.-Z. *J. Am. Chem. Soc.* **2006**, *128*, p14949]. The protection by disilyl(TIPDSCl) followed the known precedure already cited in the text [Wichai, U.; Woski, S. A. *Org. Lett.* **1999,** *1*, p1173]. The oily compound was afforded (262 mg, 70% yield over two steps).

^1^H NMR (400 MHz, CDCl_3_) δ 4.63 (dd, *J* = 16.1, 8.0 Hz, 1H), 4.20 (dt, *J* = 6.6, 3.6 Hz, 1H), 4.13 (dd, *J* = 12.3, 3.5 Hz, 1H), 3.93 (dd, *J* = 12.3, 6.5 Hz, 1H), 2.85 (dd, *J* = 17.3, 8.0 Hz, 1H), 2.70 (dd, *J* = 17.3, 9.3 Hz, 1H), 1.07-1.02 (m, 28H). ^13^C NMR (100 MHz, CDCl_3_) δ 173.14, 84.92, 69.74, 62.37, 37.94, 17.55, 17.39, 17.35, 16.97, 14.43, 13.37, 13.23, 12.93, 12.59.

**ESI-MS** *m/z* (rel int): (pos) 397 ([M+Na]^+^, 100).

**1,1,3,3-Tetraisopropyldisiloxan-1,3-diol (cleaved fully from S28).**

The bromomethylation followed the modified precedure in the text [Bessières, B.; Morin, C. *J. Org. Chem.* **2003**, *68*, p4100. & Tite, T.; Tomas, L.; Docsa, T.; Gergely, P.; Kovensky, J.; Gueyrard, D.; Wadouachi, A. *Tetrahedron Lett.* **2012**, *53*, p959. & Hou, D.; Taha H. A.; Lowary, T. L. *J. Am. Chem. Soc.* **2009**, *131*, p12937]. The cleaved oily compound was afforded with NMR and MS data identical to those in the reference [Grabovskii, S. A.; Kabal'nova, N. N.; Shereshovets, V. V.; Chatgilialoglu, C. *Organometallics*. **2002**, *21*, p3506] (104 mg, 75% yield).

^1^H NMR (400 MHz, CD_3_COCD_3_) δ 4.91 (br.s, 2H), 1.05 (d, *J* = 1.9 Hz, 12H), 1.03 (d, *J* = 1.9 Hz, 12H). 0.88 (m, 4H). ^13^C NMR (100 MHz, CD_3_COCD_3_) δ 17.66, 17.61, 14.08.

**EI-MS** *m/z* (rel int): 278 (M^+^, 100), 235 (M^+^–43), 207.

**2-Deoxy-3,5-di-*O*-tetrahydro-2H-pyran-2-yloxyl-D-ribonolactone (S29).**

The synthesis of lactone followed the modified precedure in the text [Araújo, A. C.; Rauter, A. P.; Nicotra, F.; Airoldi, C.; Costa, B.; Cipolla, L. *J. Med. Chem.* **2011**, *54*, p1266. & Wichai, U.; Woski, S. A. *Org. Lett.* **1999**, *1*, p1173. & Block, E.; Dikarev, E.V.; Glass, R. S.; Jin, J.; Li, B.; Li X.; Zhang, S.-Z. *J. Am. Chem. Soc.* **2006**, *128*, p14949]. The protection by THP ether followed the known precedure [3b]. The oily compound was afforded (210 mg, 70% yield over two steps).

^1^H NMR (500 MHz, CDCl_3_) δ 4.70-4.62 (m, 2H), 4.57-4.46 (m, 2H), 3.99-3.89 (m, 1H), 3.81-3.72 (m, 2H), 3.63-3.48 (m, 3H), 2.94-2.82 (m, 1H), 2.62-2.45 (m, 1H), 1.79-1.66 (m, 4H), 1.57-1.51 (m, 8H). ^13^C NMR (125 MHz, CDCl_3_) δ 176.05, 99.70, 98.33, 85.33, 84.09, 74.15, 73.87, 67.70, 66.37, 63.01, 62.58, 36.91, 36.10, 30.78, 30.37, 25.32, 19.52.

**2-Deoxy-3,5-di-*O*-methoxymethoxyl-D-ribonolactone (S30).**

The synthesis of lactone followed the modified precedure in the text [Araújo, A. C.; Rauter, A. P.; Nicotra, F.; Airoldi, C.; Costa, B.; Cipolla, L. *J. Med. Chem.* **2011**, *54*, p1266. & Wichai, U.; Woski, S. A. *Org. Lett.* **1999**, *1*, p1173. & Block, E.; Dikarev, E.V.; Glass, R. S.; Jin, J.; Li, B.; Li X.; Zhang, S.-Z. *J. Am. Chem. Soc.* **2006**, *128*, p14949]. The protection by MOM ether followed the known precedure [8]. The oily compound was afforded (139 mg, 63% yield over two steps).

^1^H NMR (400 MHz, CDCl_3_) δ 4.84-4.70 (m, 1H), 4.67-4.58 (m, 4H), 4.41-4.38 (m, 1H), 3.79-3.70 (m, 2H), 3.37 (s, 3H), 3.34 (s, 3H), 2.91 (dd, *J* = 18.1, 7.1 Hz, 1H), 2.59-2.51 (m, 1H). ^13^C NMR (100 MHz, CDCl_3_) δ 175.46, 96.69, 96.00, 93.67, 93.47, 92.25, 91.16, 84.33, 84.27, 84.19, 74.56, 66.98, 55.93, 55.62, 36.31.

**(*3S,5R*)-5-*O*-Allyl-3-hydroxy-D-ribonolactone (S31).**

The synthesis of lactone followed the modified precedure in the text [Araújo, A. C.; Rauter, A. P.; Nicotra, F.; Airoldi, C.; Costa, B.; Cipolla, L. *J. Med. Chem.* **2011**, *54*, p1266. & Wichai, U.; Woski, S. A. *Org. Lett.* **1999**, *1*, p1173. & Block, E.; Dikarev, E.V.; Glass, R. S.; Jin, J.; Li, B.; Li X.; Zhang, S.-Z. *J. Am. Chem. Soc.* **2006**, *128*, p14949]. The allylation followed the known precedure already cited in the text [Yamada, K.; Fujita, H.; Kunishima, M. *Org. Lett.* **2012**, *14*, p5026]. The oily compound was afforded (103 mg, 60% yield over two steps).

^1^H NMR (400 MHz, CDCl_3_) δ 5.89-5.79 (m, 1H), 5.34-5.18 (m, 2H), 4.57-4.47 (m, 2H), 4.04-3.93 (m, 2H), 3.65 (qd, *J* = 10.7, 3.4 Hz, 2H), 2.95 (dd, *J* = 18.0, 6.8 Hz, 1H), 2.47 (dd, *J* = 18.0, 2.7 Hz, 1H). ^13^C NMR (100 MHz, CDCl_3_) δ 175.91, 133.94, 117.89, 86.24, 72.75, 69.98, 69.49, 38.52.


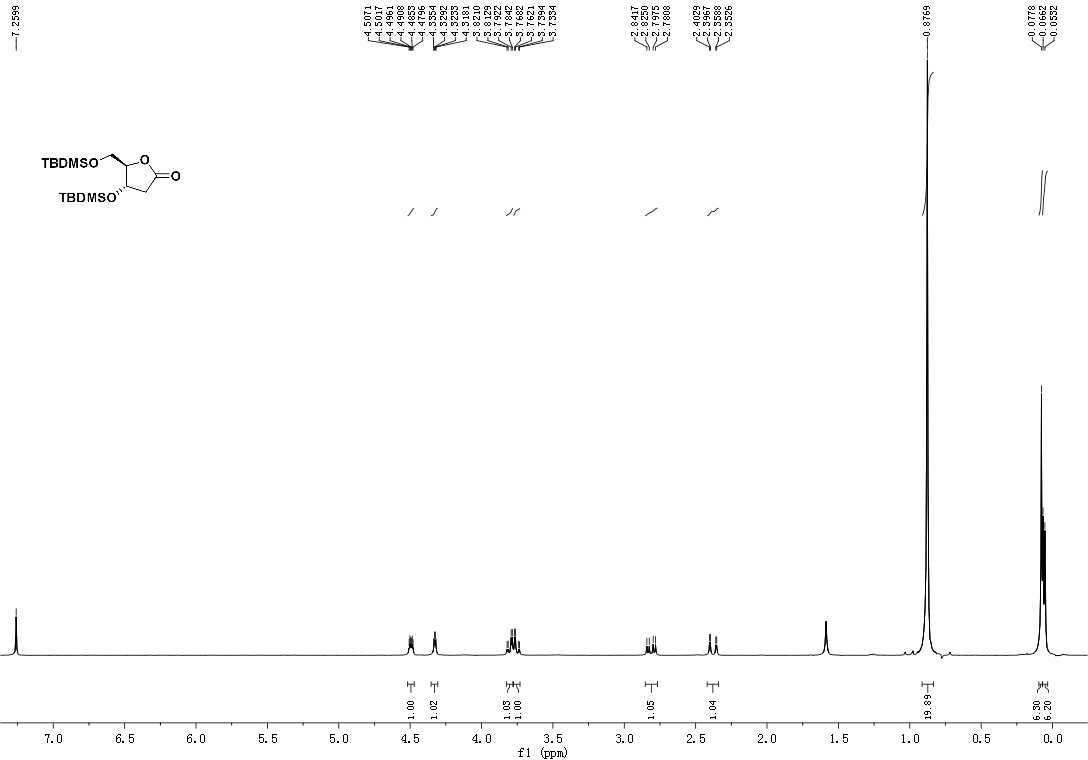


^1^H NMR spectra for **8a** (400 MHz, CDCl_3_)


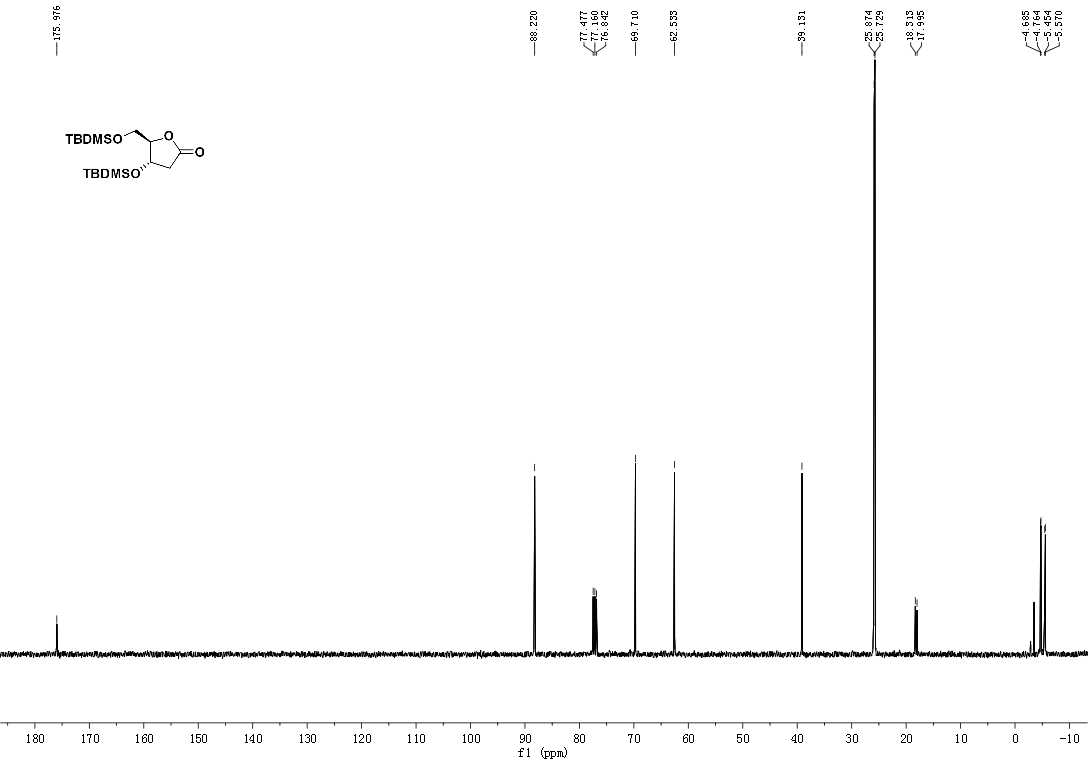


^13^C NMR spectra for **8a** (100 MHz, CDCl_3_)


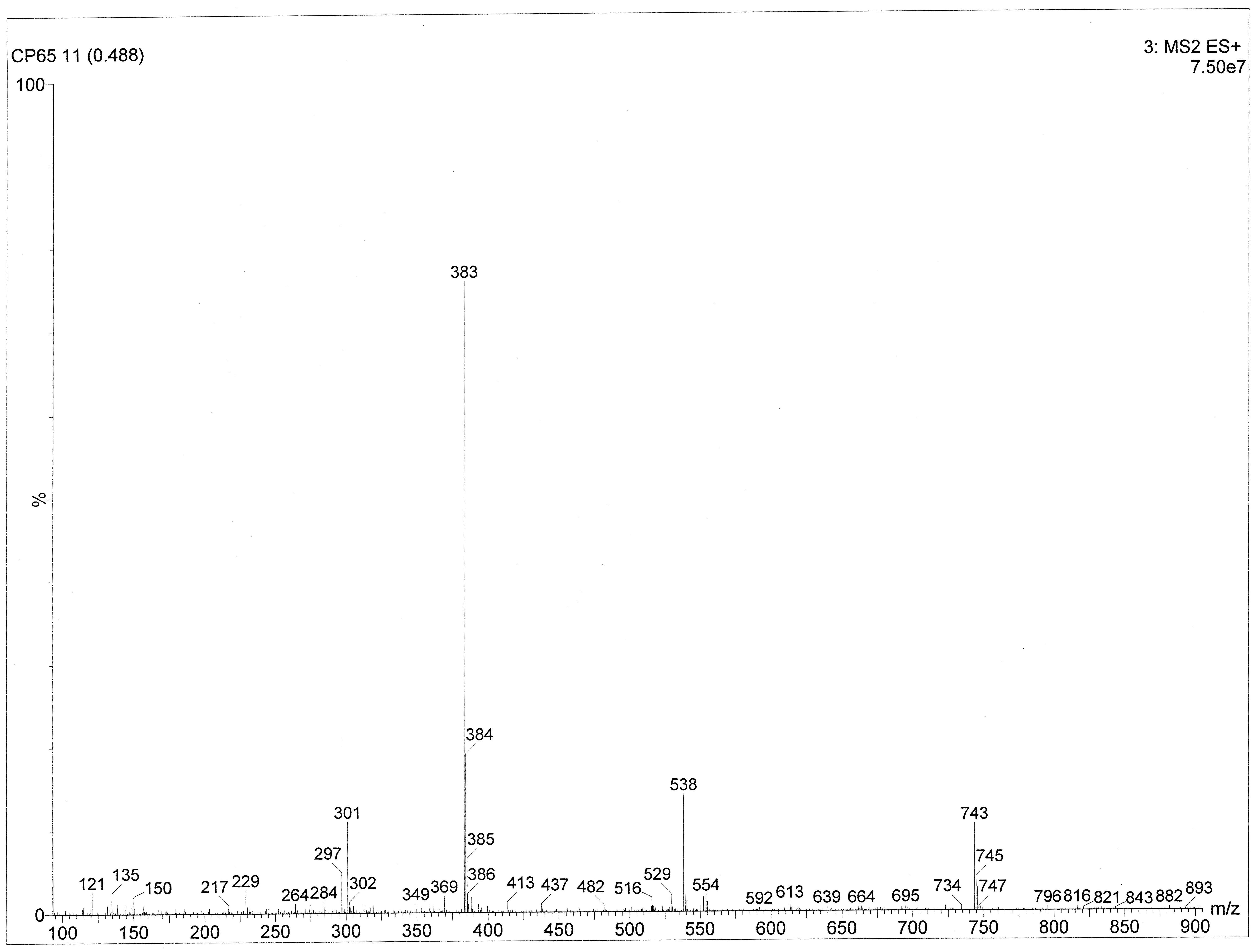


ESI-MS spectra for **8a**


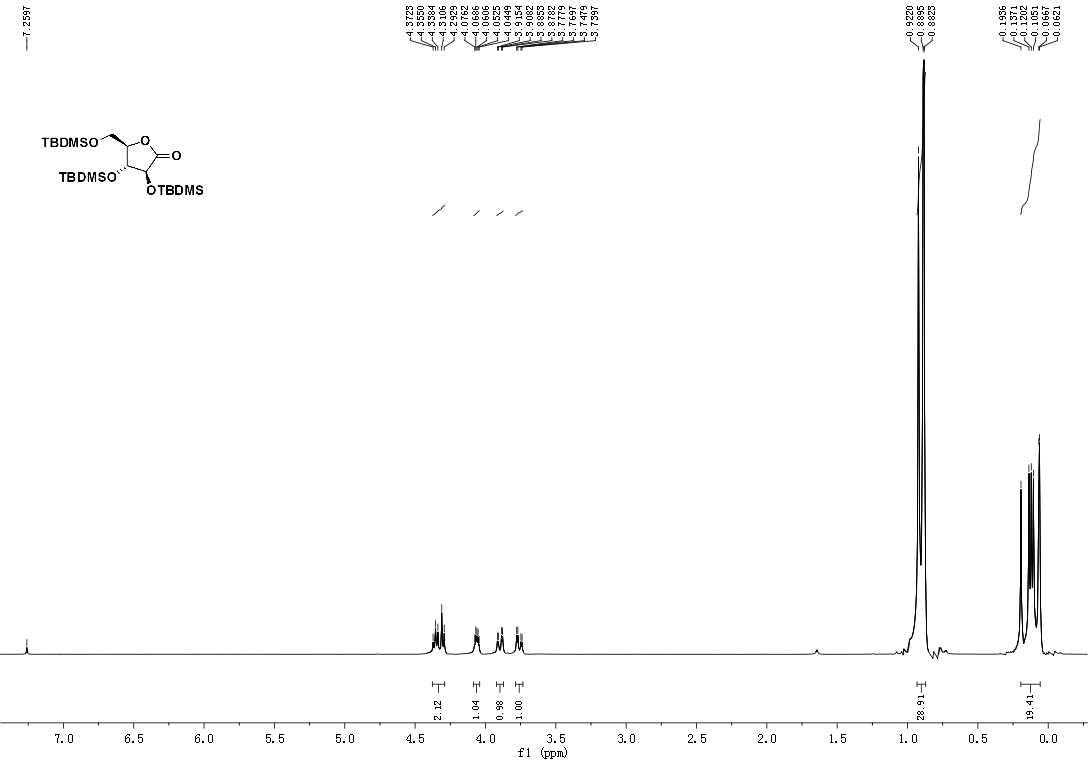


^1^H NMR spectra for **8b** (400 MHz, CDCl_3_)


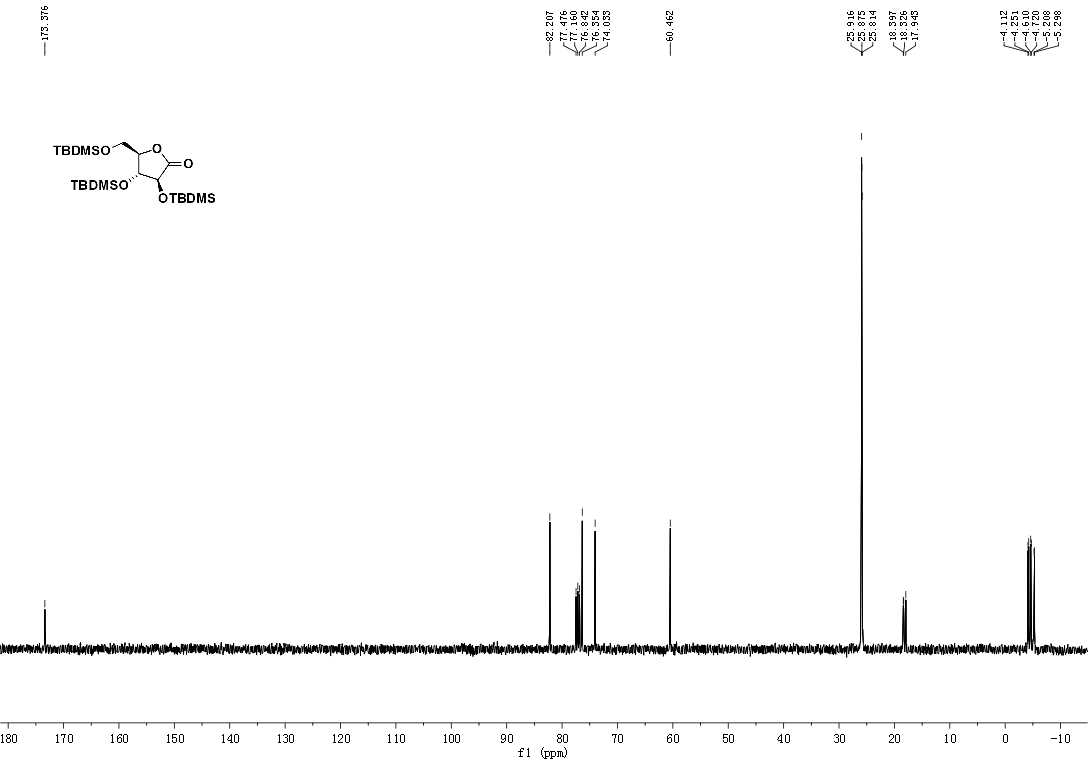


^13^C NMR spectra for **8b** (100 MHz, CDCl_3_)


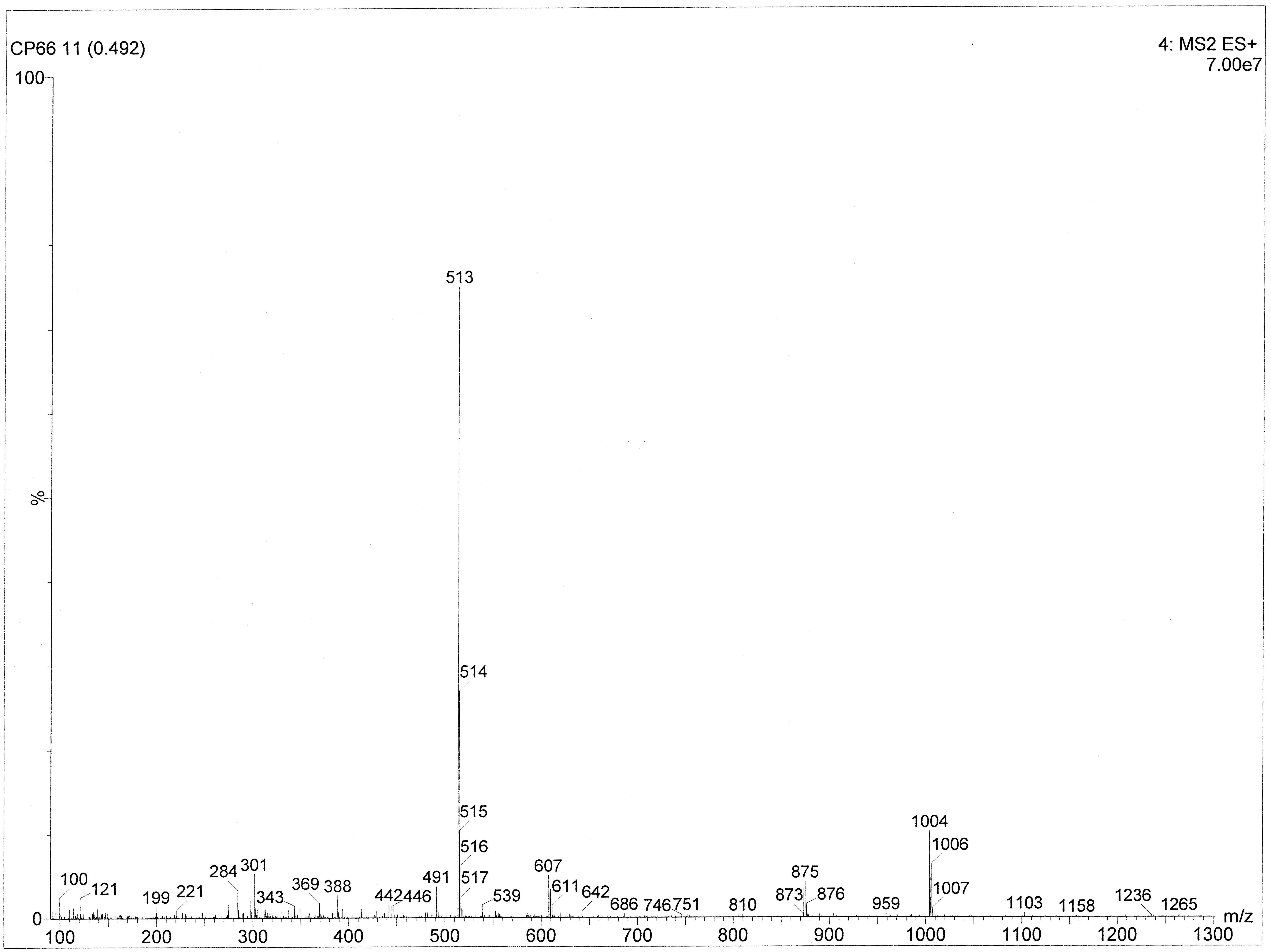


ESI-MS spectra for **8b**


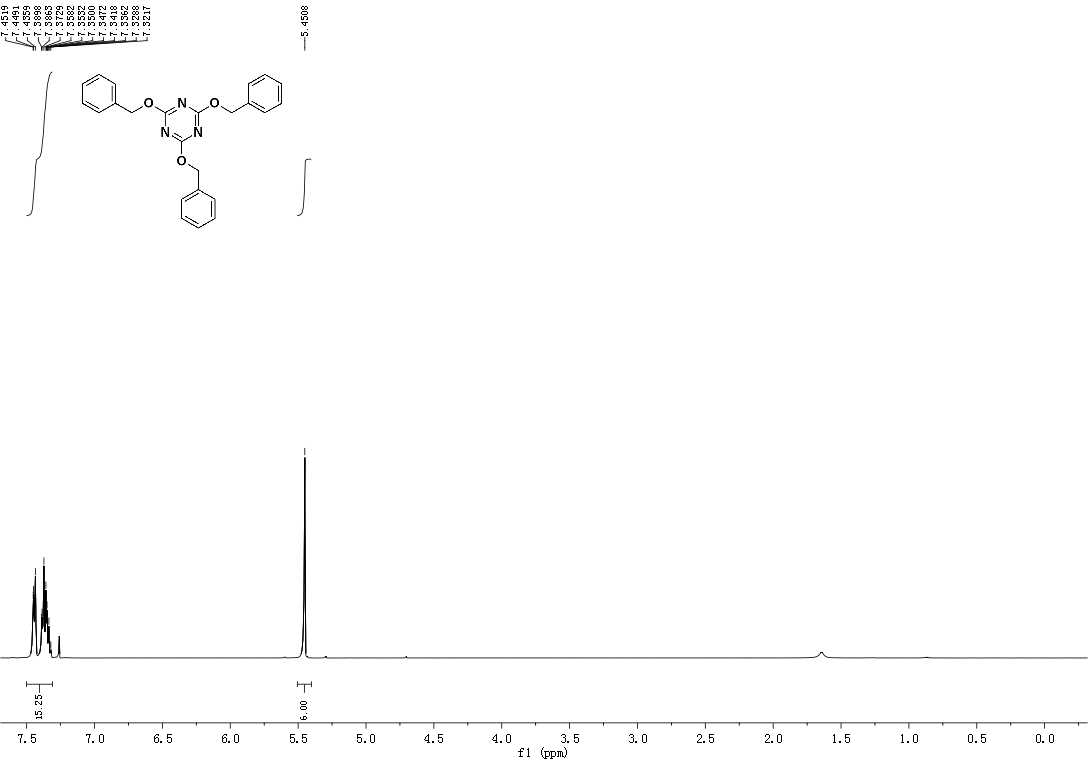


^1^H NMR spectra for **TriBOT** (500 MHz, CDCl_3_)


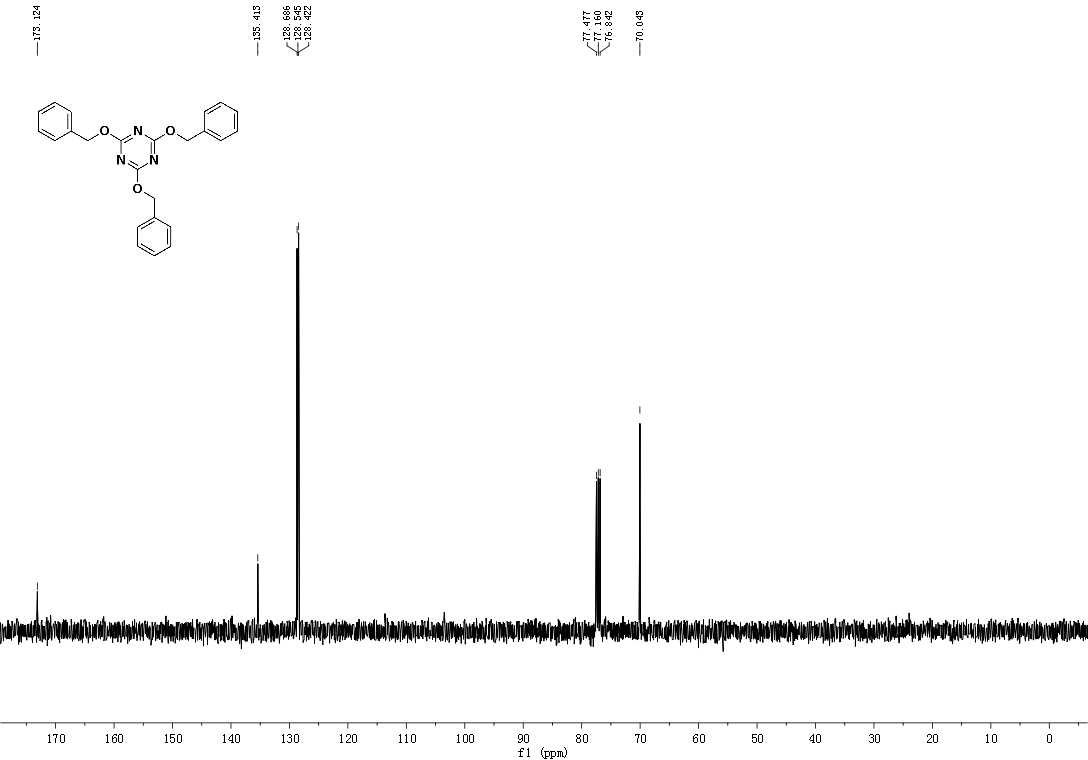


^13^C NMR spectra for **TriBOT** (100 MHz, CDCl_3_)


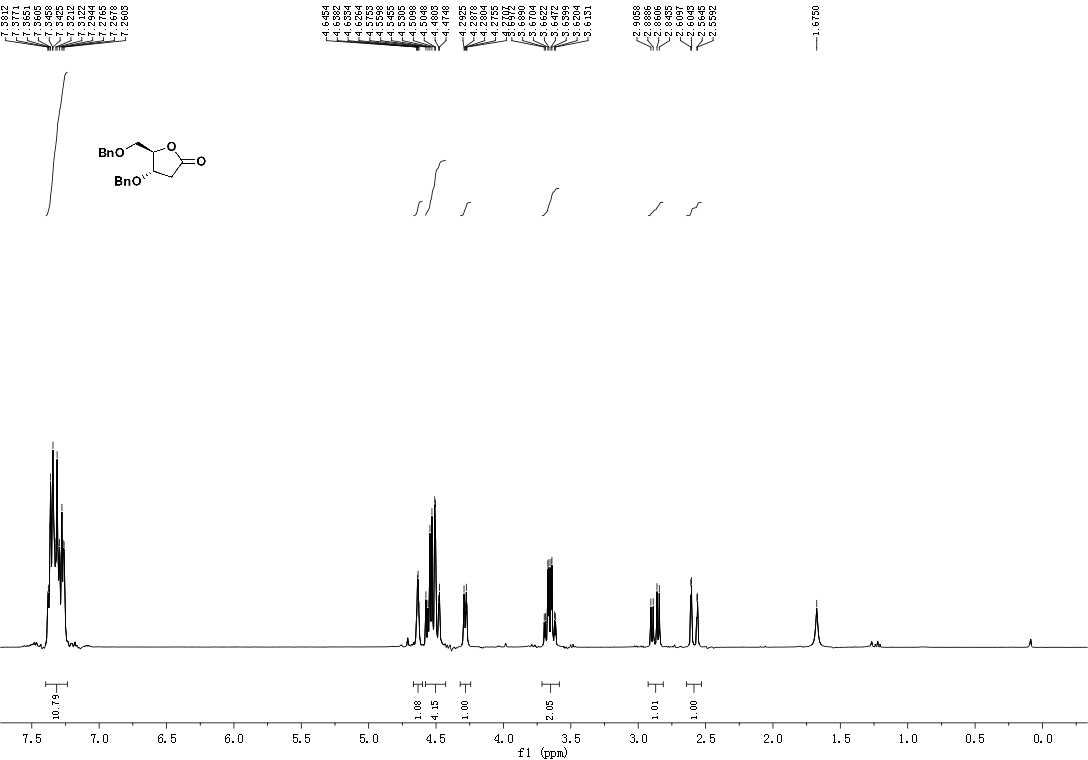


^1^H NMR spectra for **8c** (400 MHz, CDCl_3_)


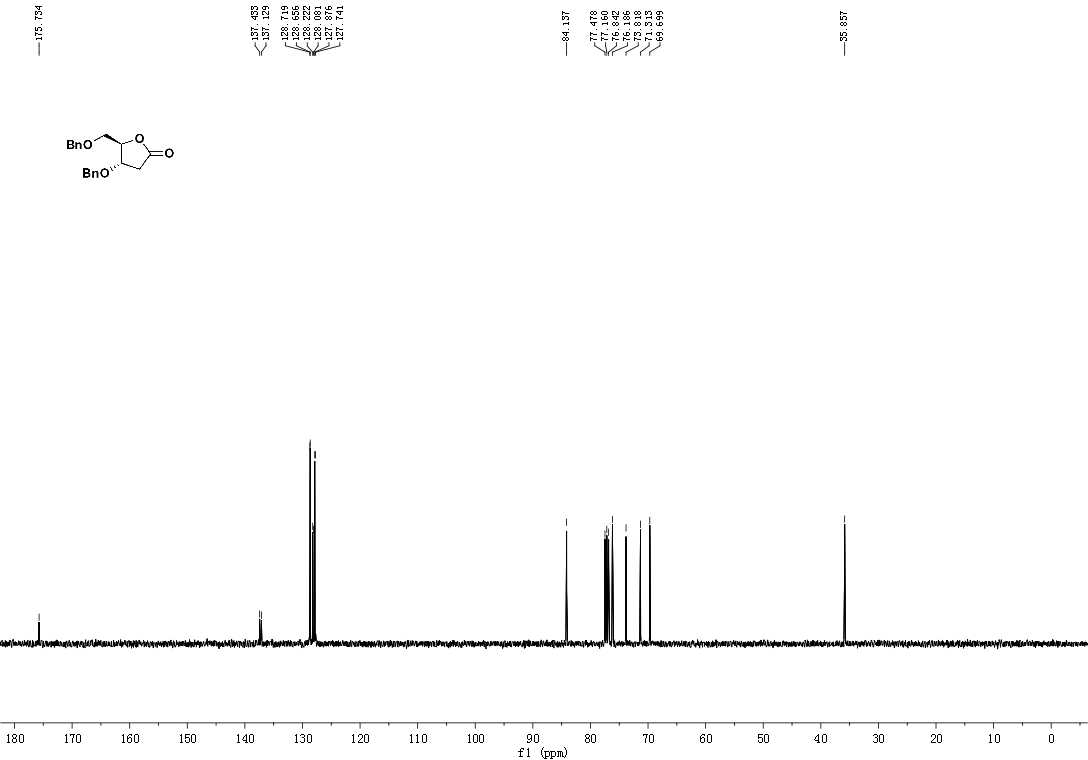


^13^C NMR spectra for **8c** (100 MHz, CDCl_3_)


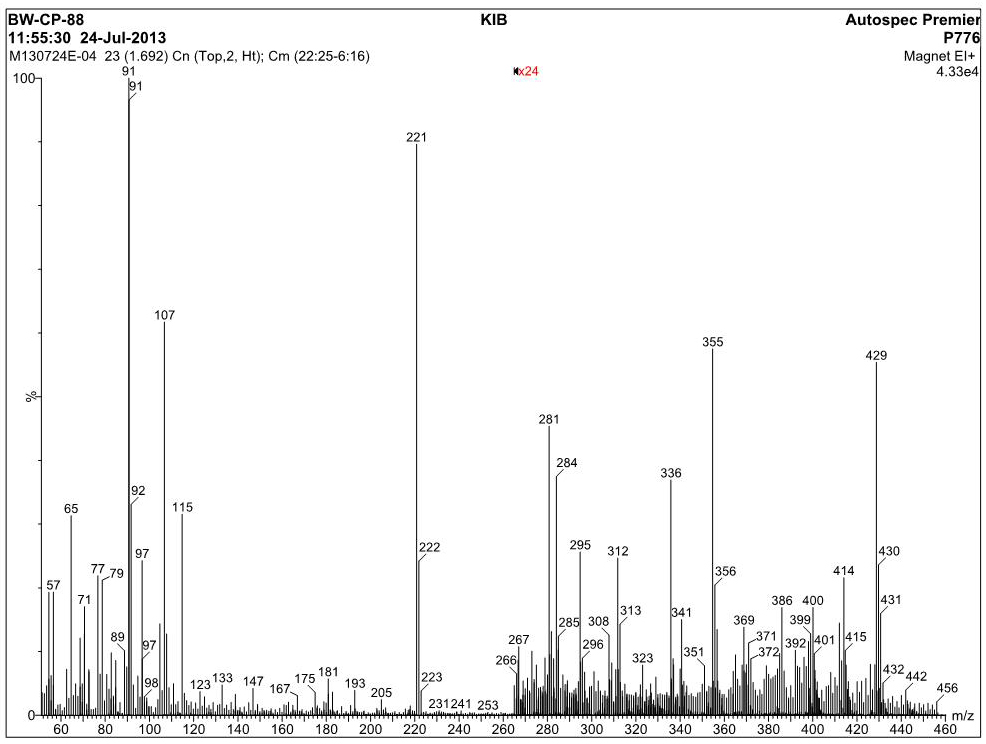


EI-MS spectra for **8c**


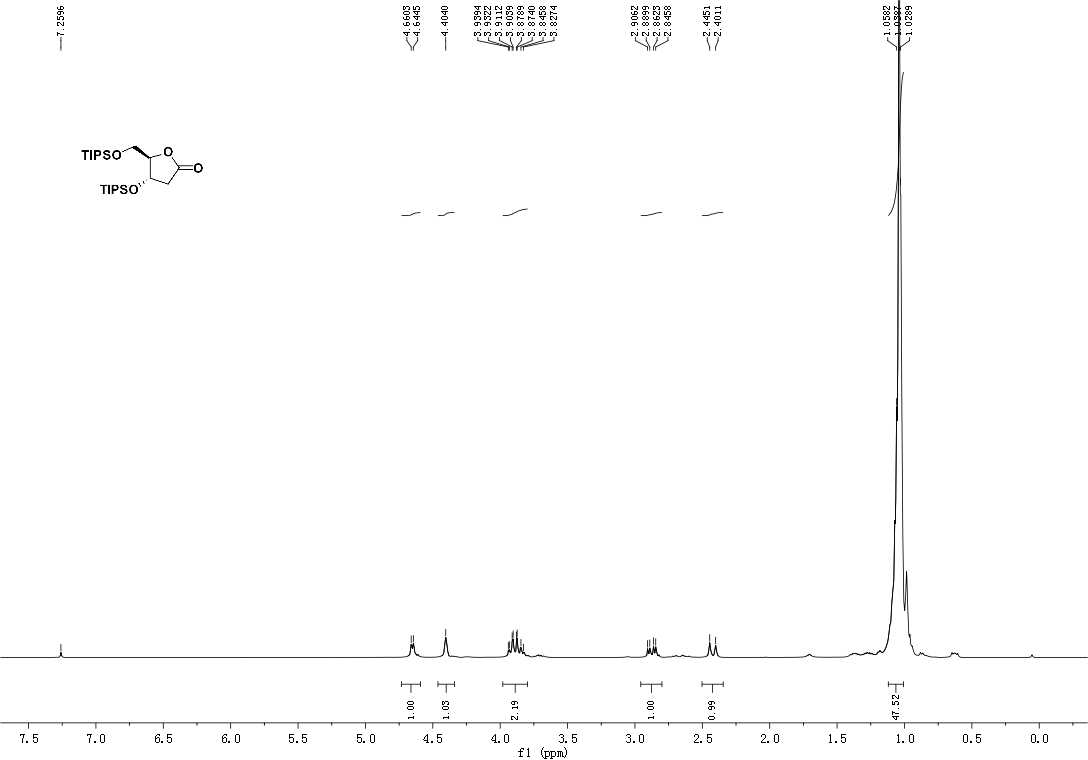


^1^H NMR spectra for **8d** (400 MHz, CDCl_3_)


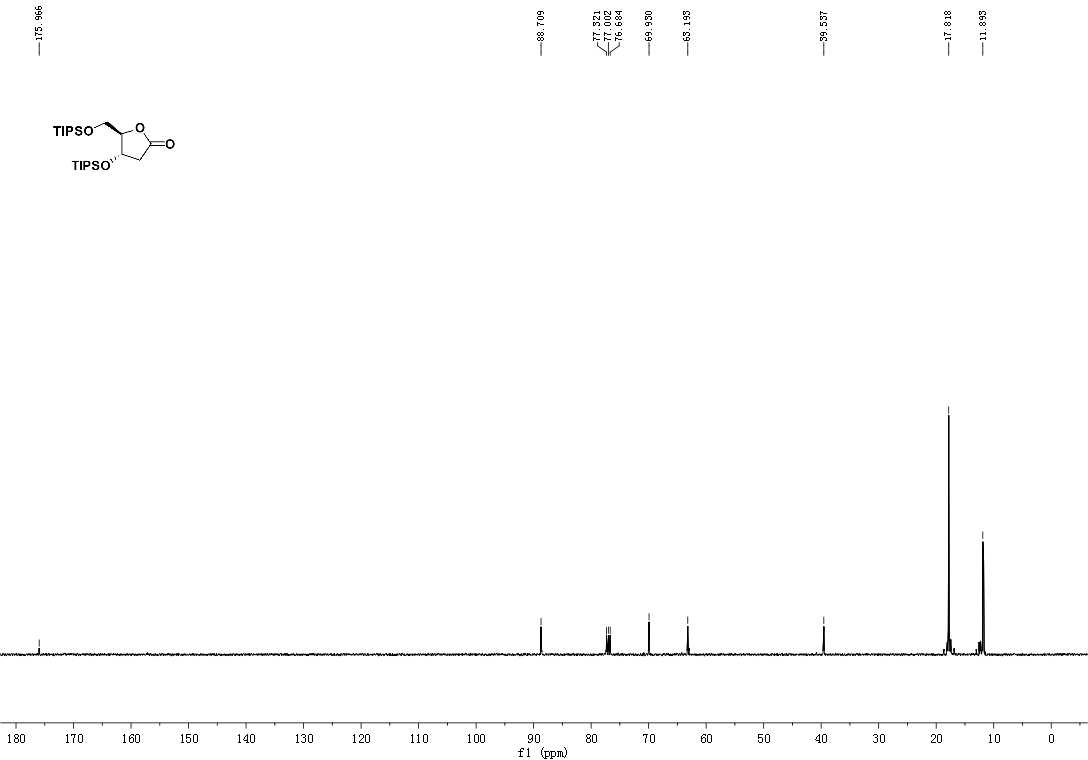


^13^C NMR spectra for **8d** (100 MHz, CDCl_3_)


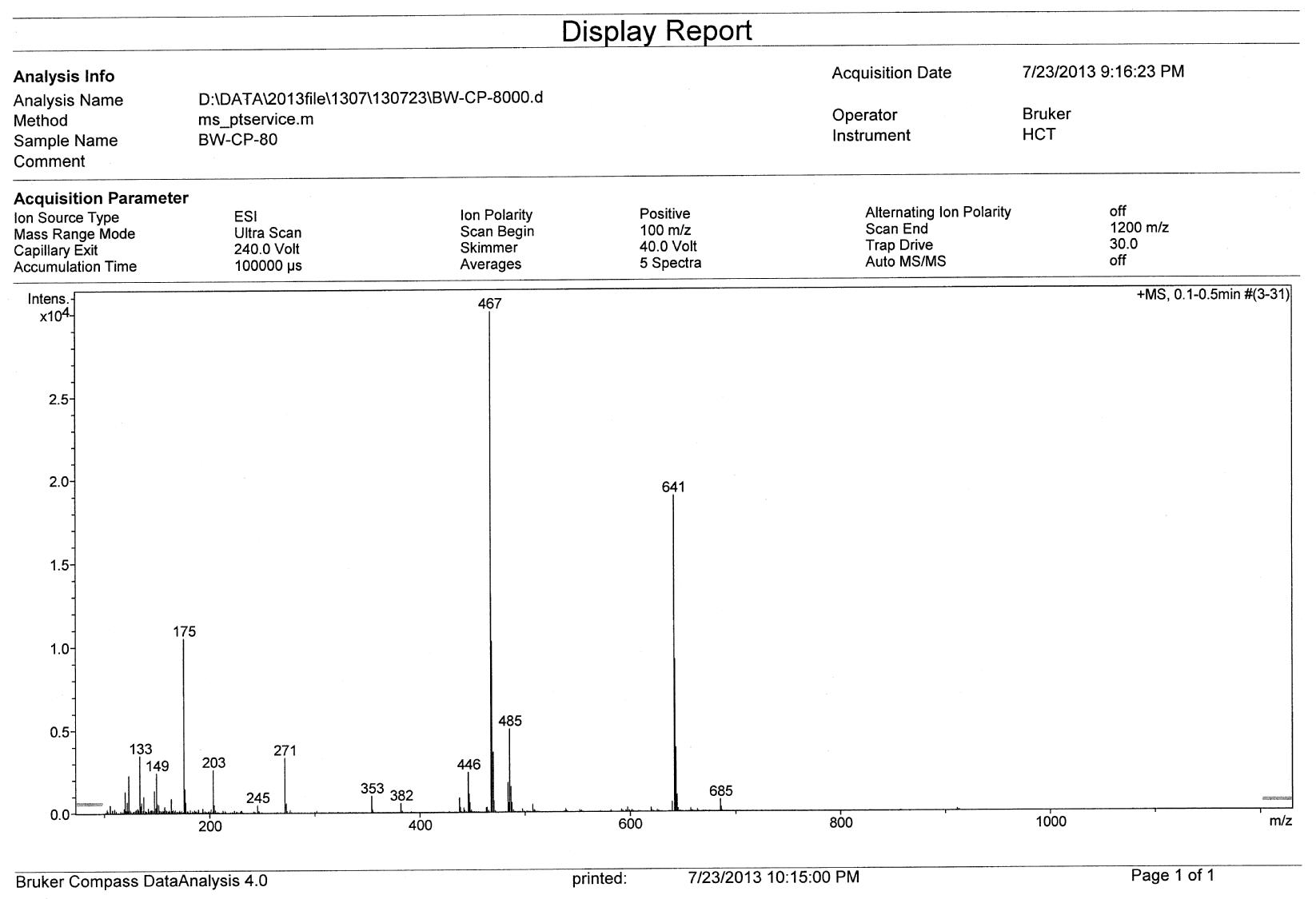


ESI-MS spectra for **8d**


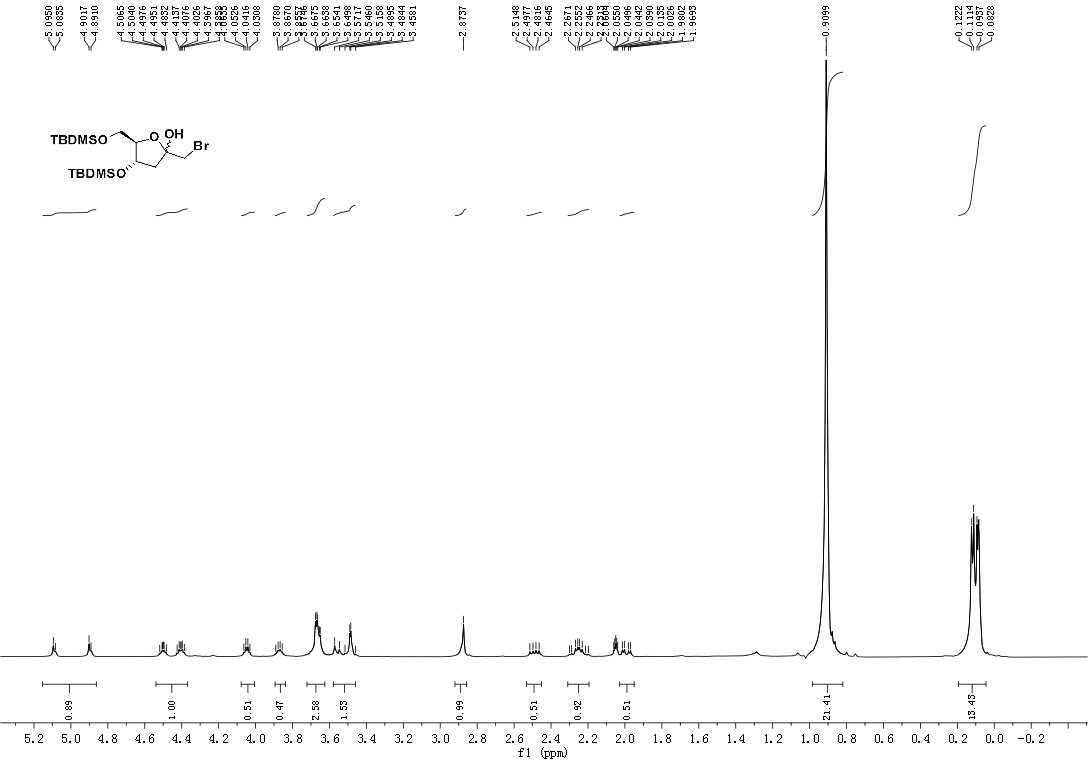


^1^H NMR spectra for **10a** (400 MHz, CD_3_COCD_3_)


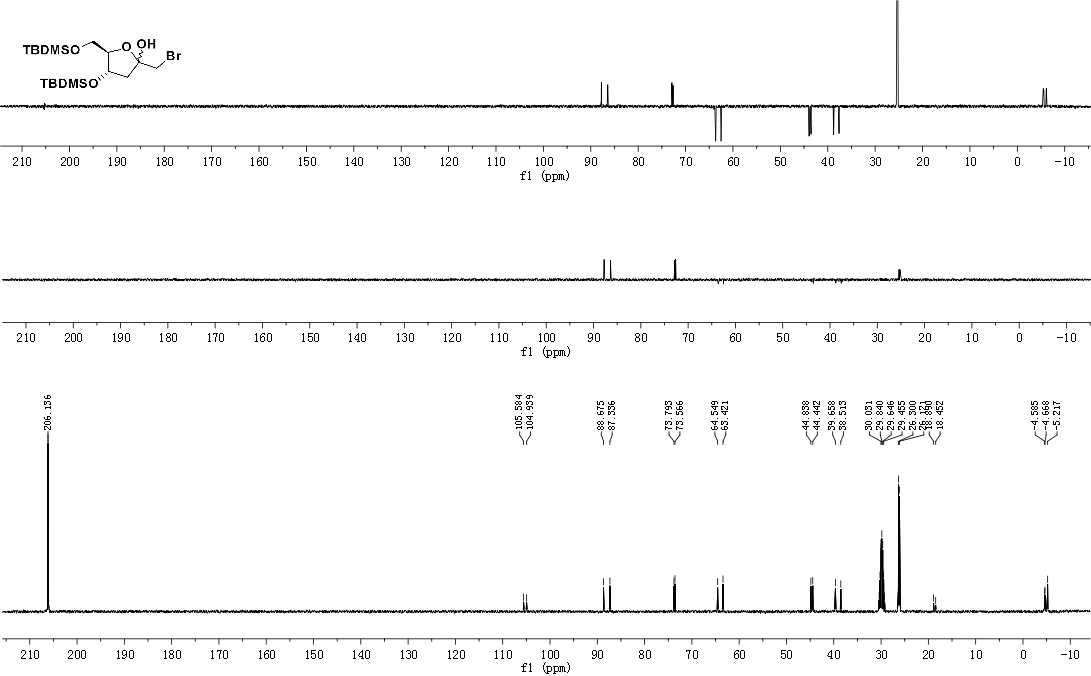


^13^C and DEPT NMR spectra for **10a** (100 MHz, CD_3_COCD_3_)


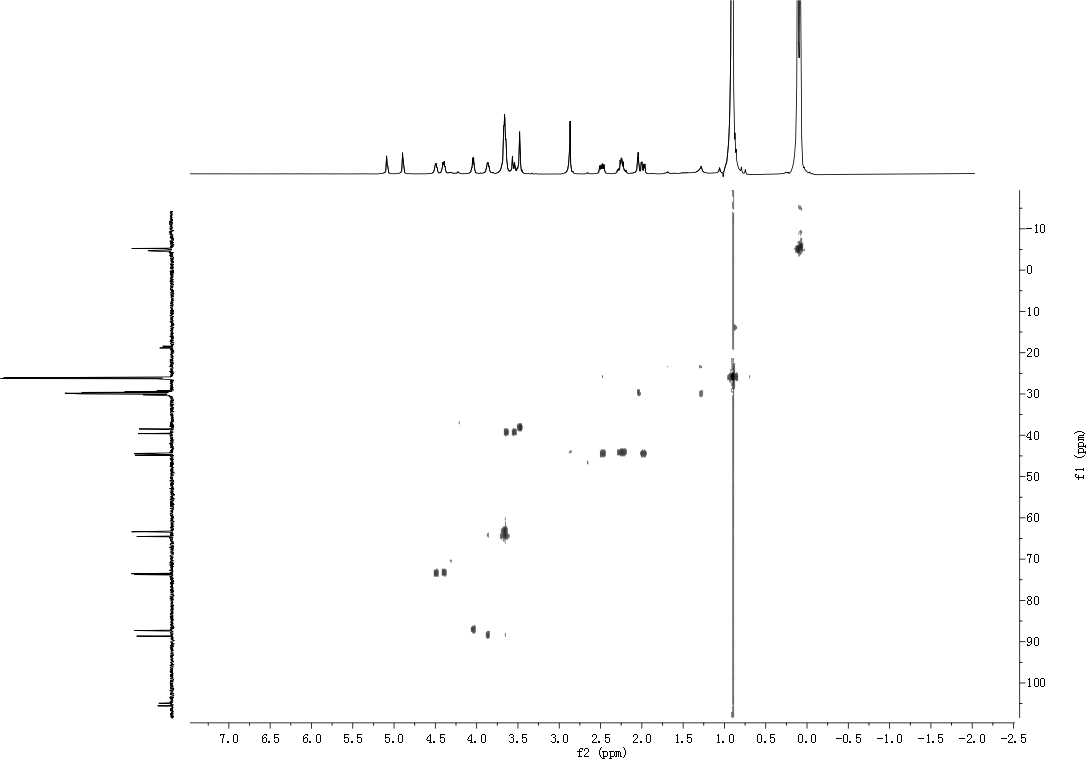


HSQC NMR spectra for **10a** (500 MHz, CD_3_COCD_3_)


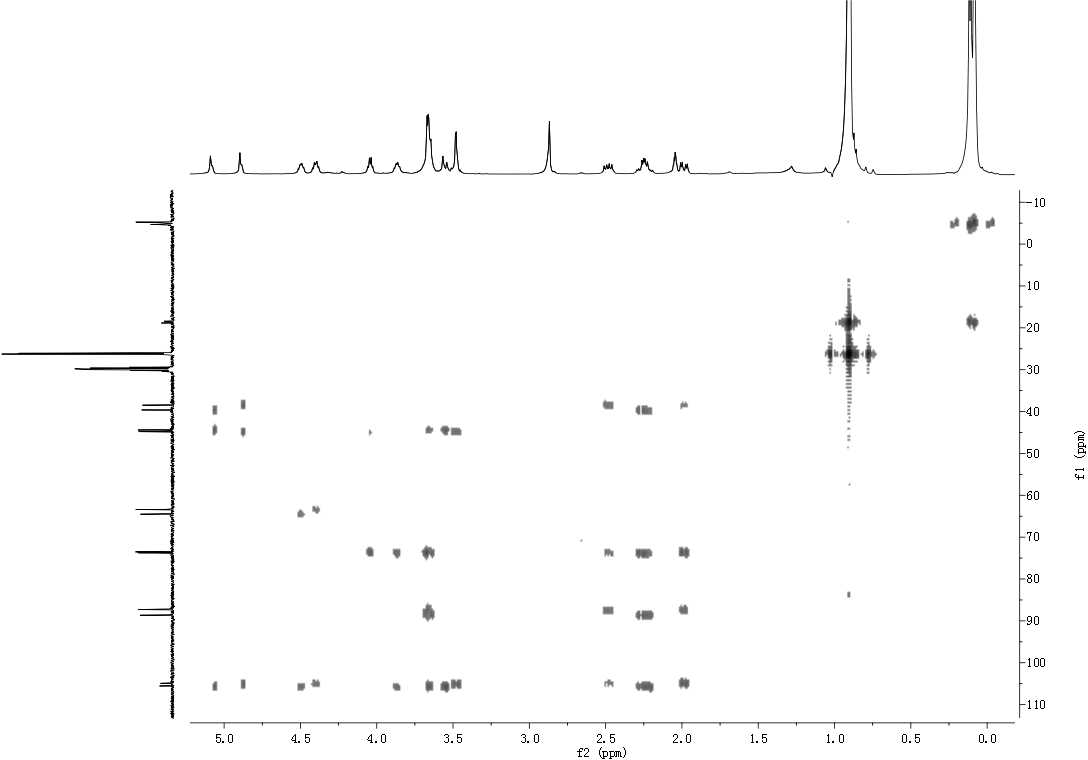


HMBC NMR spectra for **10a** (500 MHz, CD_3_COCD_3_)


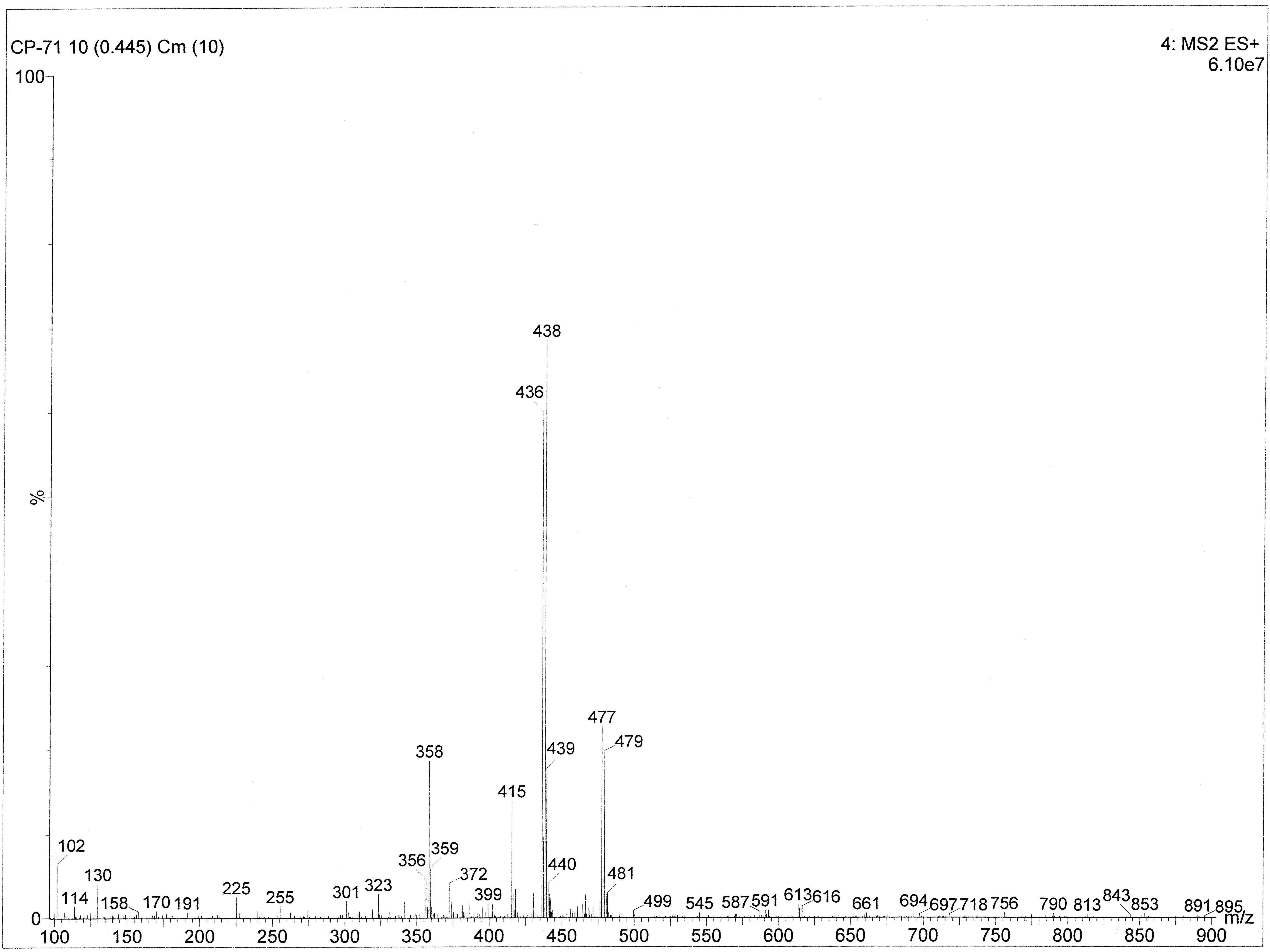


ESI-MS spectra for **10a**


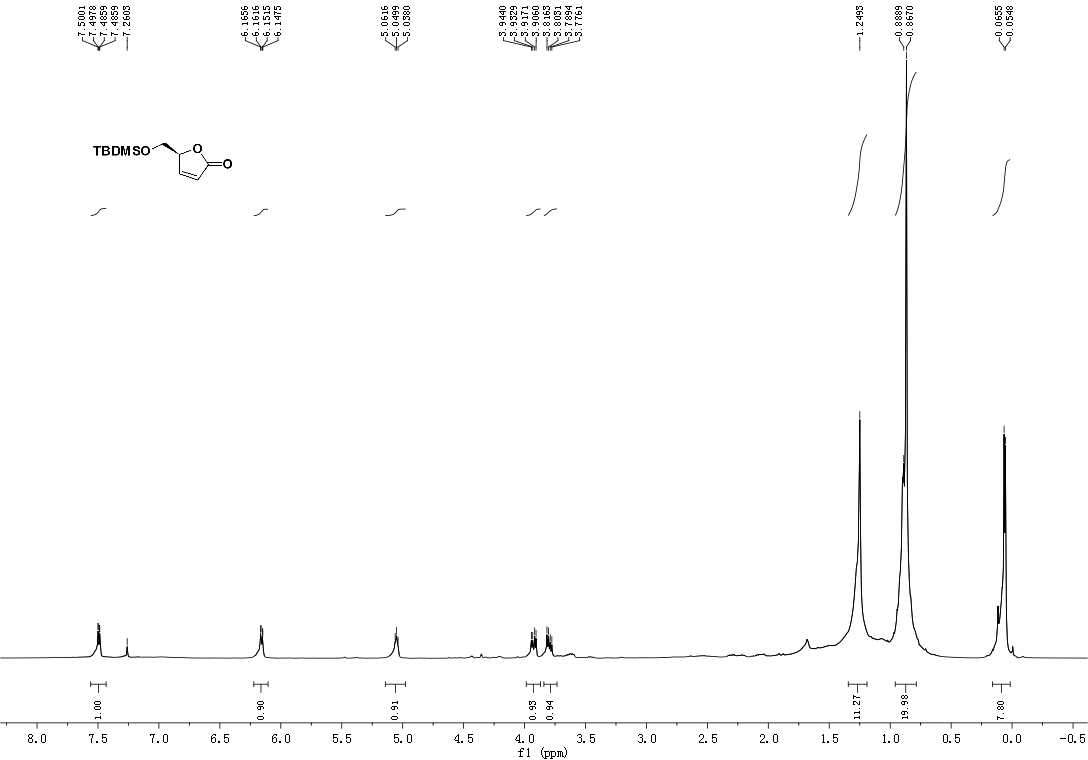


^1^H NMR spectra for **10a’** (400 MHz, CDCl_3_)


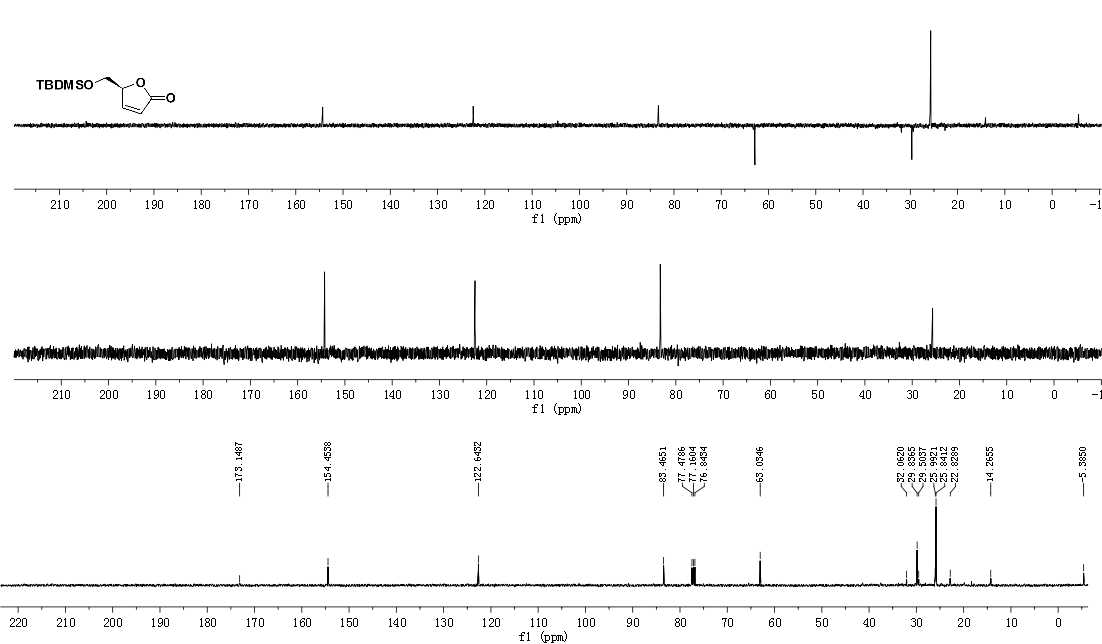


^13^C and DEPT NMR spectra for **10a’** (100 MHz, CDCl_3_)


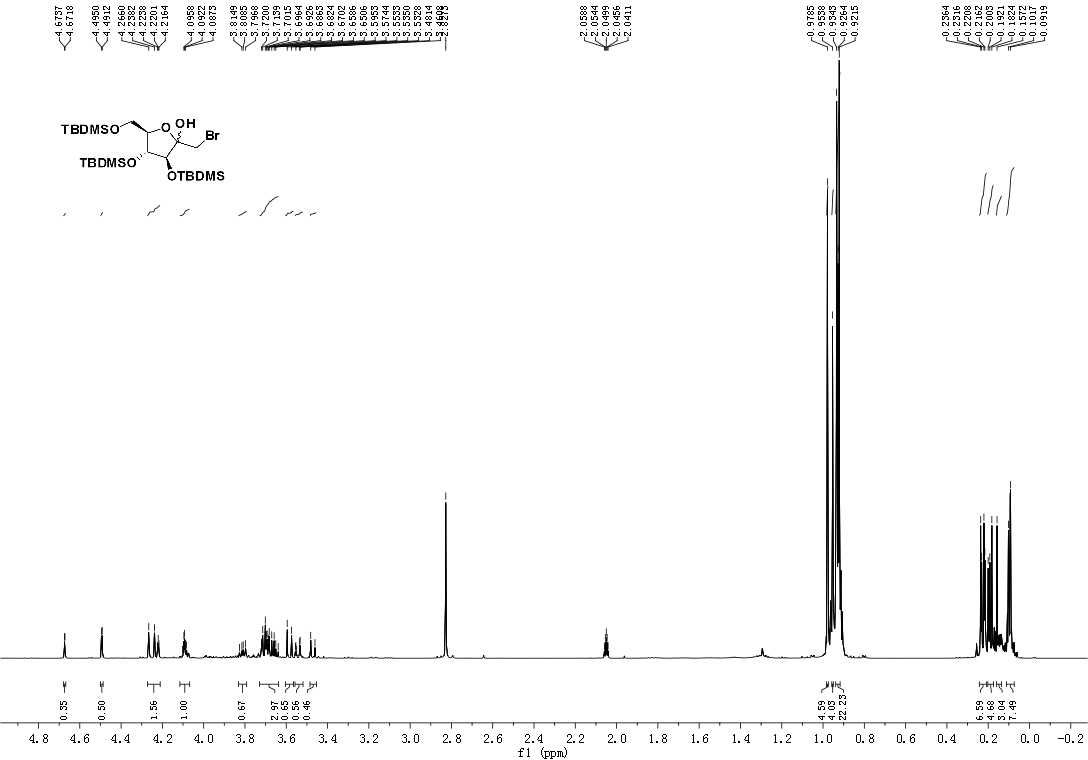


^1^H NMR spectra for **10b** (500 MHz, CD_3_COCD_3_)


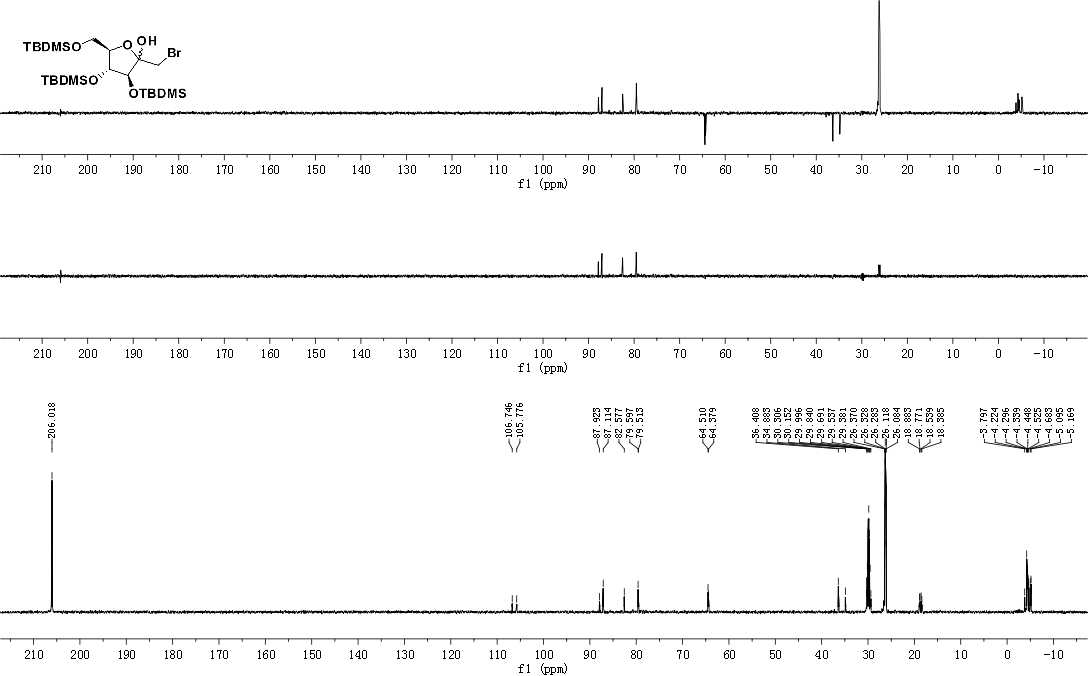


^13^C and DEPT NMR spectra for **10b** (100 MHz, CD_3_COCD_3_)


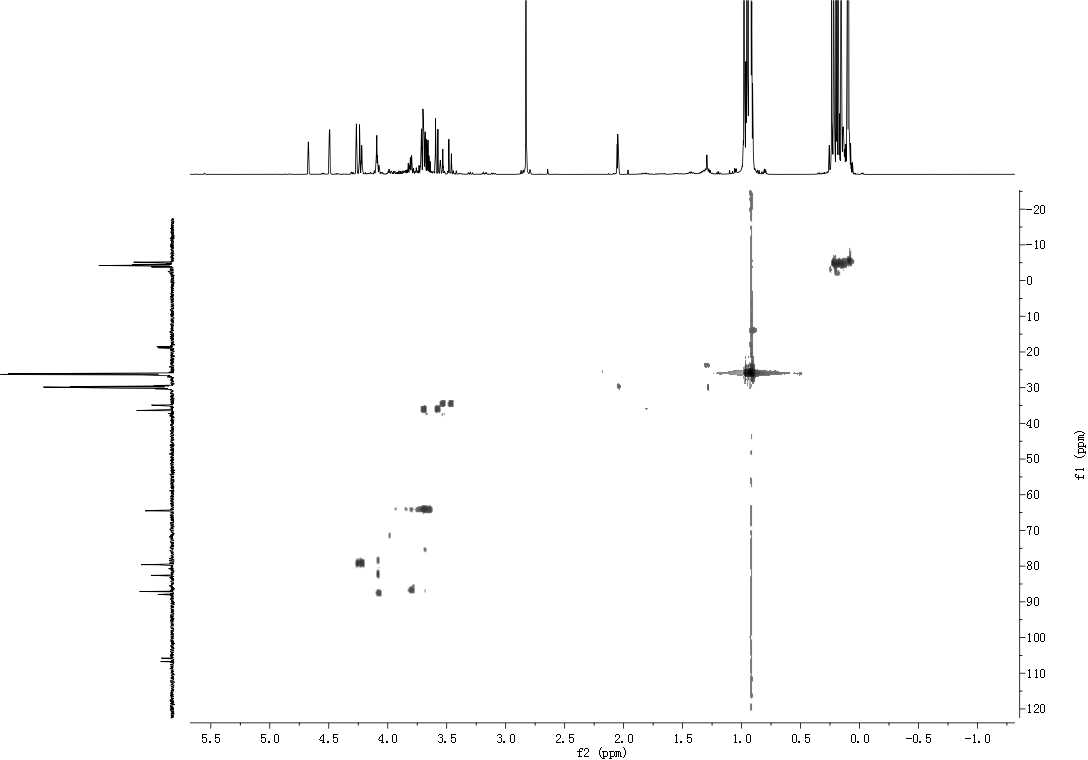


HSQC NMR spectra for **10b** (500 MHz, CD_3_COCD_3_)


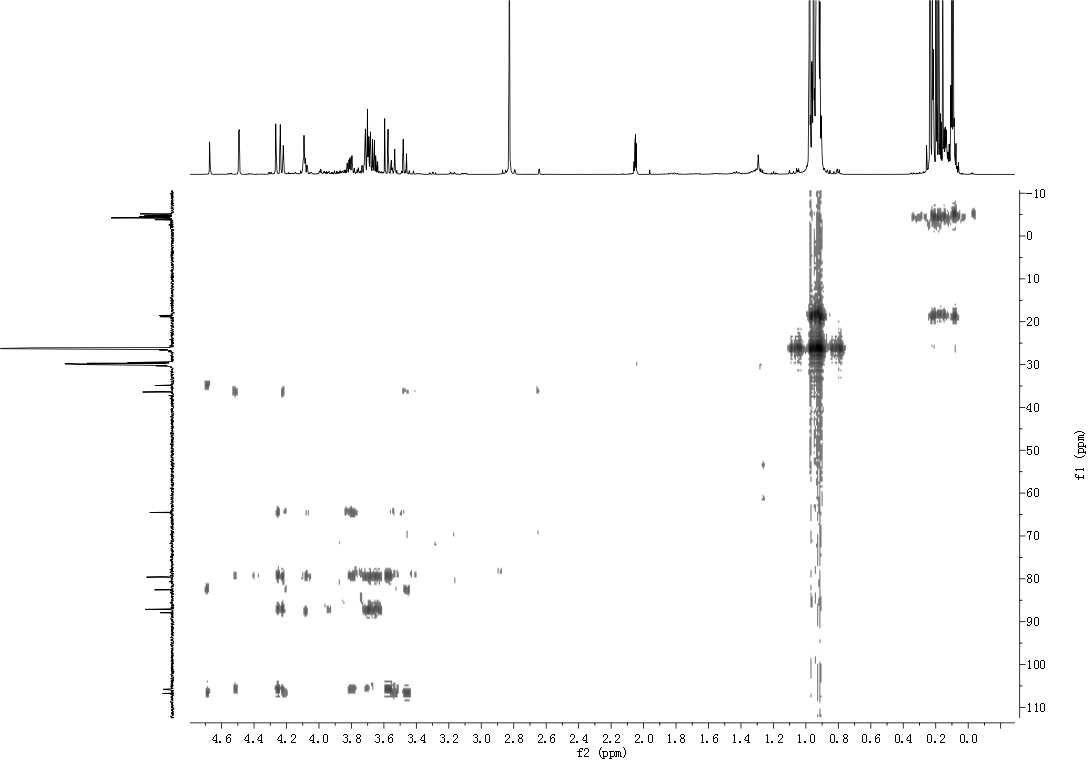


HMBC NMR spectra for **10b** (500 MHz, CD_3_COCD_3_)


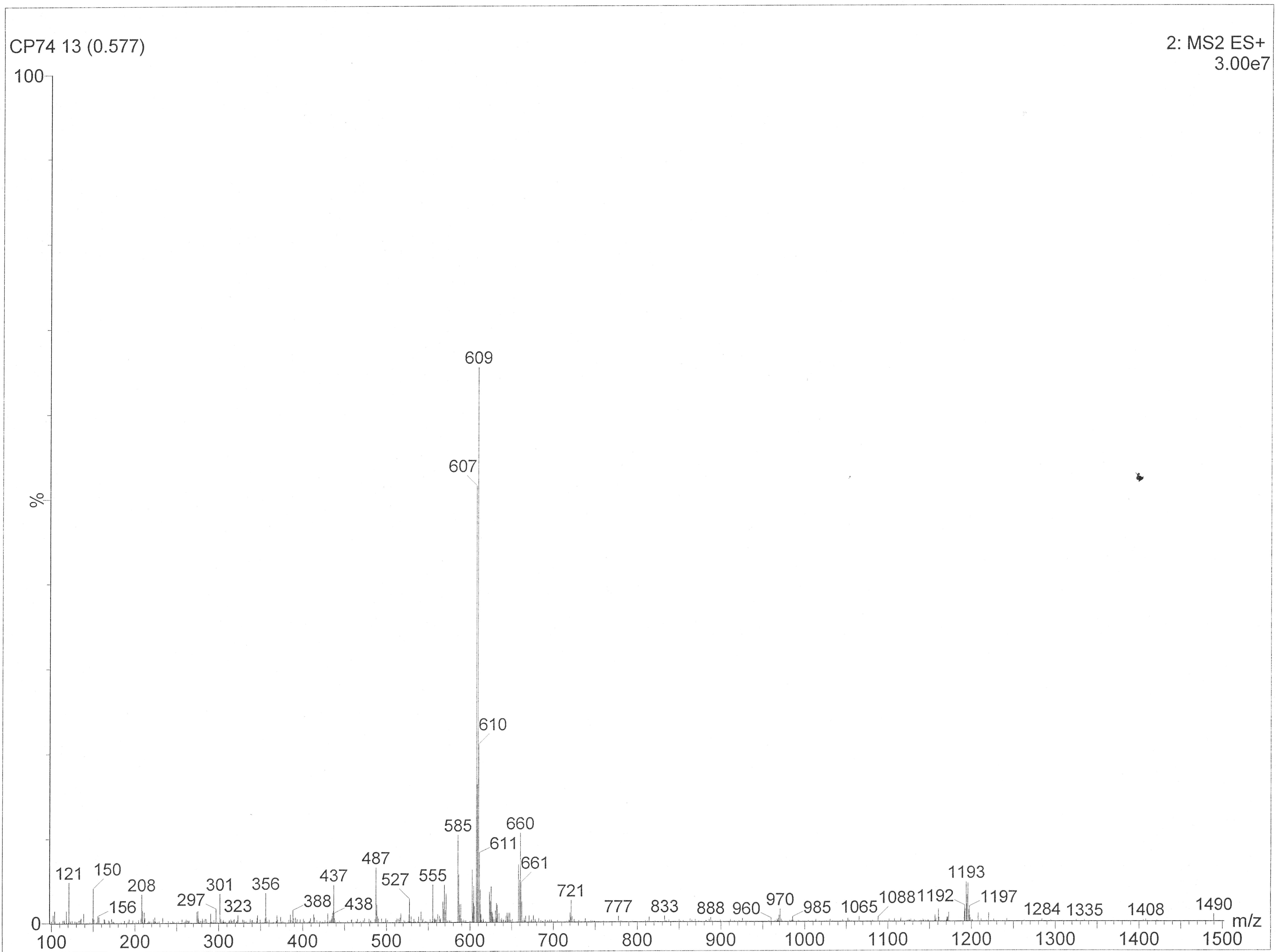


ESI-MS spectra for **10b**


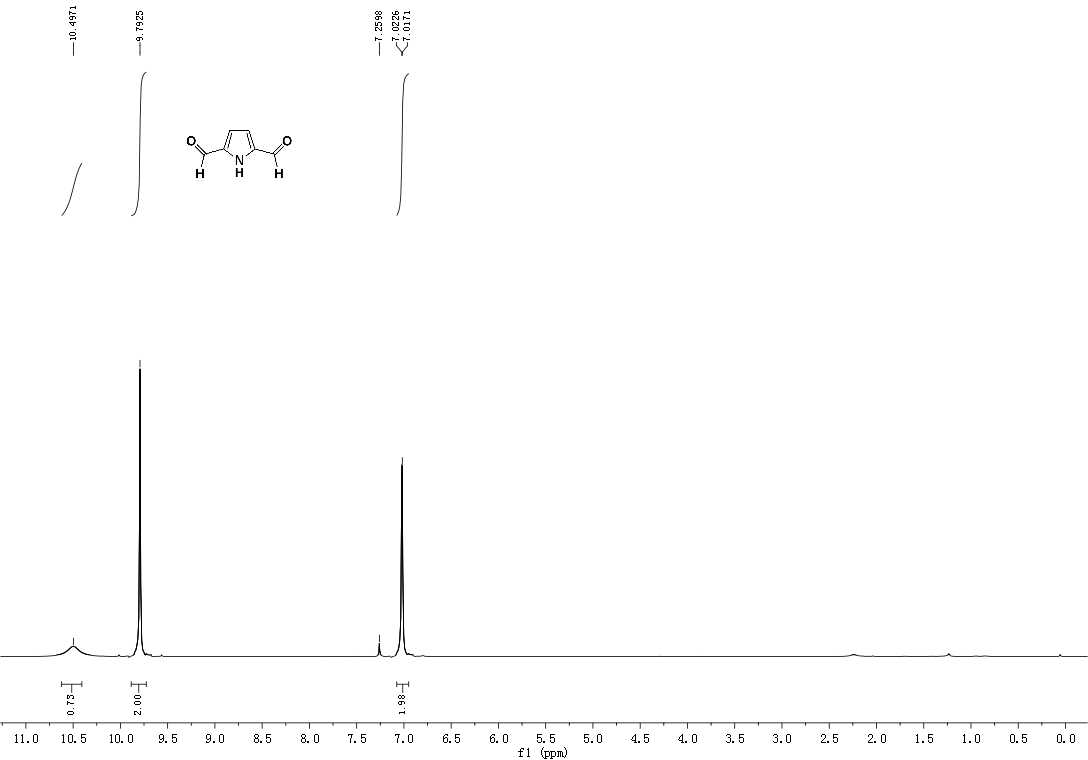


^1^H NMR spectra for **11** (400 MHz, CDCl_3_)


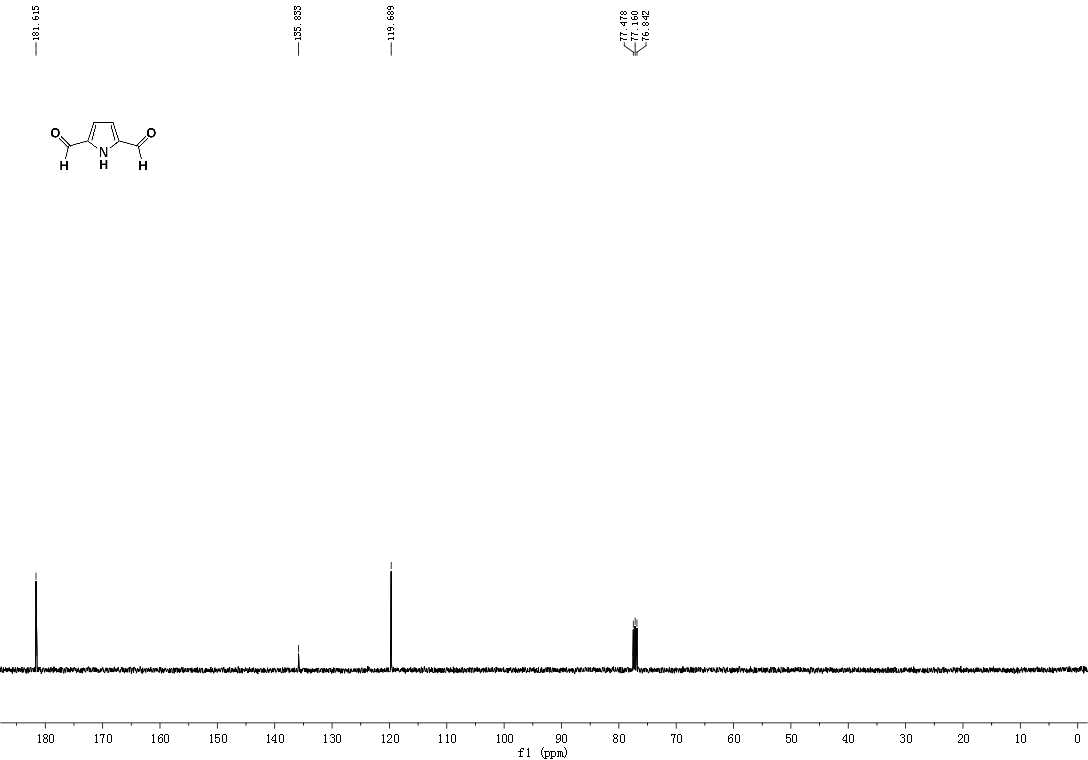


^13^C NMR spectra for **11** (100 MHz, CDCl_3_)


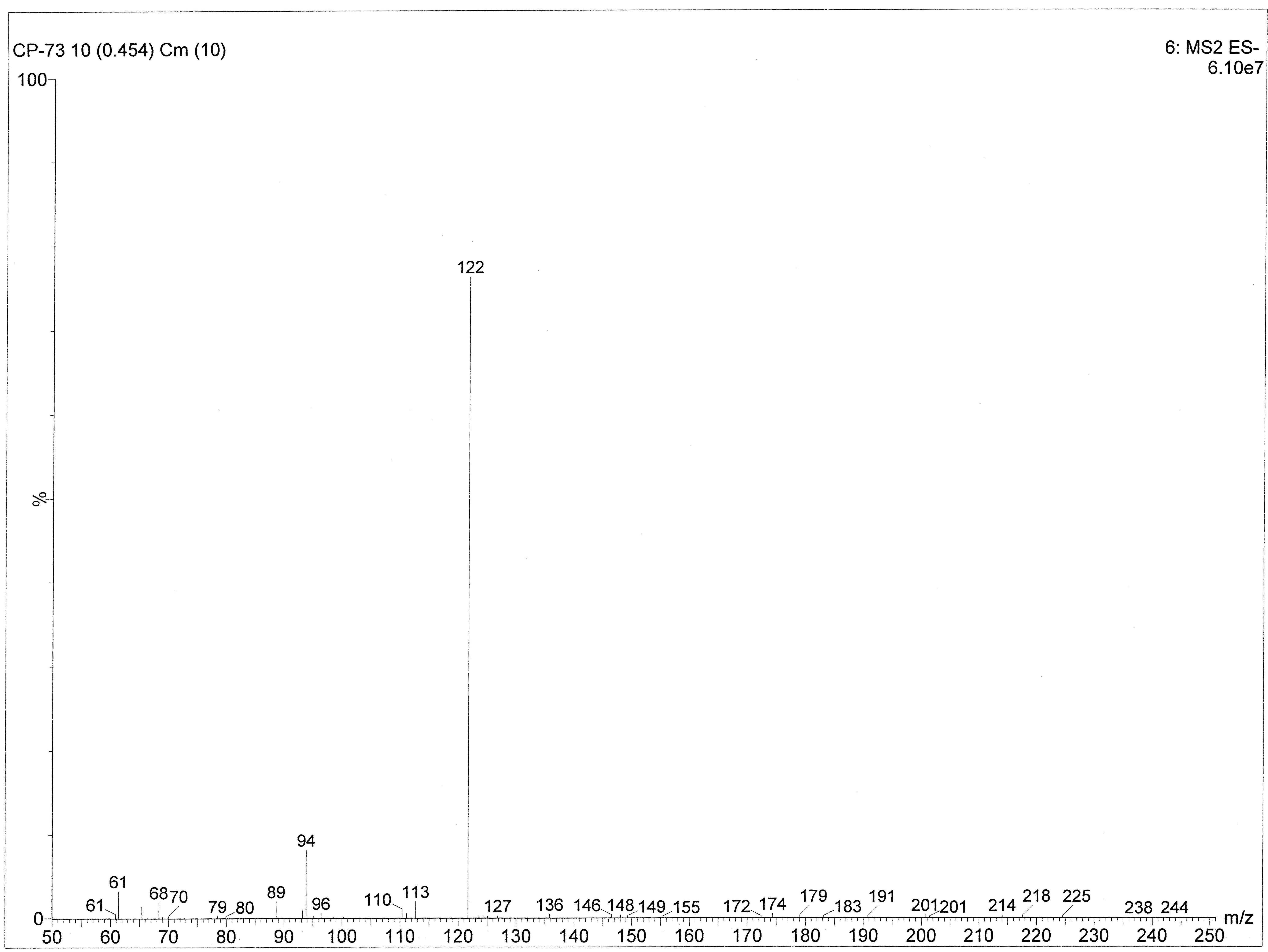


ESI-MS spectra for **11**


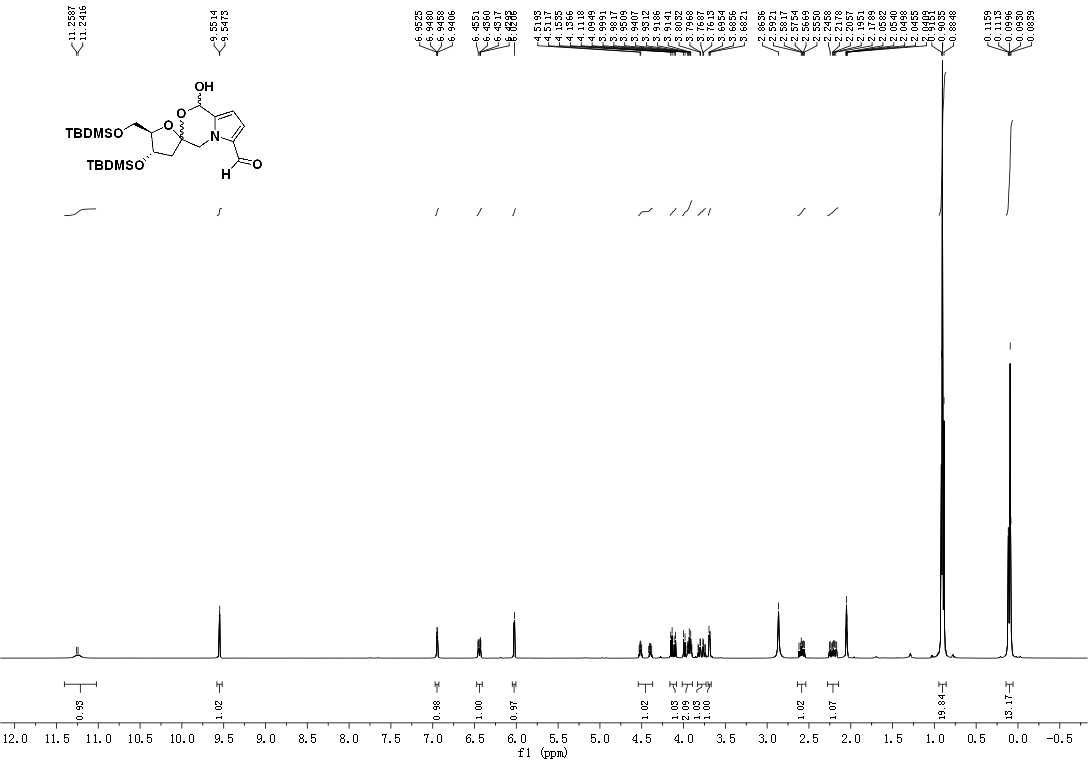


^1^H NMR spectra for **12a** (500 MHz, CD_3_COCD_3_)


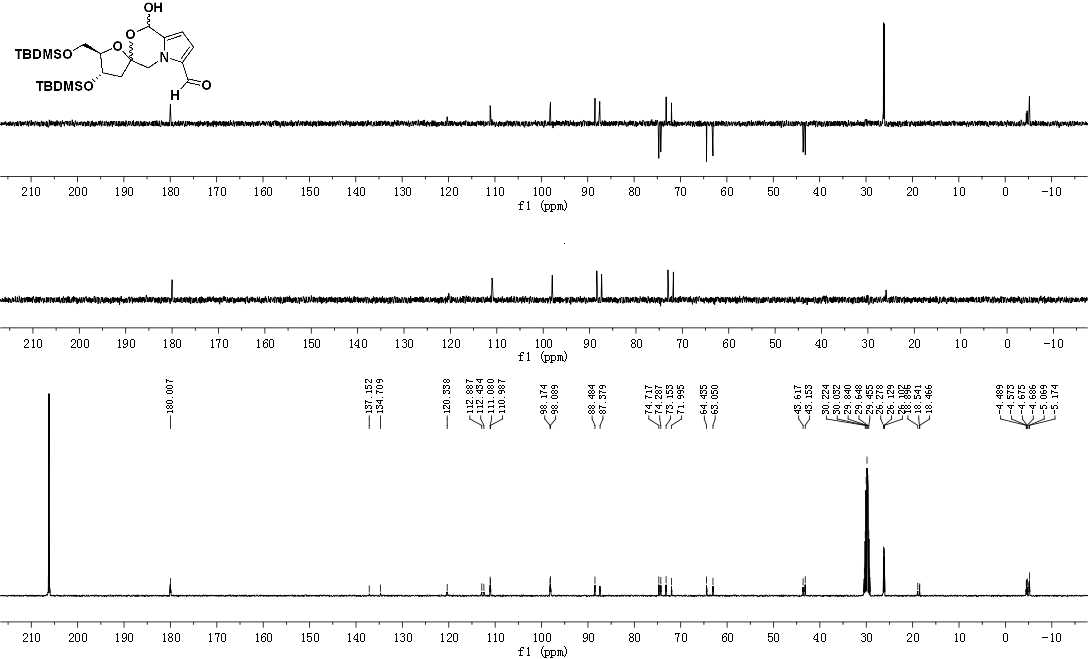


^13^C and DEPT NMR spectra for **12a** (100 MHz, CD_3_COCD_3_)


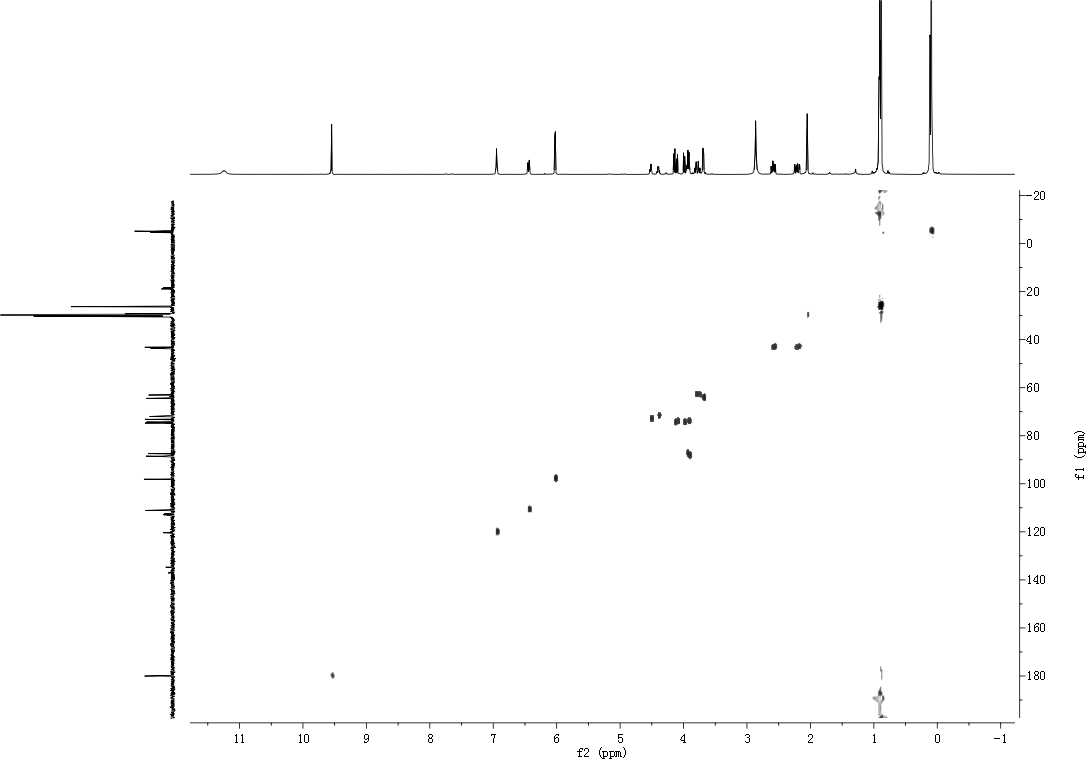


HSQC NMR spectra for **12a** (500 MHz, CD_3_COCD_3_)


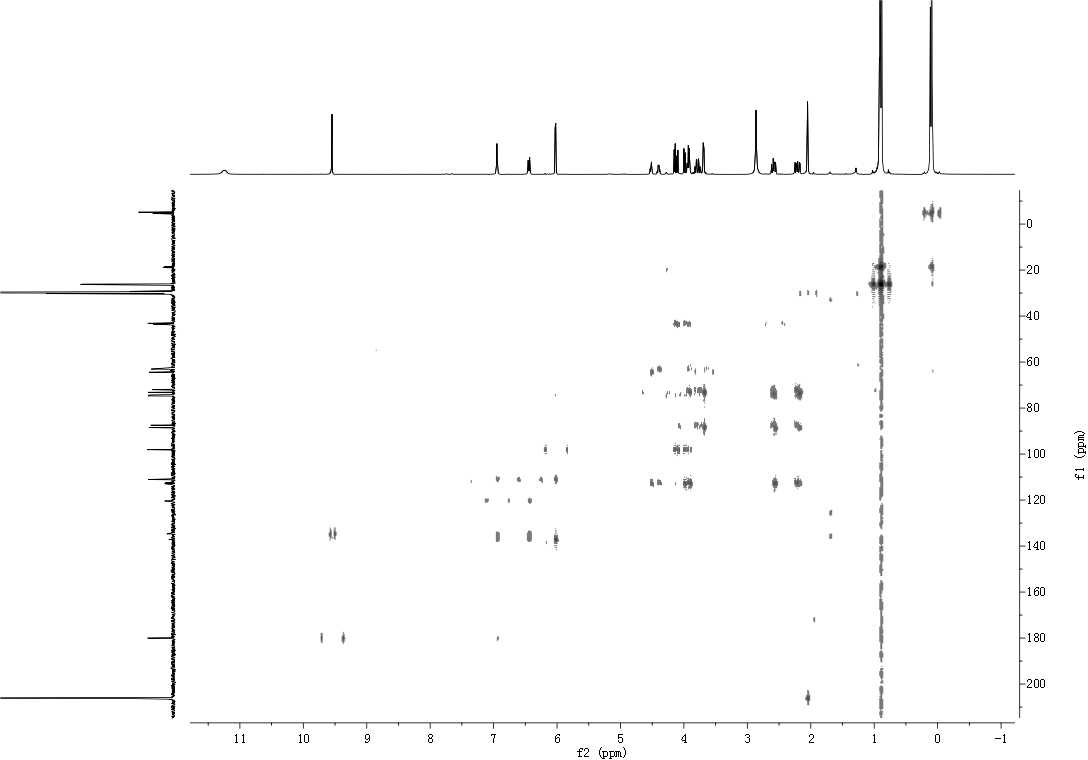


HMBC NMR spectra for **12a** (500 MHz, CD_3_COCD_3_)


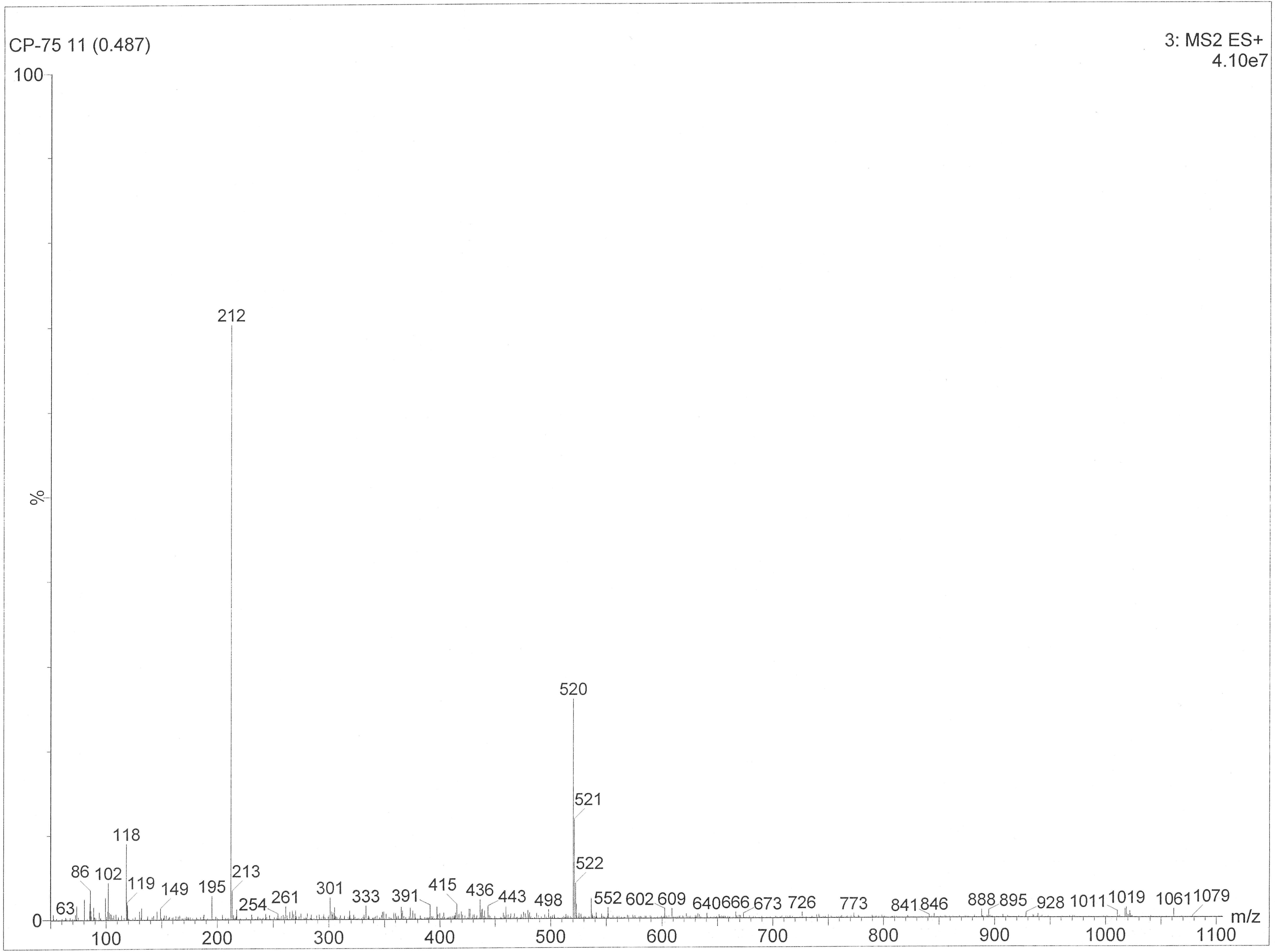


ESI-MS spectra for **12a**


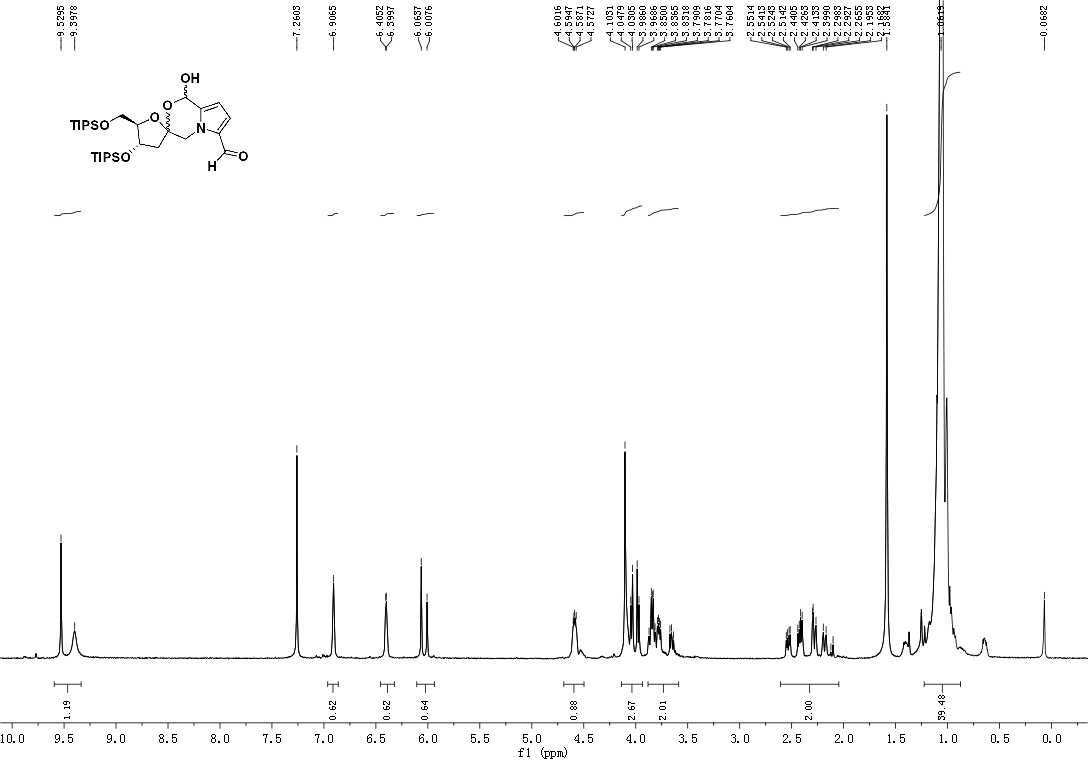


^1^H NMR spectra for **12b** (500 MHz, CDCl_3_)


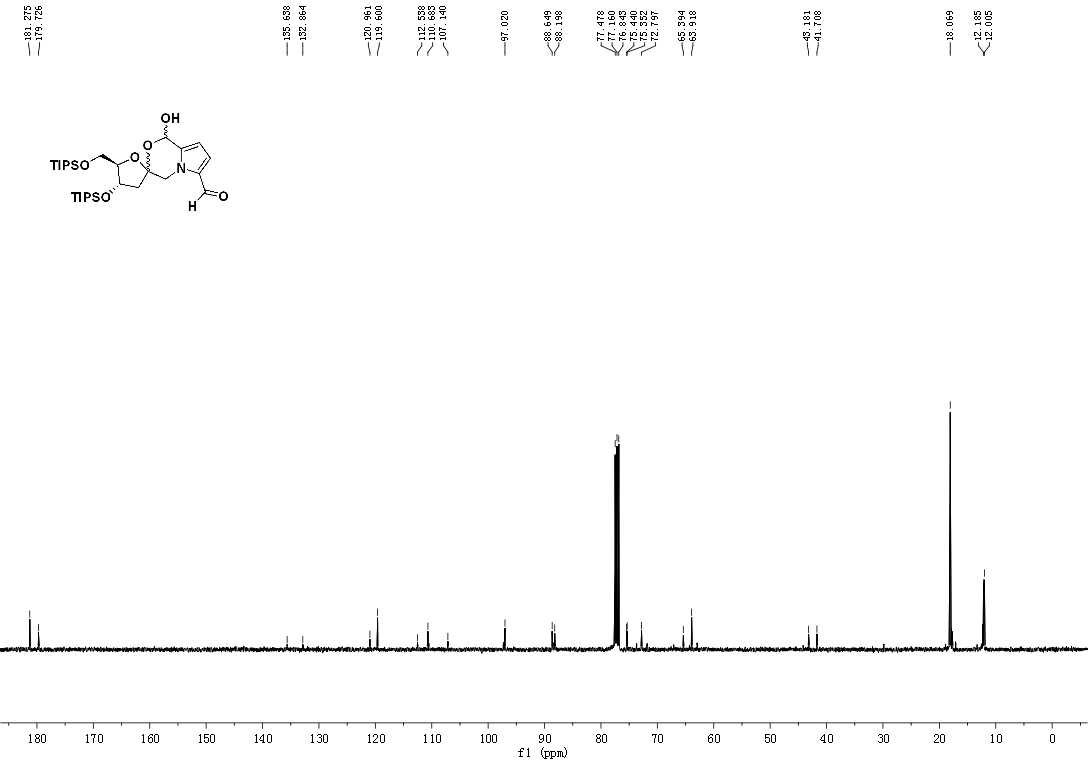


^13^C NMR spectra for **12b** (100 MHz, CDCl_3_)


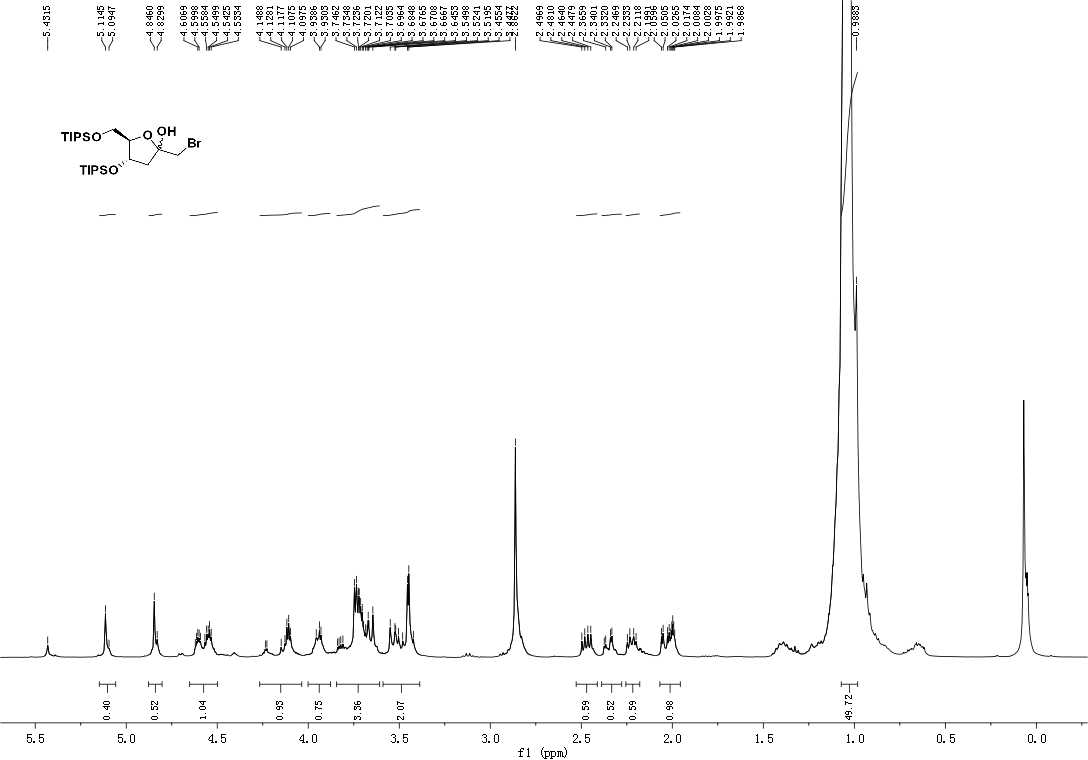


^1^H NMR spectra for **14a** (400 MHz, CD_3_COCD_3_)


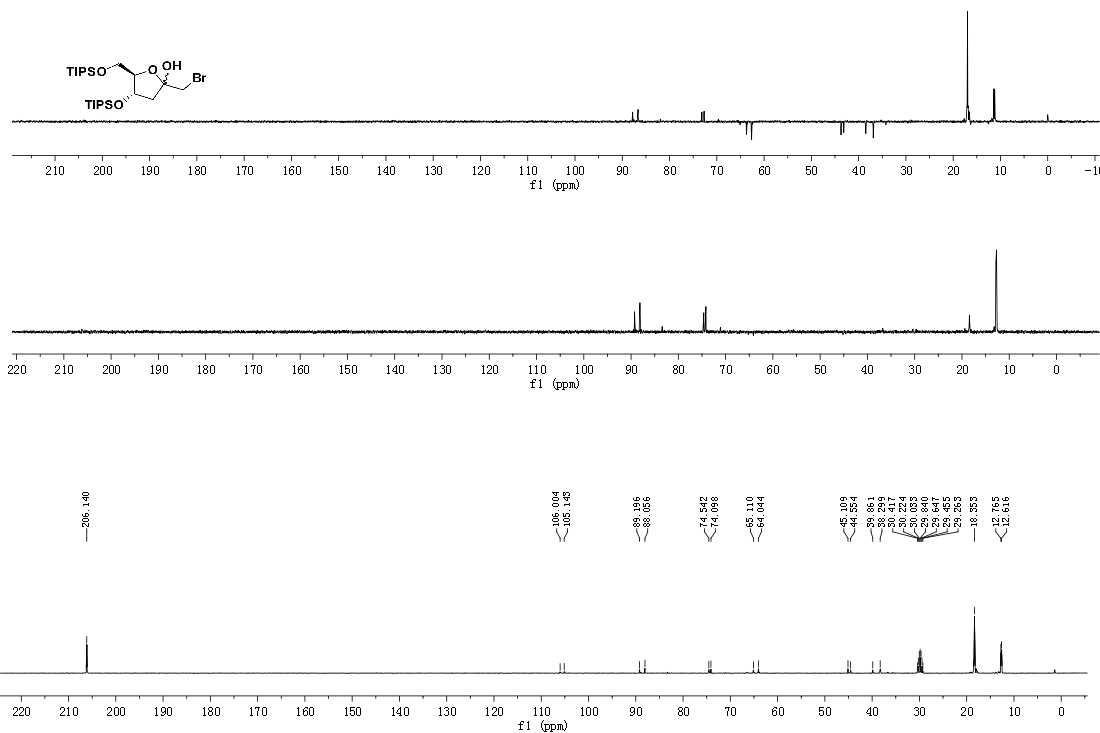


^13^C and DEPT NMR spectra for **14a** (100 MHz, CD_3_COCD_3_)


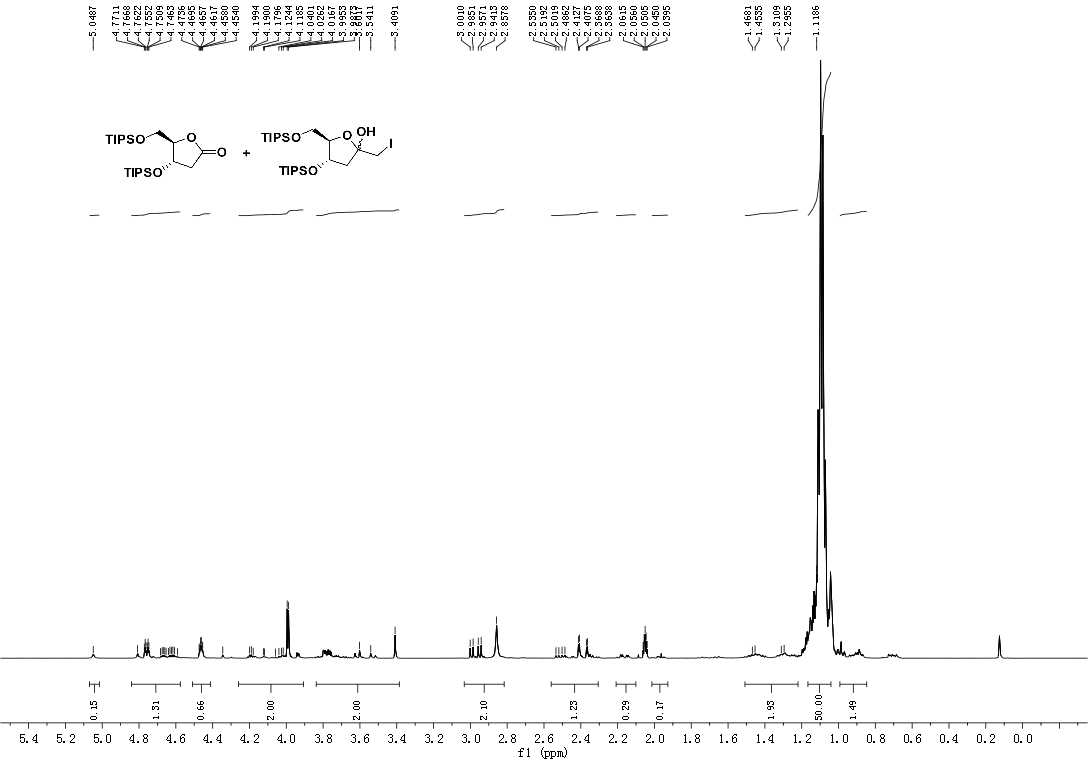


^1^H NMR spectra for crude **14b** (CH_2_I_2_/*n*-BuLi = 1.2/1) (400 MHz, CD_3_COCD_3_)


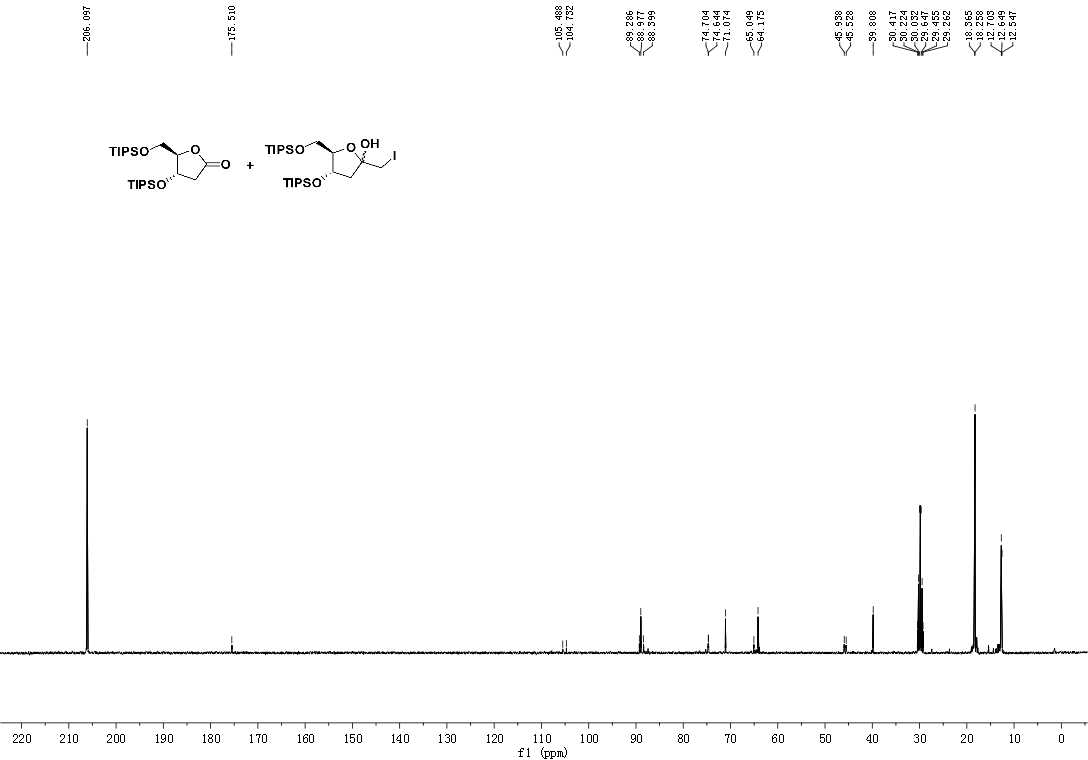


^13^C NMR spectra for crude **14b** (CH_2_I_2_/*n*-BuLi = 1.2/1) (100 MHz, CD_3_COCD_3_)


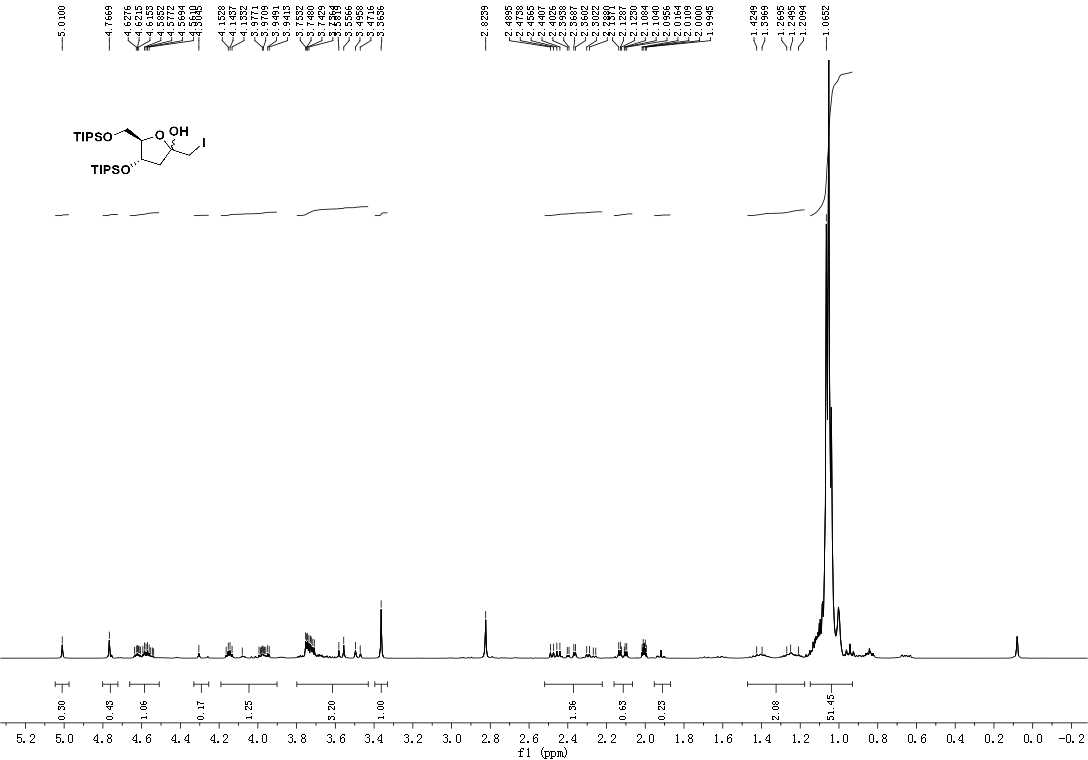


^1^H NMR spectra for **14b** (400 MHz, CD_3_COCD_3_)


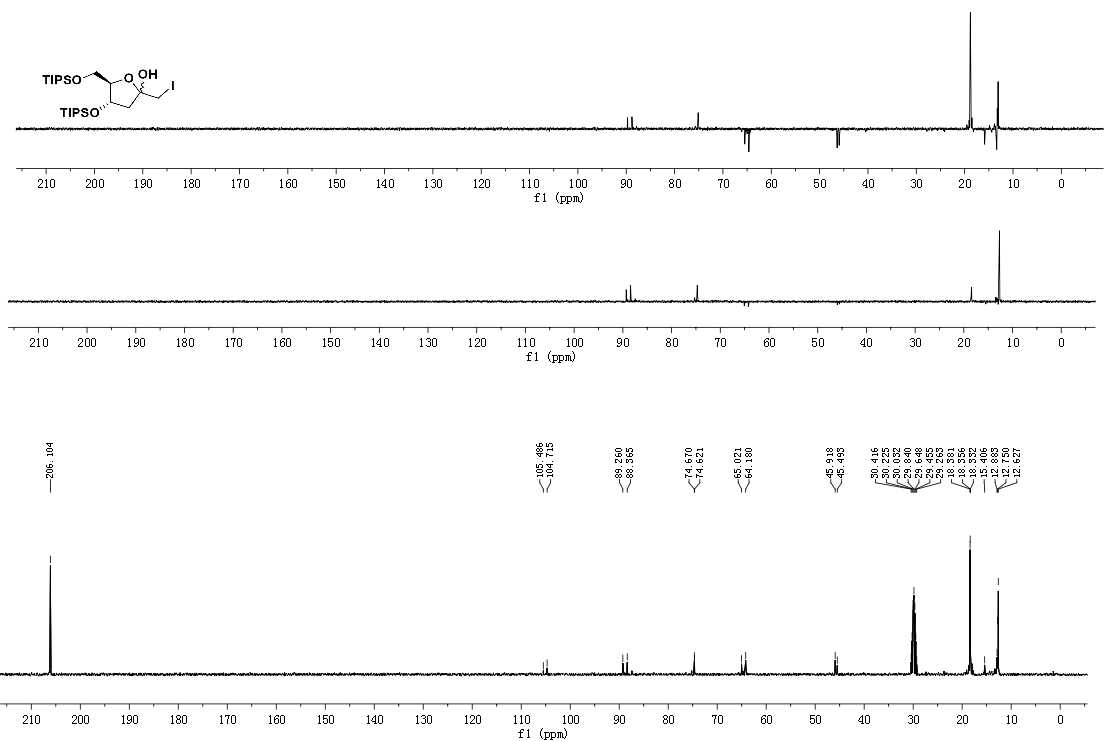


^13^C and DEPT NMR spectra for **14b** (100 MHz, CD_3_COCD_3_)


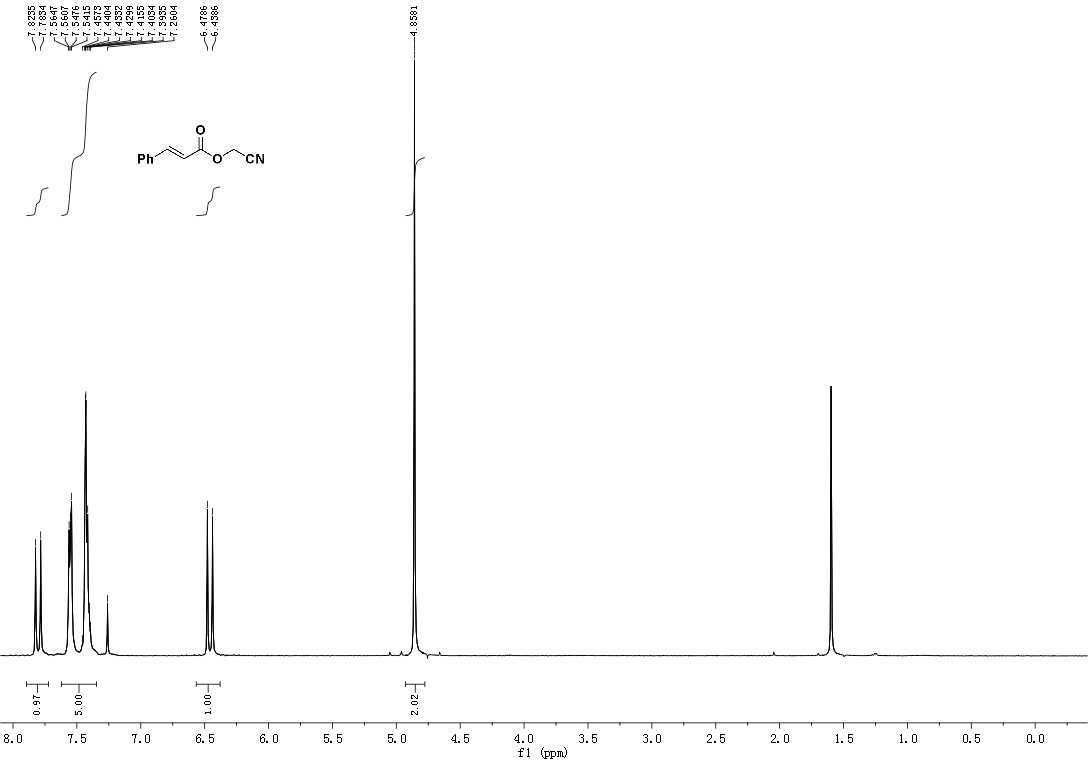


^1^H NMR spectra for **18** (400 MHz, CDCl_3_)


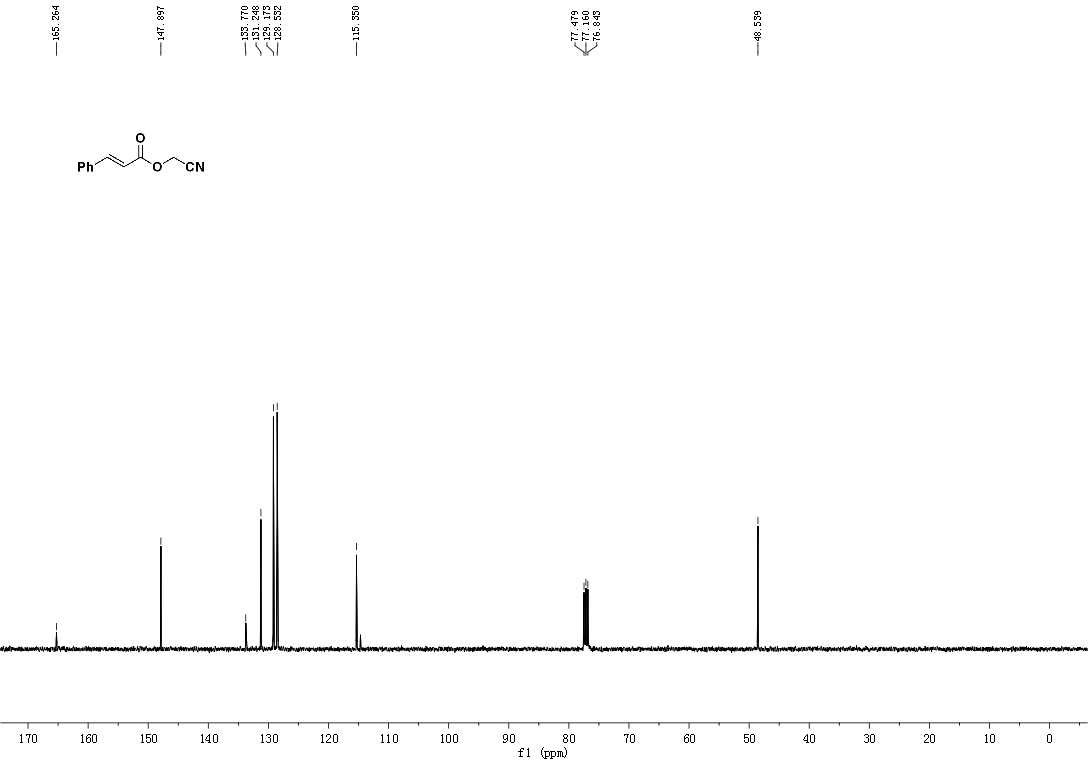


^13^C NMR spectra for **18** (100 MHz, CDCl_3_)


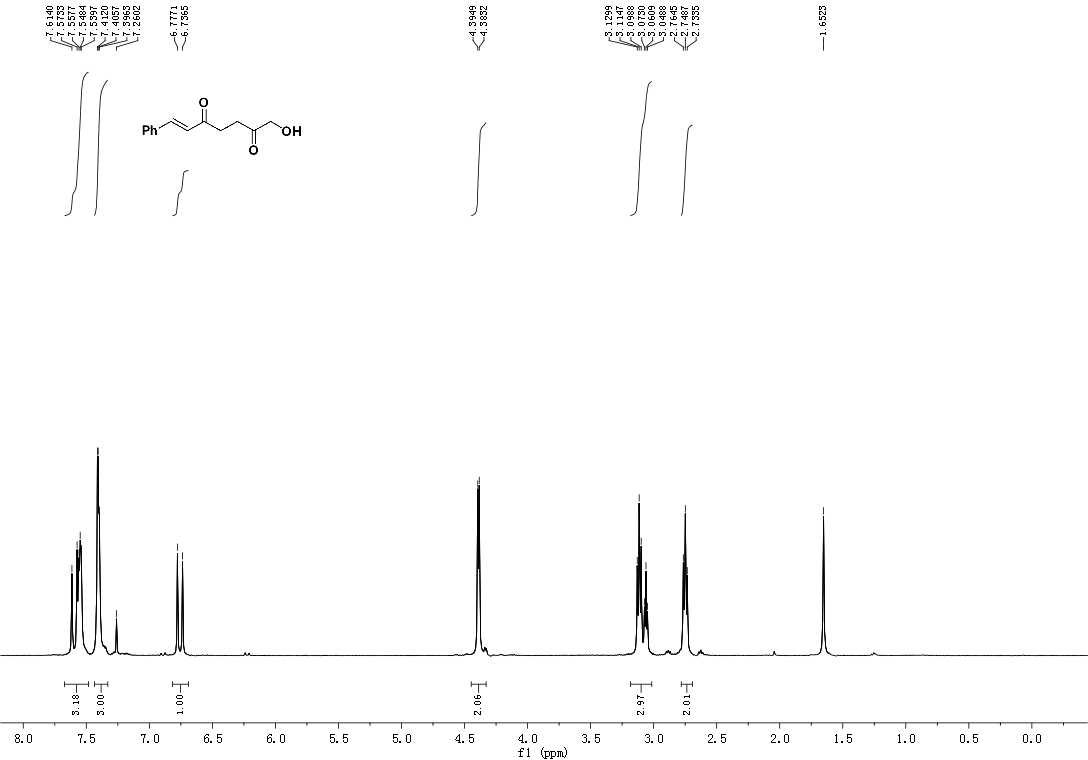


^1^H NMR spectra for **19** (400 MHz, CDCl_3_)


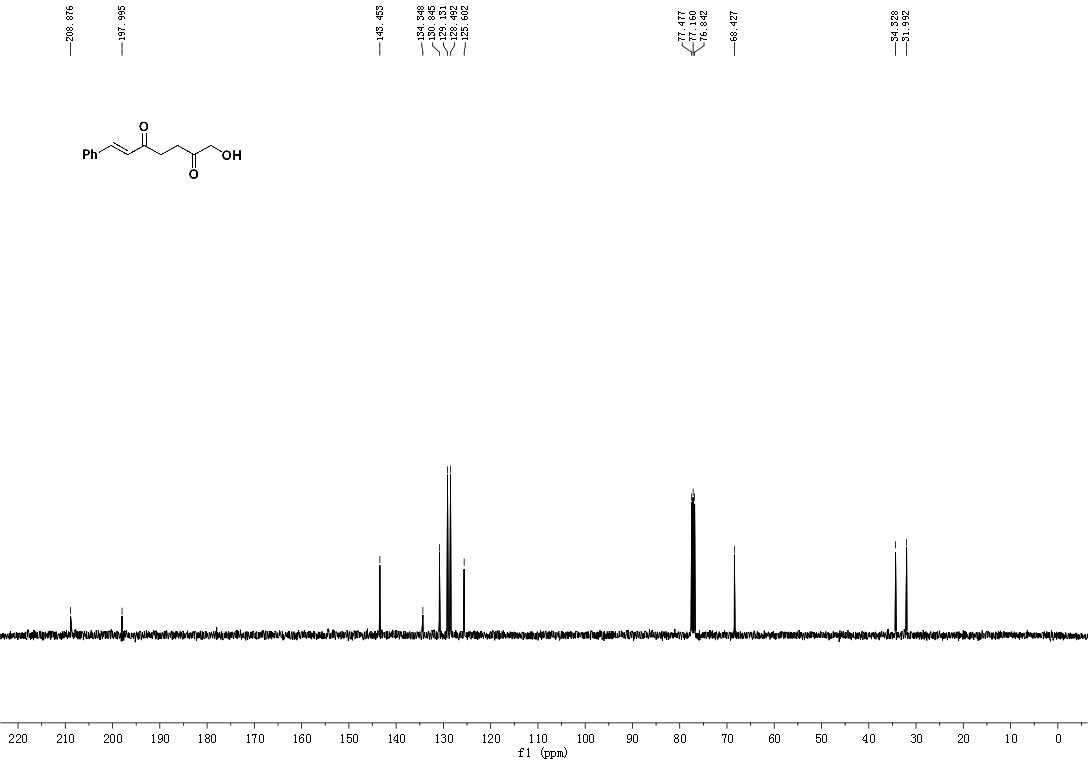


^13^C NMR spectra for **19** (100 MHz, CDCl_3_)


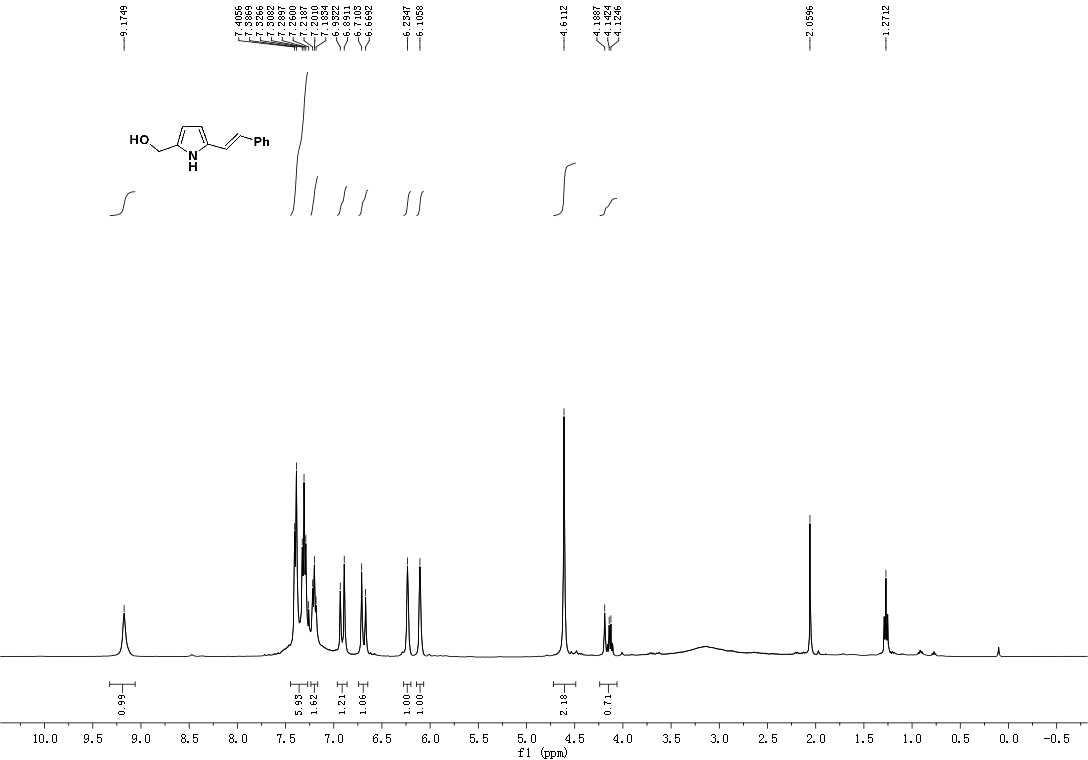


^1^H NMR spectra for crude **20** (400 MHz, CDCl_3_)


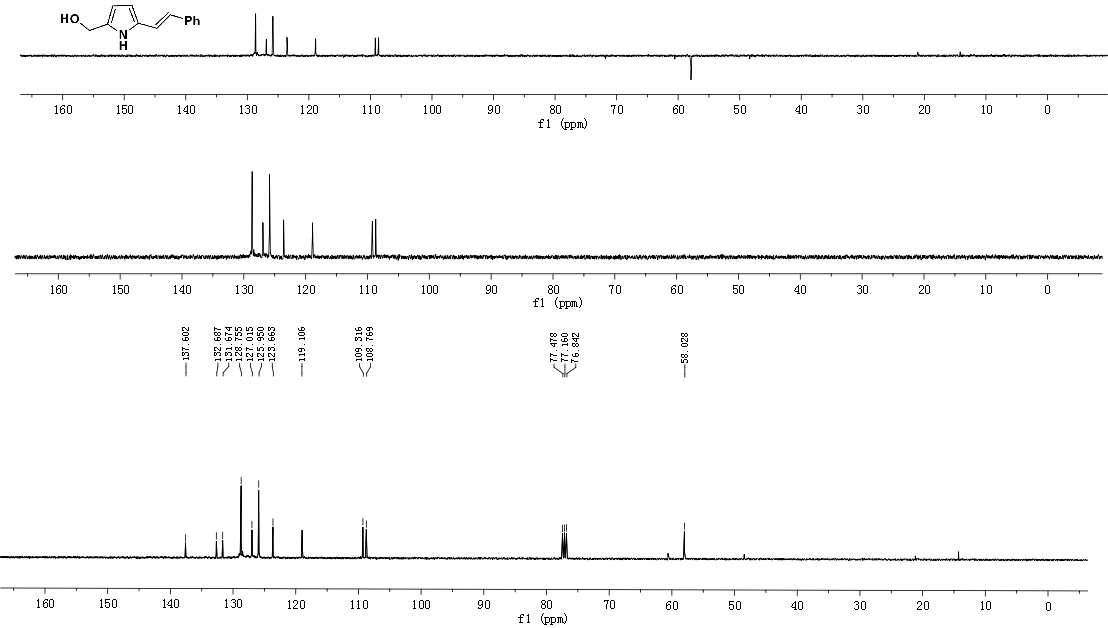


^13^C and DEPT NMR spectra for crude **20** (100 MHz, CDCl_3_)


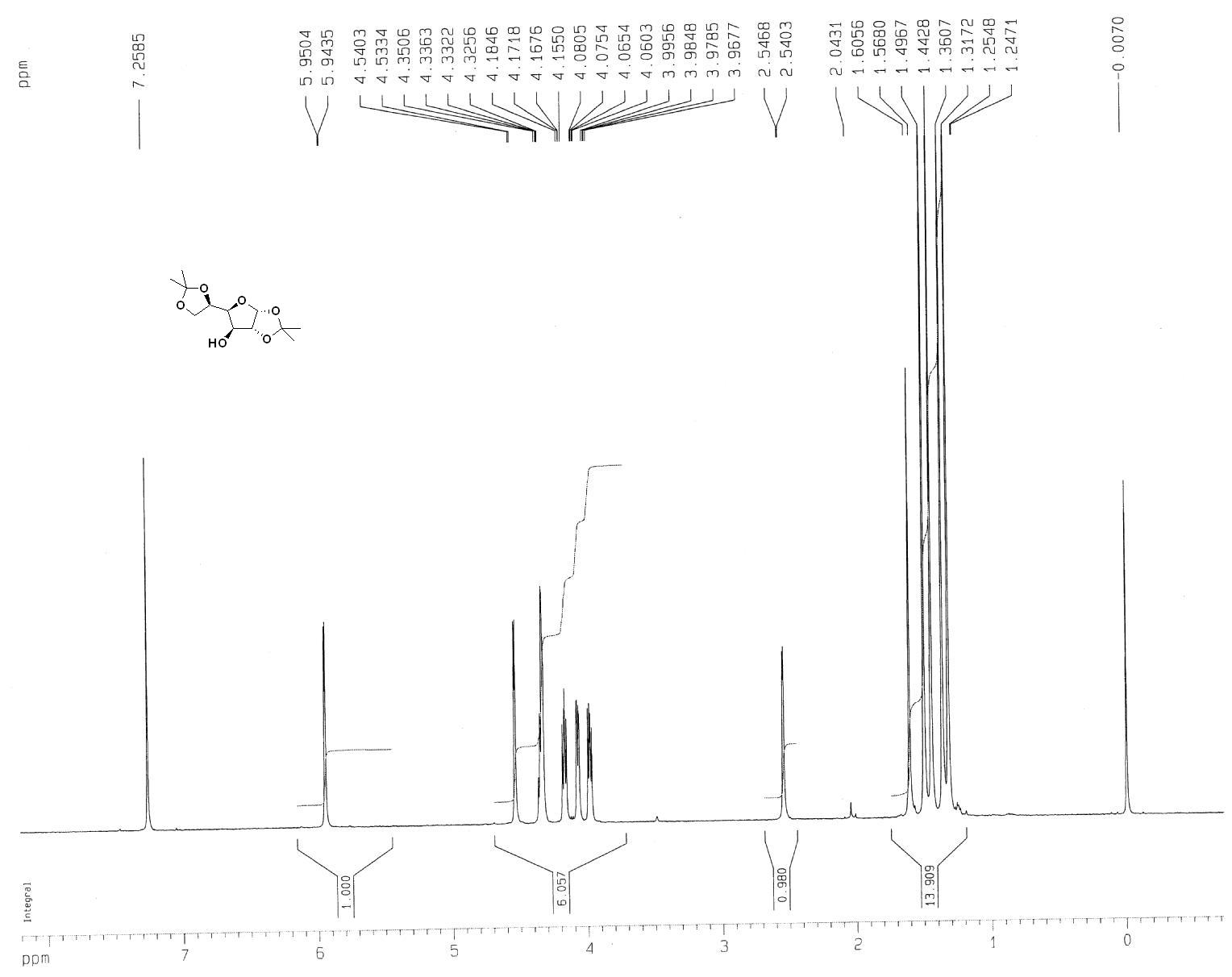


^1^H NMR spectra for **S10** (500 MHz, CDCl_3_)


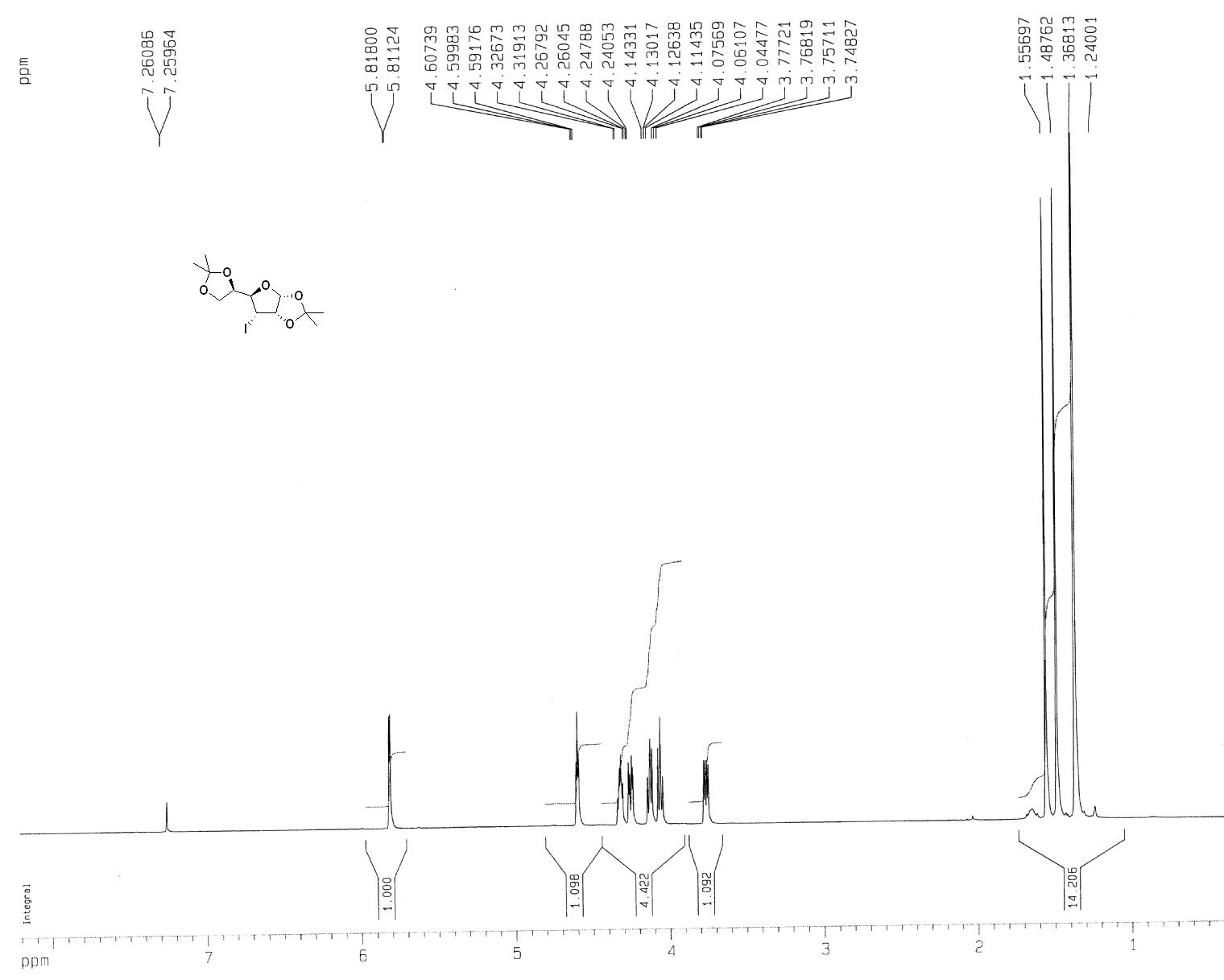


^1^H NMR spectra for **S9** (500 MHz, CDCl_3_)


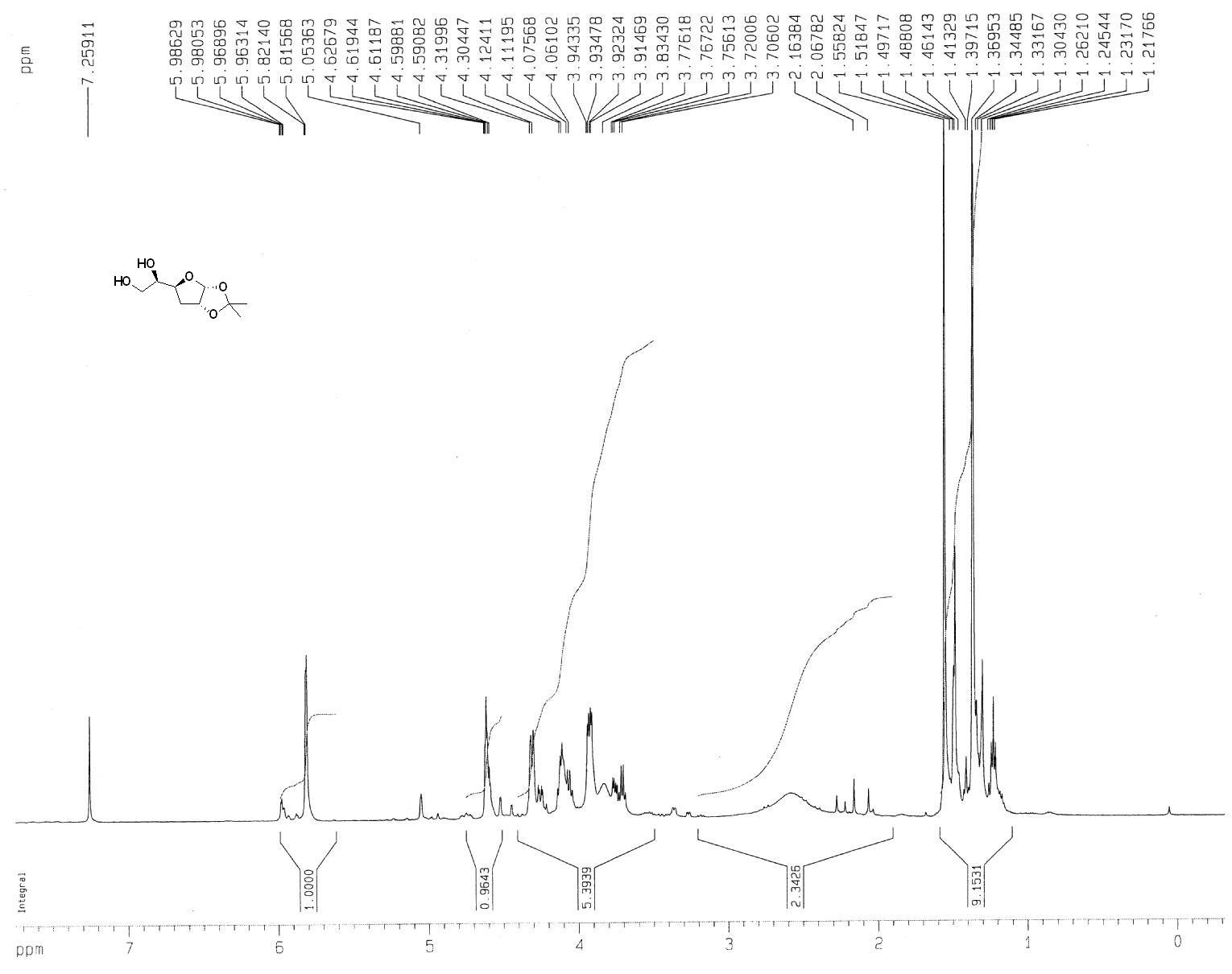


^1^H NMR spectra for crude **S13** (500 MHz, CDCl_3_)


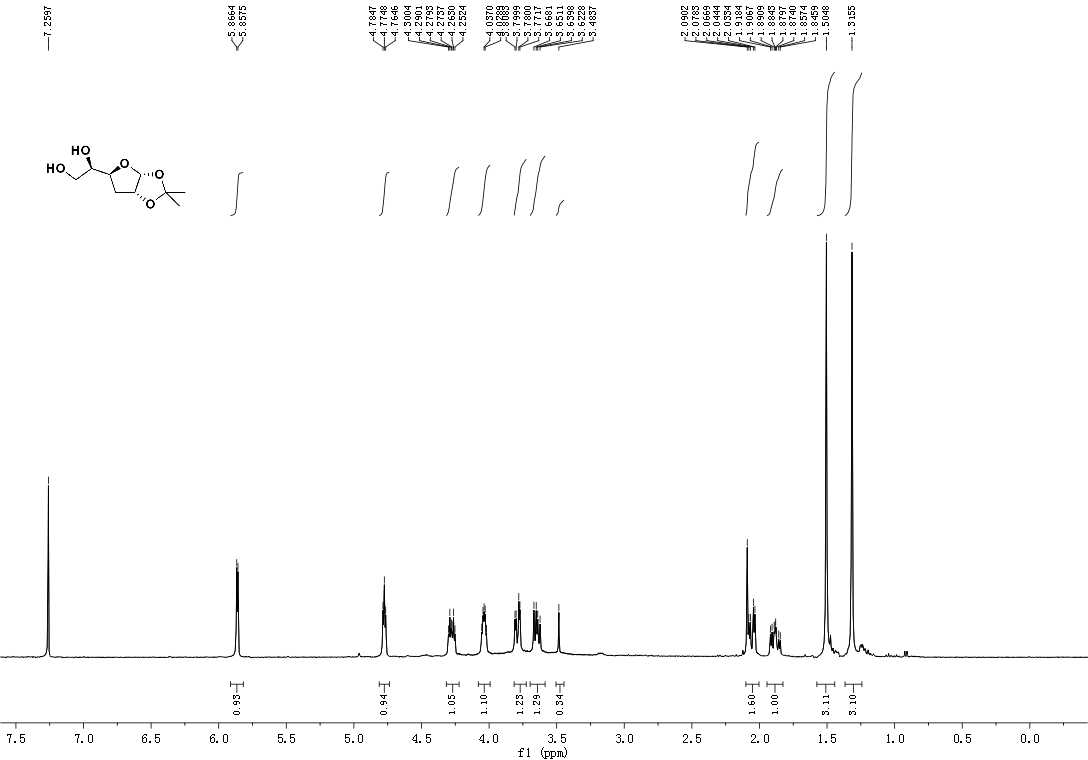


^1^H NMR spectra for **S13** (400 MHz, CDCl_3_)


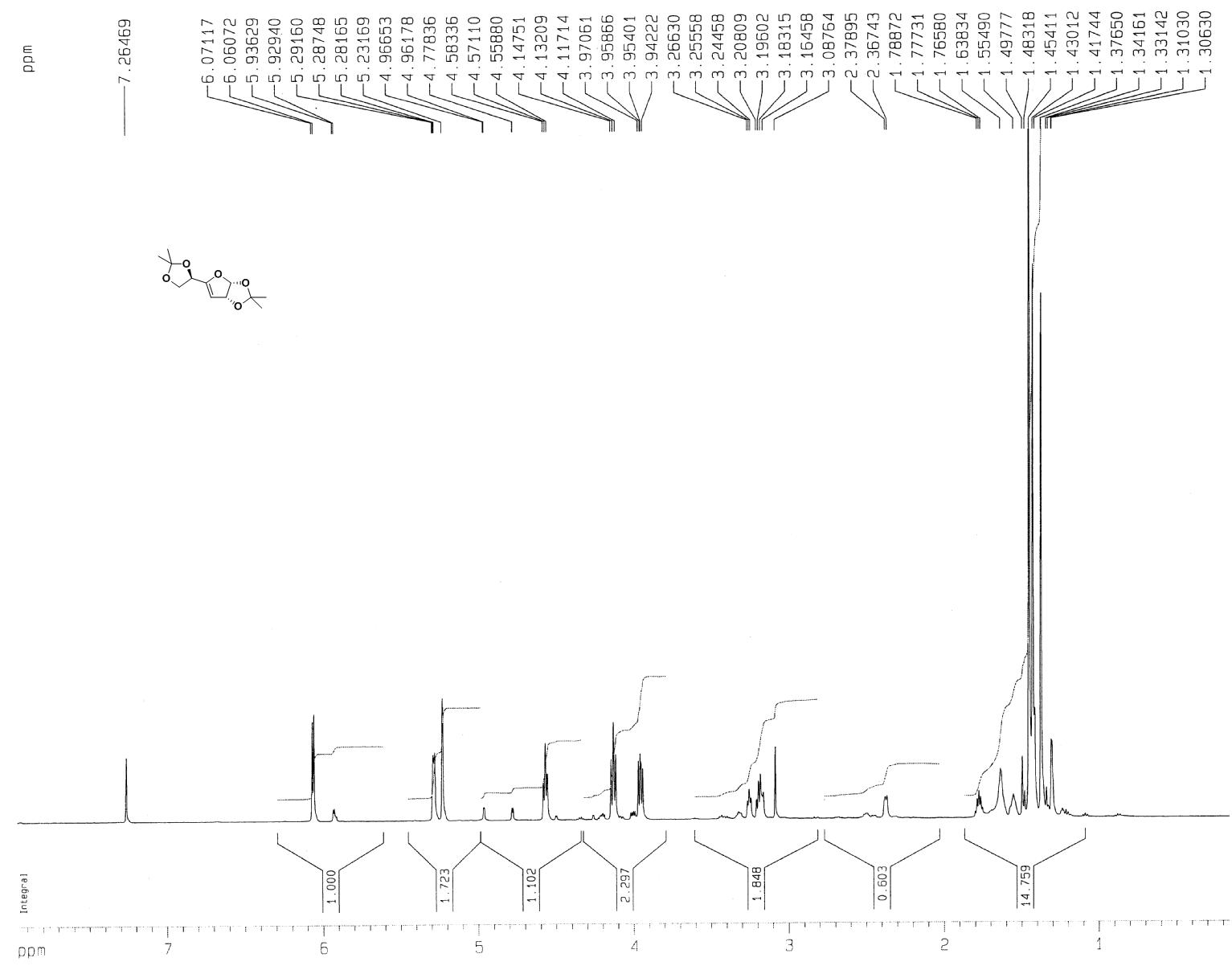


^1^H NMR spectra for crude **S14** (400 MHz, CDCl_3_)


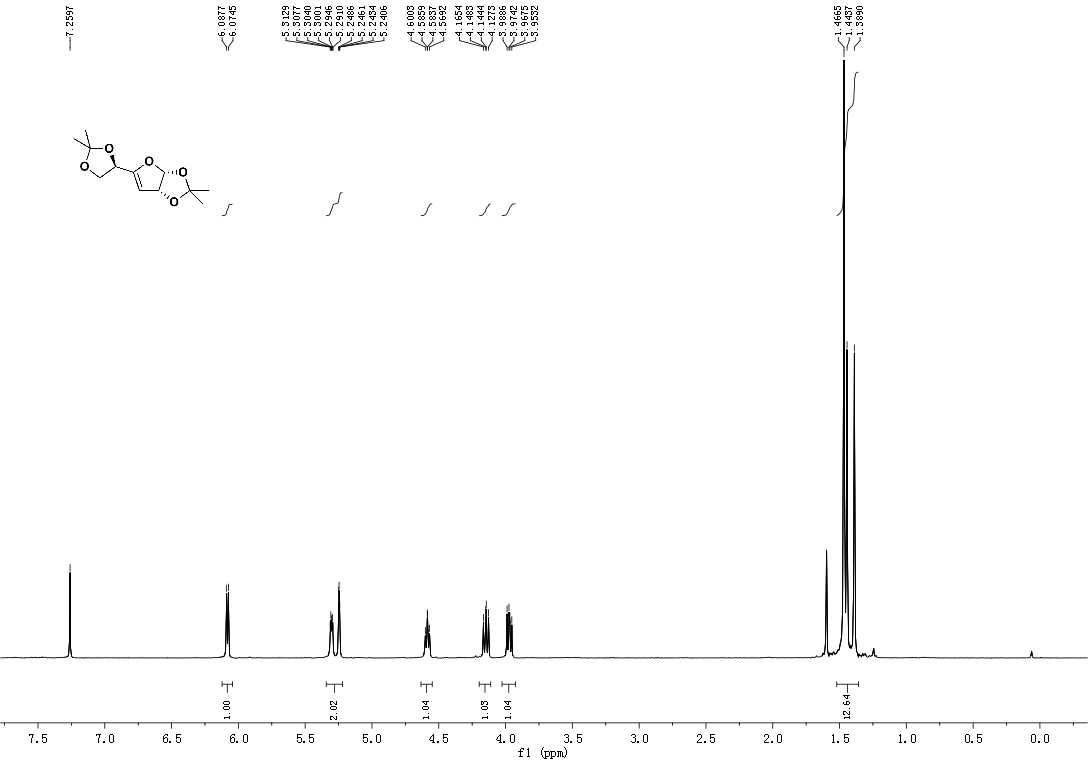


^1^H NMR spectra for **S14** (400 MHz, CDCl_3_)


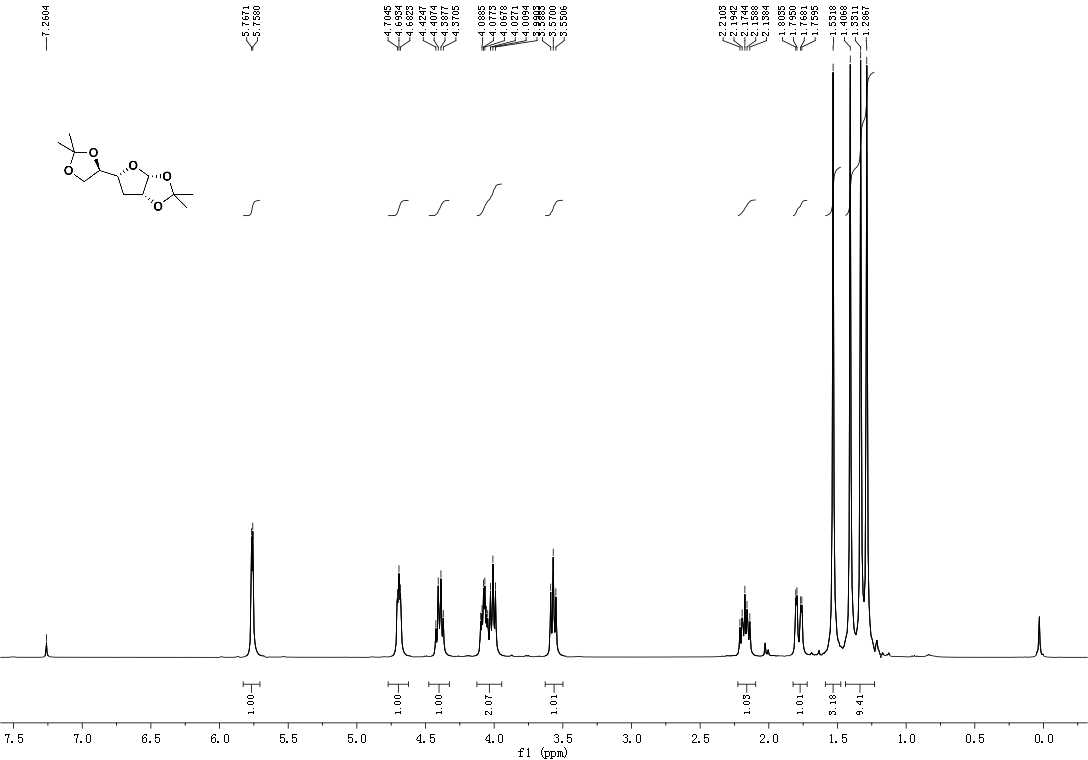


^1^H NMR spectra for **S15** (400 MHz, CDCl_3_)


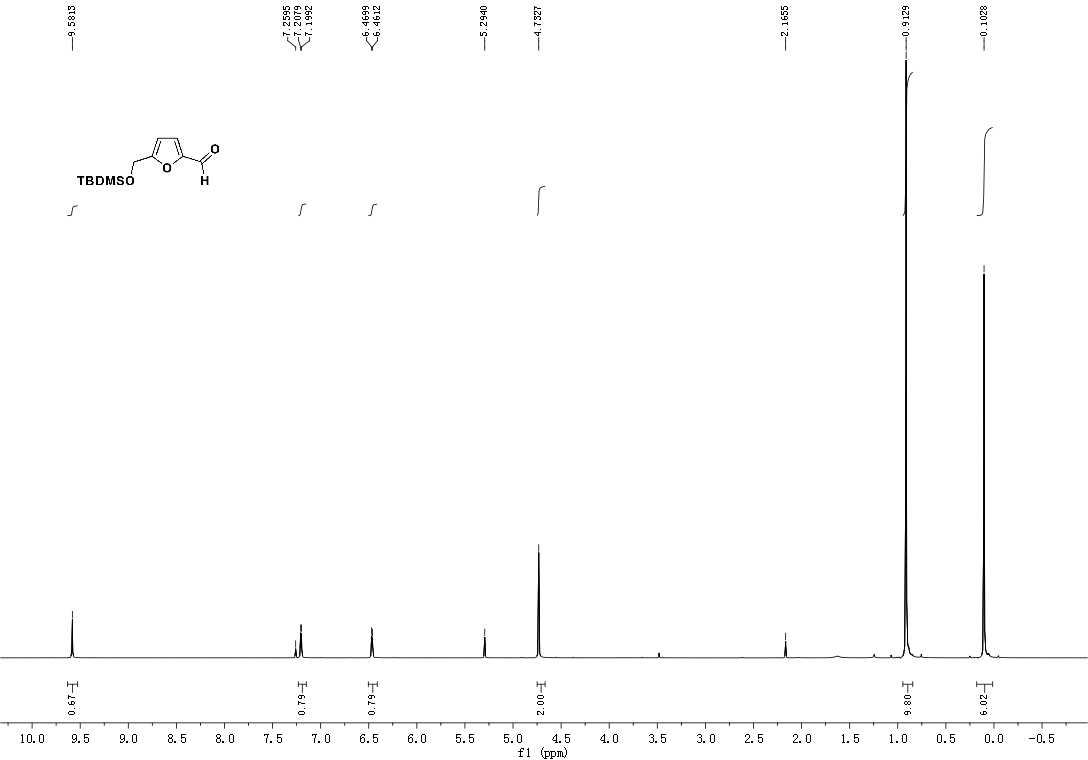


^1^H NMR spectra for **S18** (400 MHz, CDCl_3_)


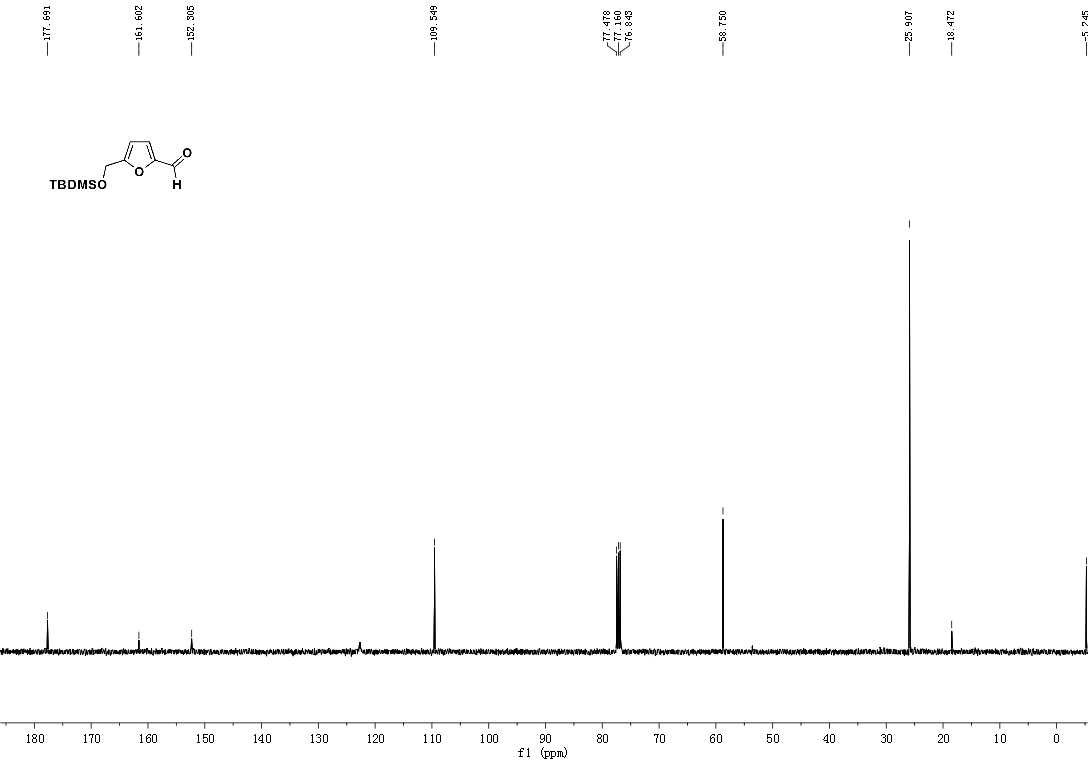


^13^C NMR spectra for **S18** (100 MHz, CDCl_3_)


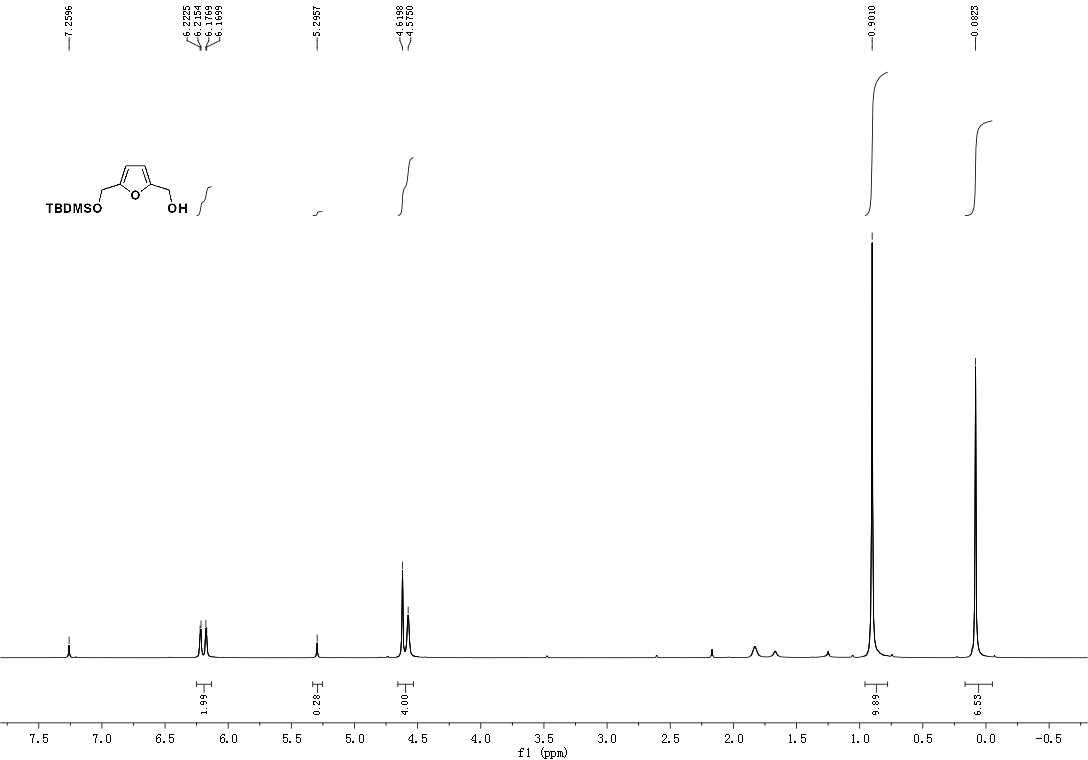


^1^H NMR spectra for **S20** (400 MHz, CDCl_3_)


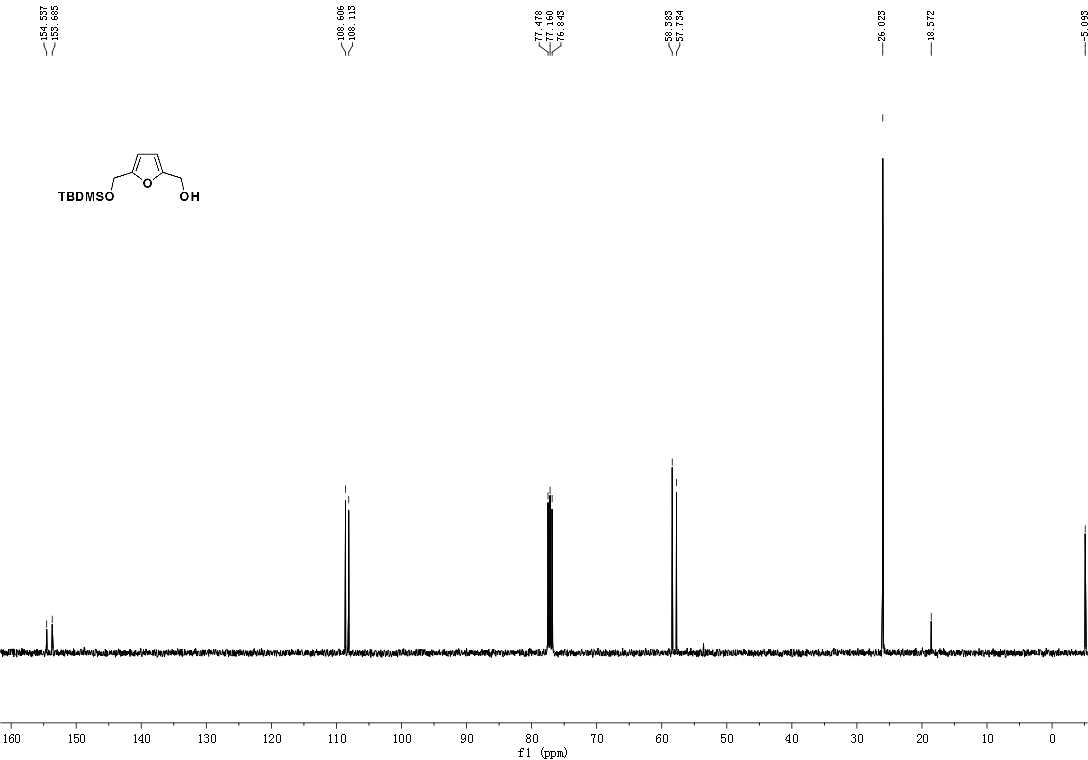


^13^C NMR spectra for **S20** (100 MHz, CDCl_3_)


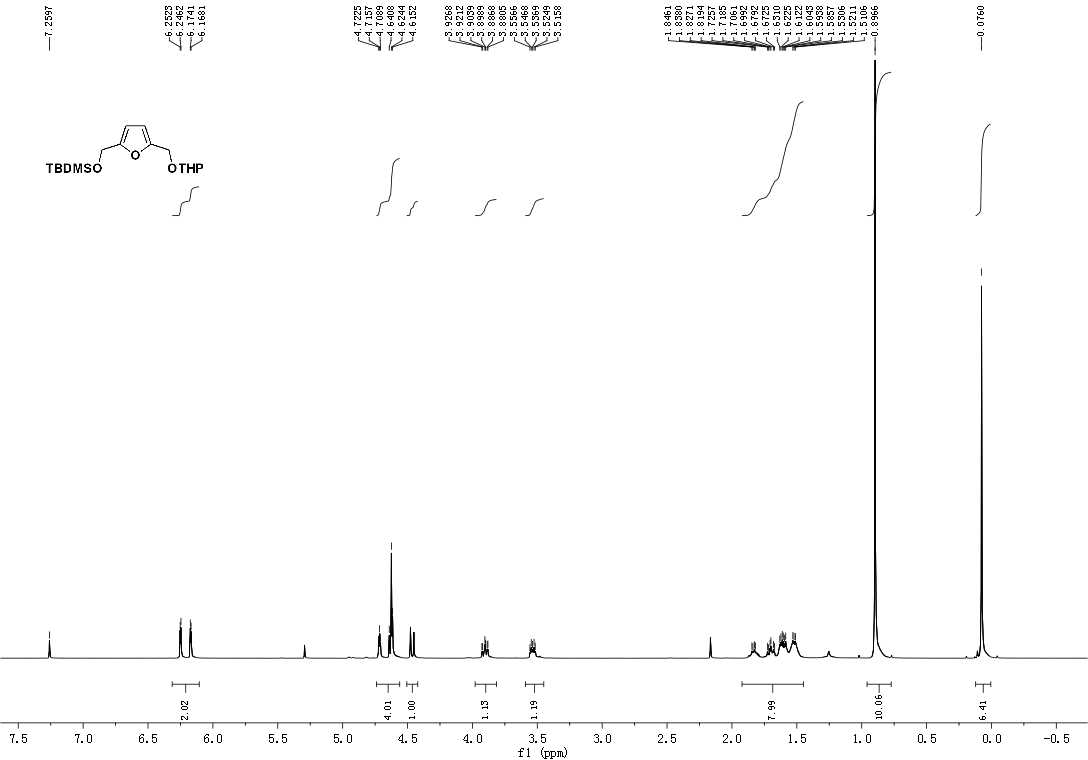


^1^H NMR spectra for **S21** (500 MHz, CDCl_3_)


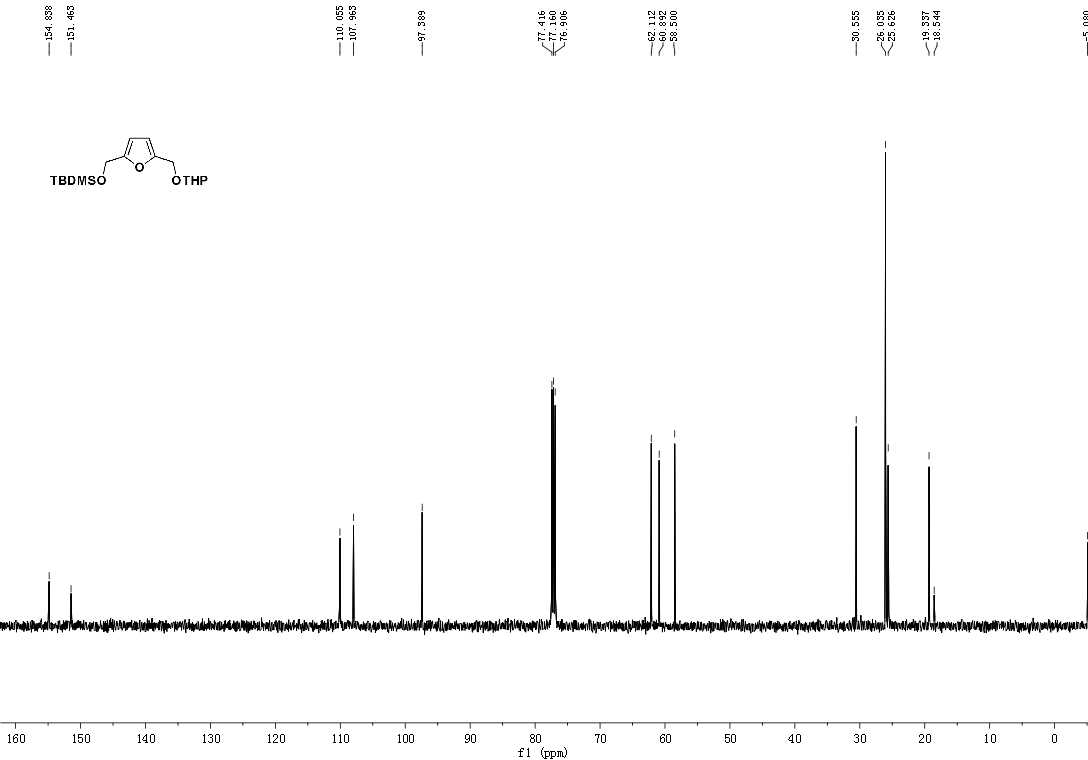


^13^C NMR spectra for **S21** (125 MHz, CDCl_3_)


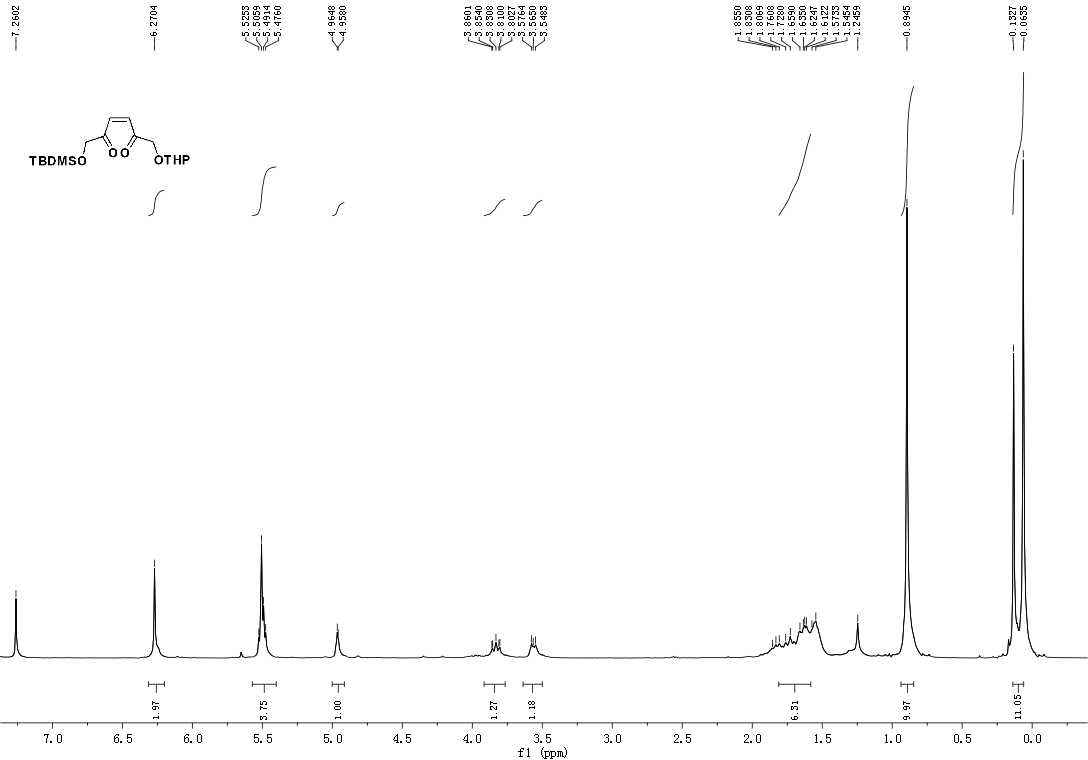


^1^H NMR spectra for **S22** (400 MHz, CDCl_3_)


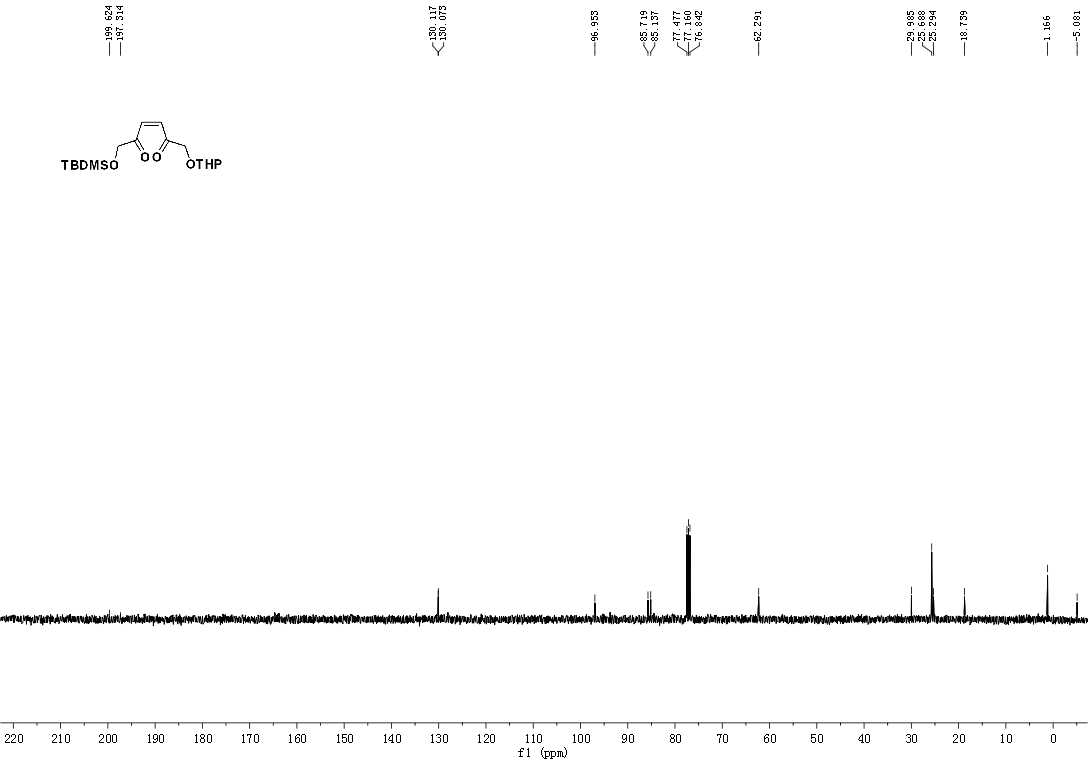


^13^C NMR spectra for **S22** (100 MHz, CDCl_3_)


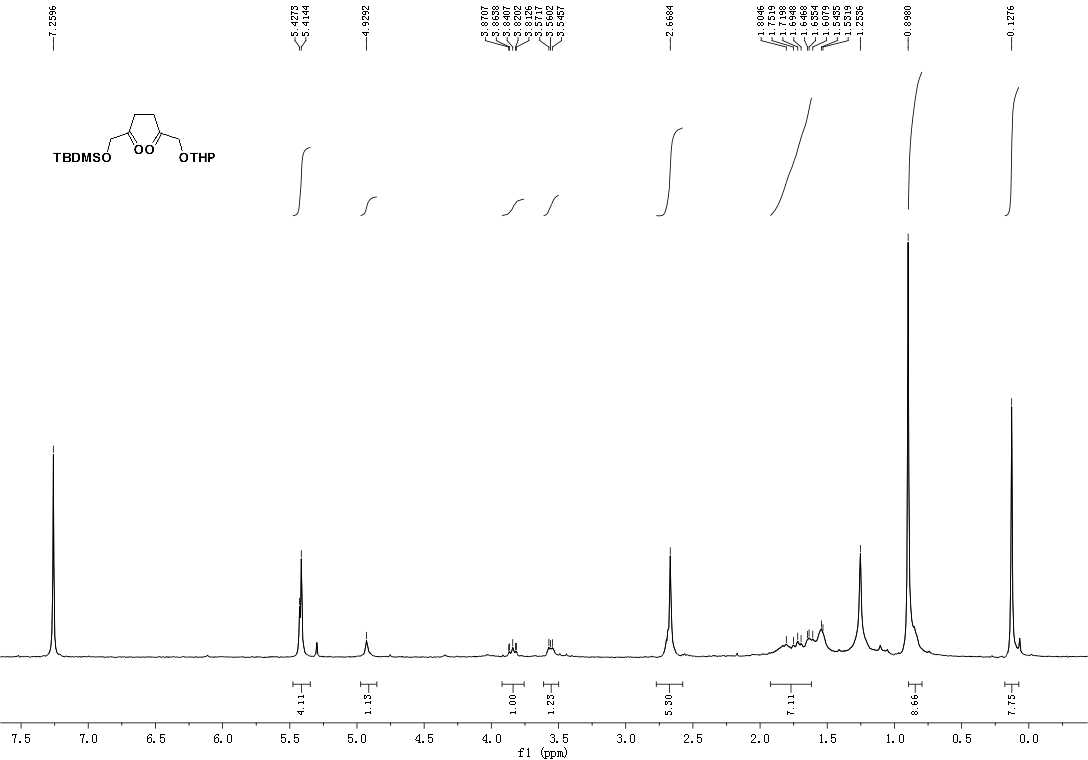


^1^H NMR spectra for **S23** (400 MHz, CDCl_3_)


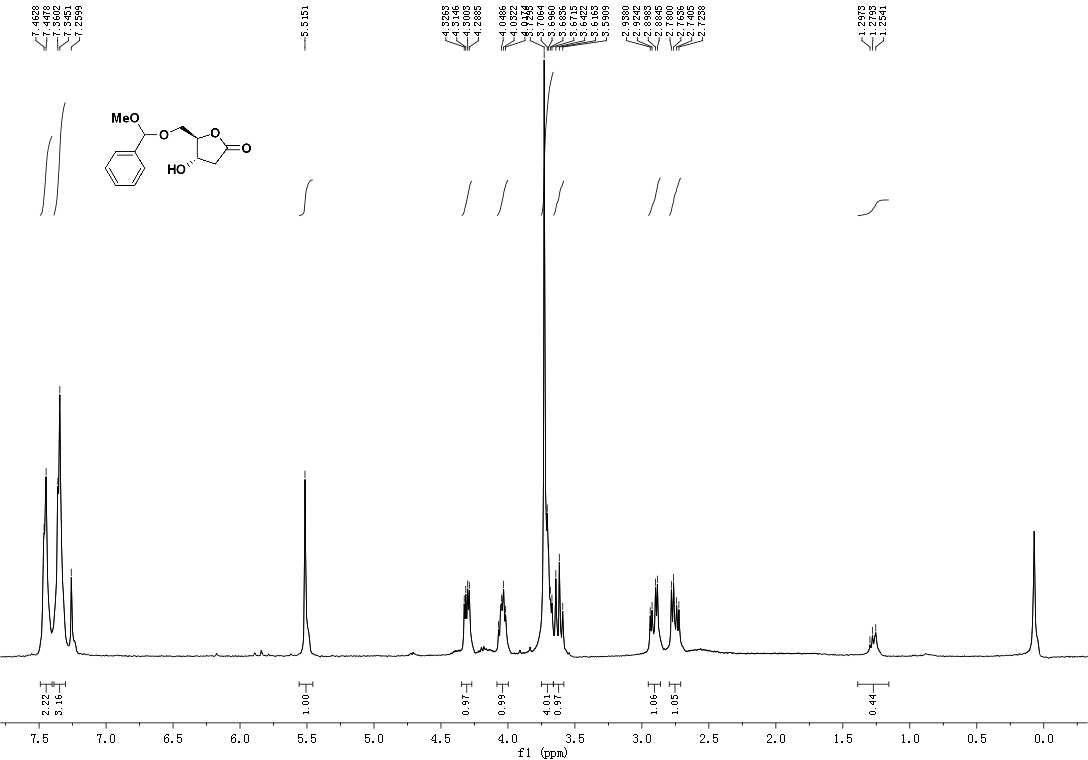


^1^H NMR spectra for **S27** (400 MHz, CDCl_3_)


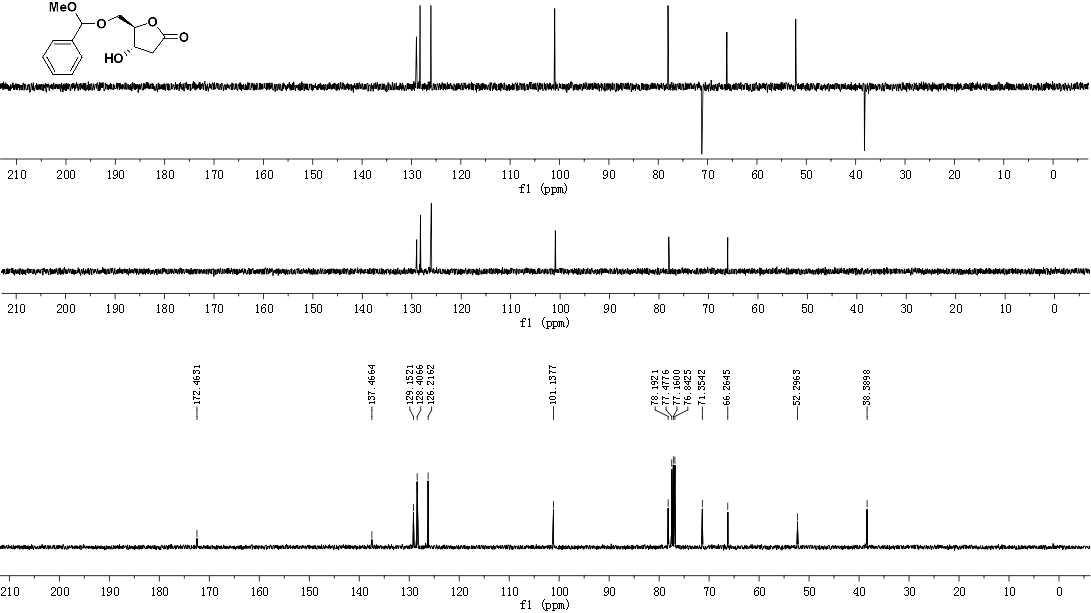


^13^C and DEPT NMR spectra for **S27** (100 MHz, CDCl_3_)


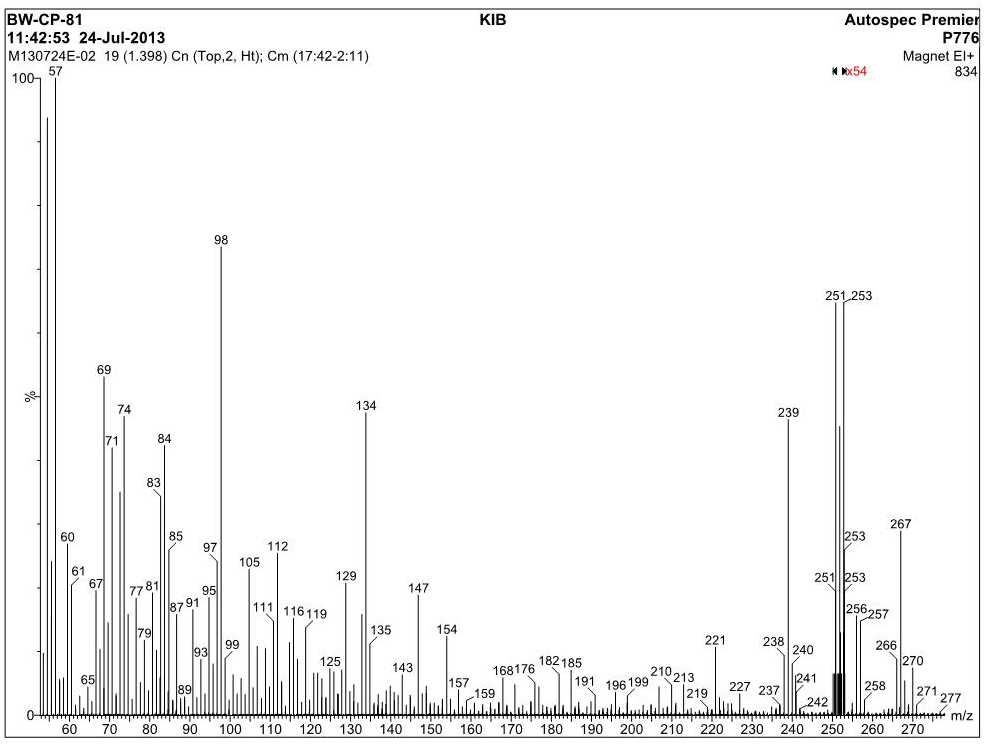


EI-MS spectra for **S27**


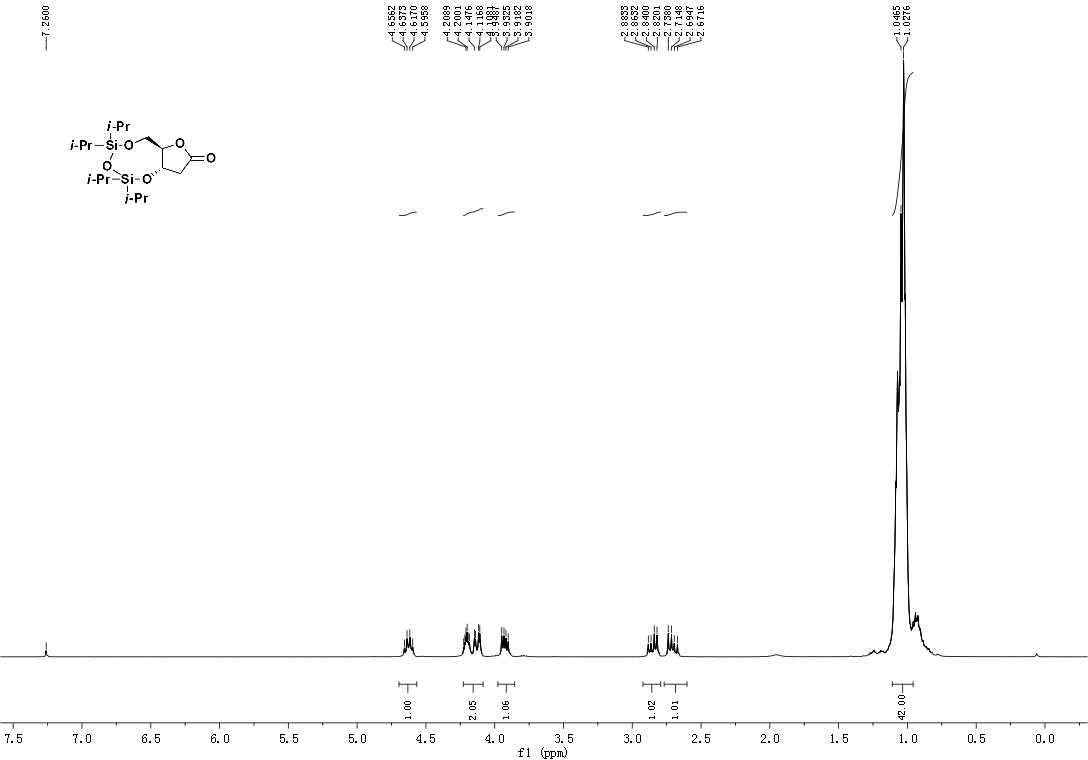


^1^H NMR spectra for **S28** (400 MHz, CDCl_3_)


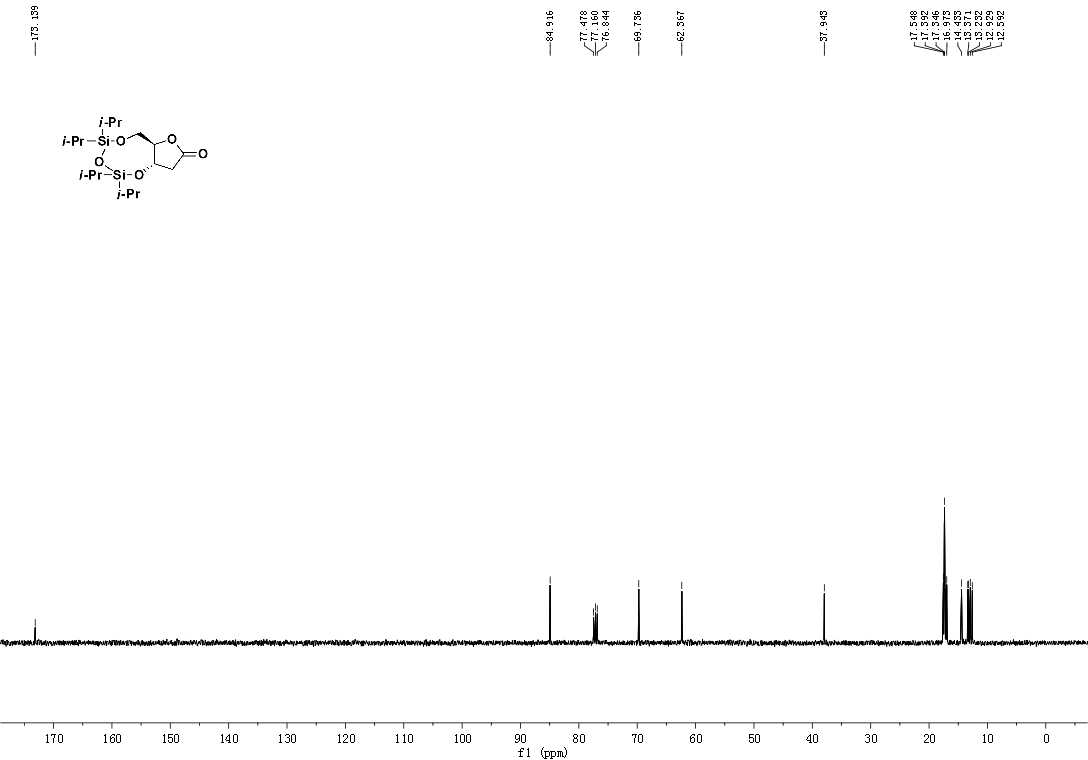


^13^C NMR spectra for **S28** (100 MHz, CDCl_3_)


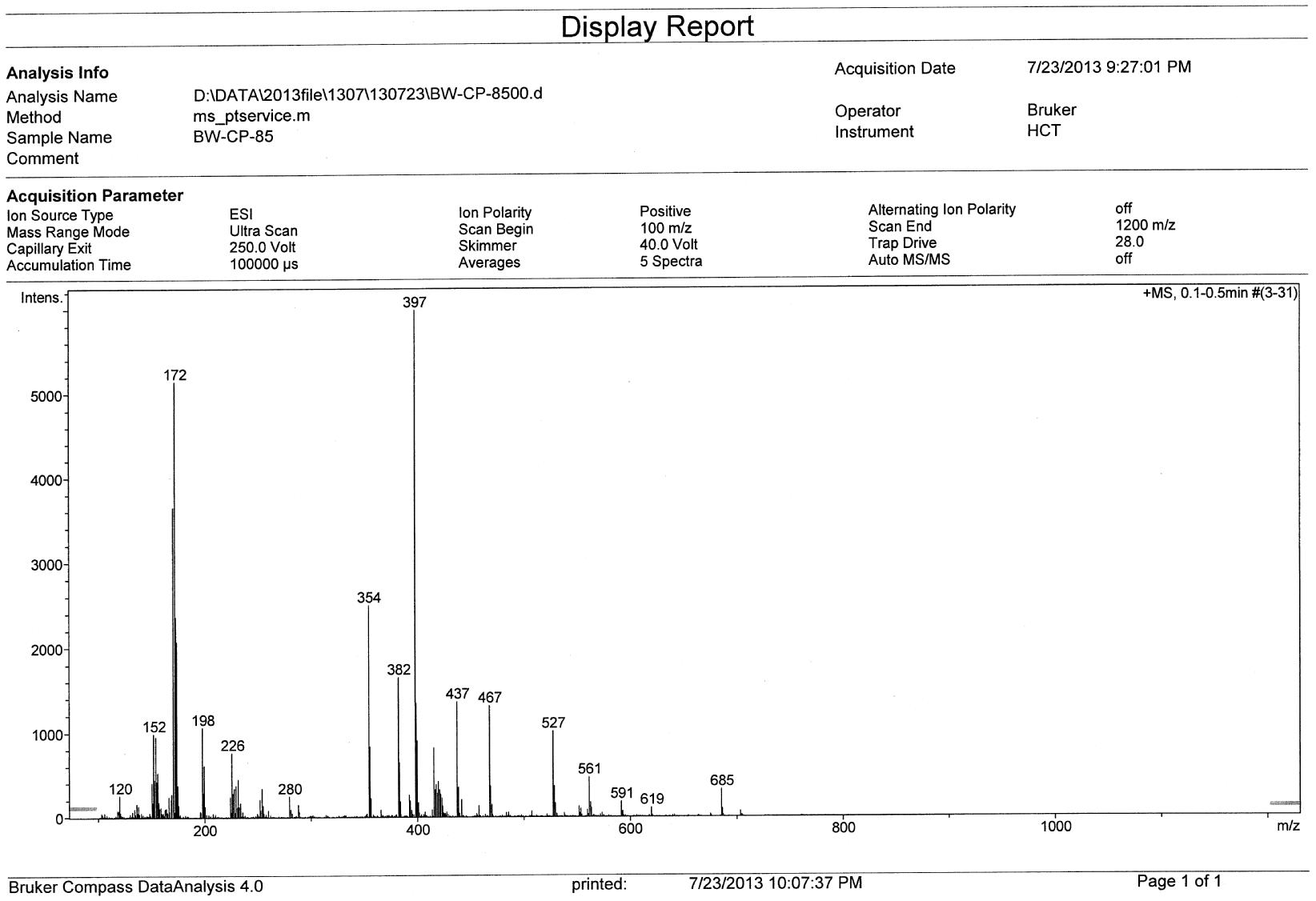


ESI-MS spectra for **S28**


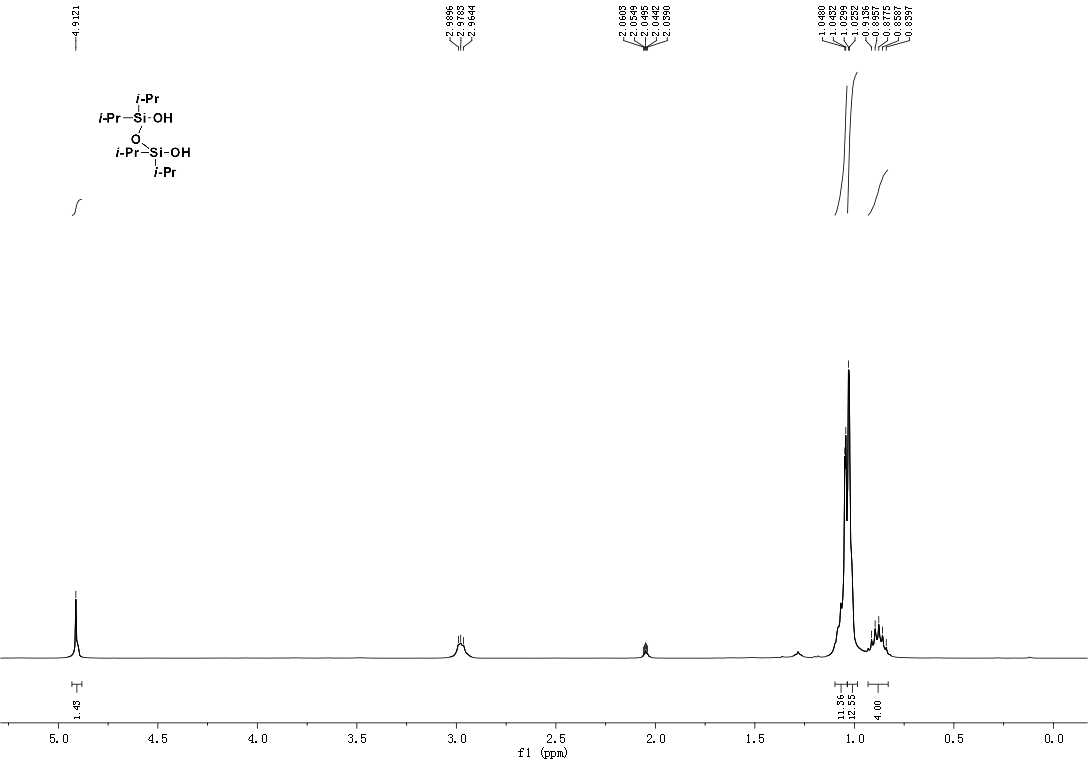


^1^H NMR spectra for diol cleaved fully from **S28** (400 MHz, CD_3_COCD_3_)


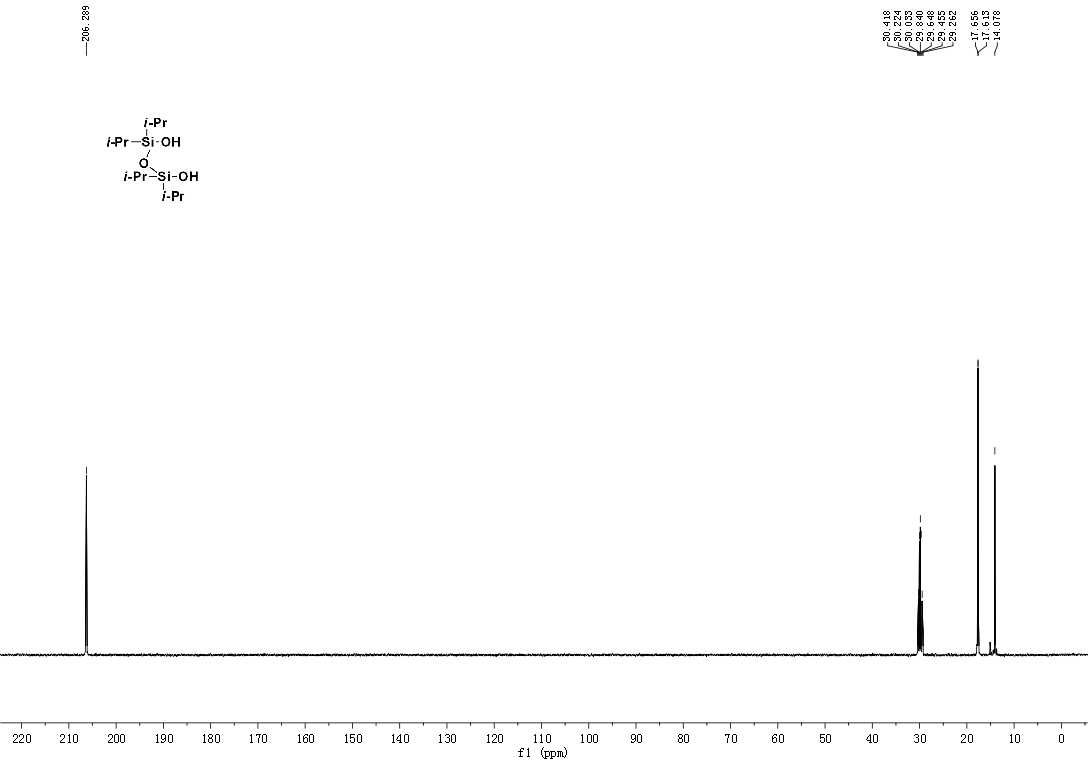


^13^C NMR spectra for diol cleaved fully from **S28** (100 MHz, CD_3_COCD_3_)


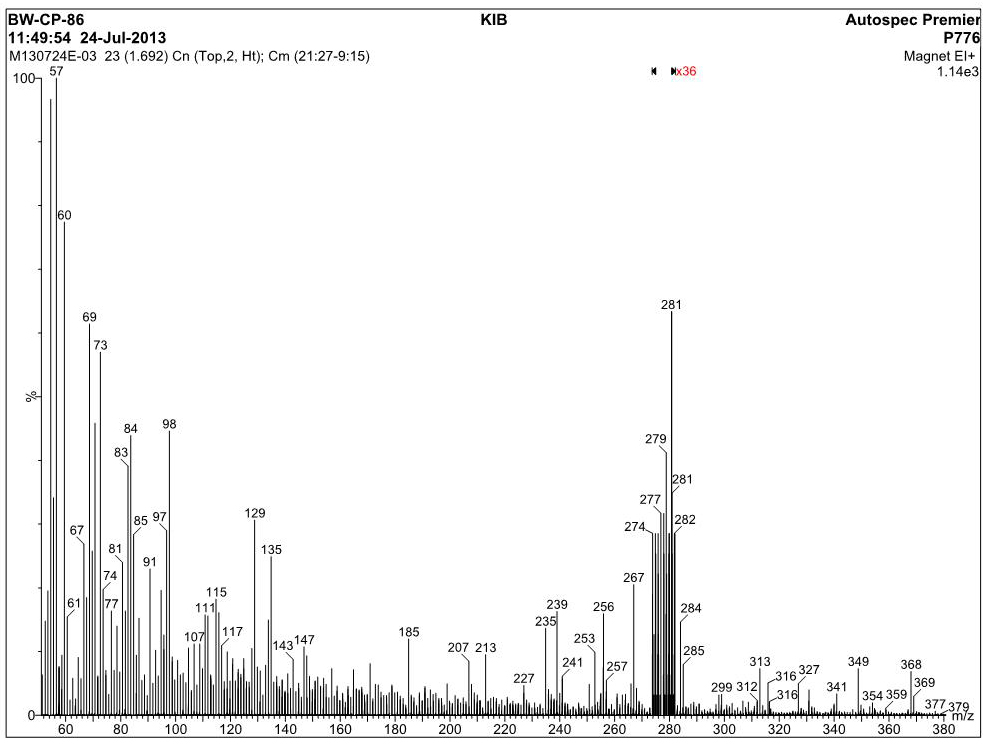


EI-MS spectra for diol cleaved fully from **S28**


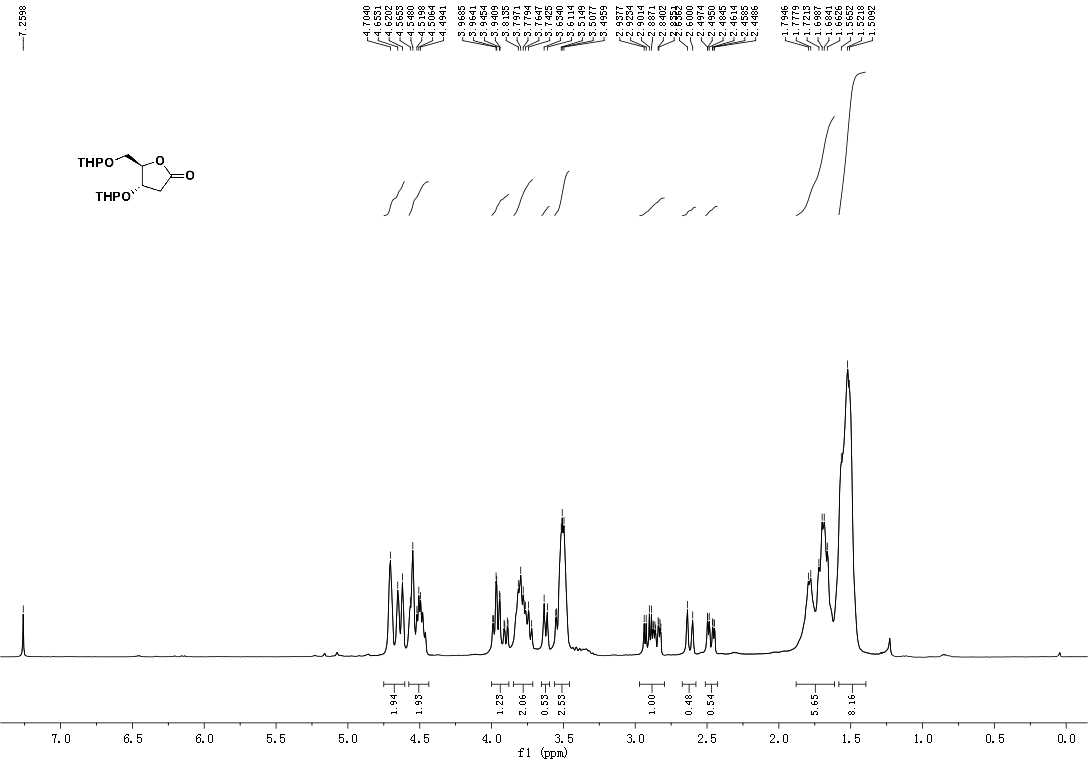


^1^H NMR spectra for **S29** (500 MHz, CDCl_3_)


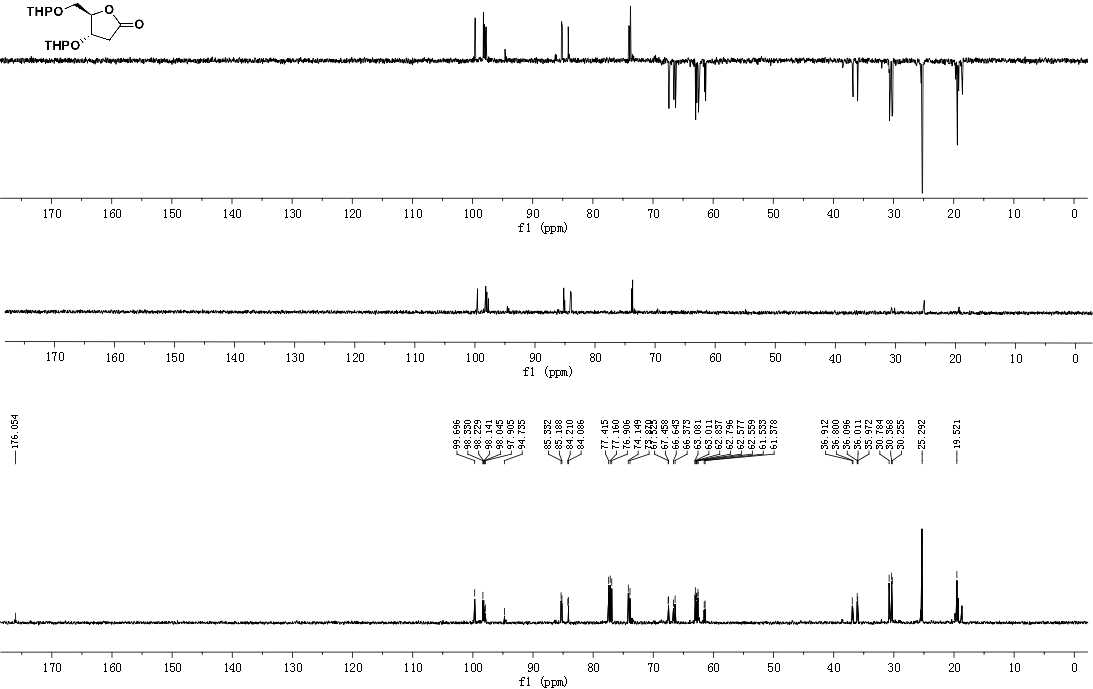


^13^C and DEPT NMR spectra for **S29** (125 MHz, CDCl_3_)


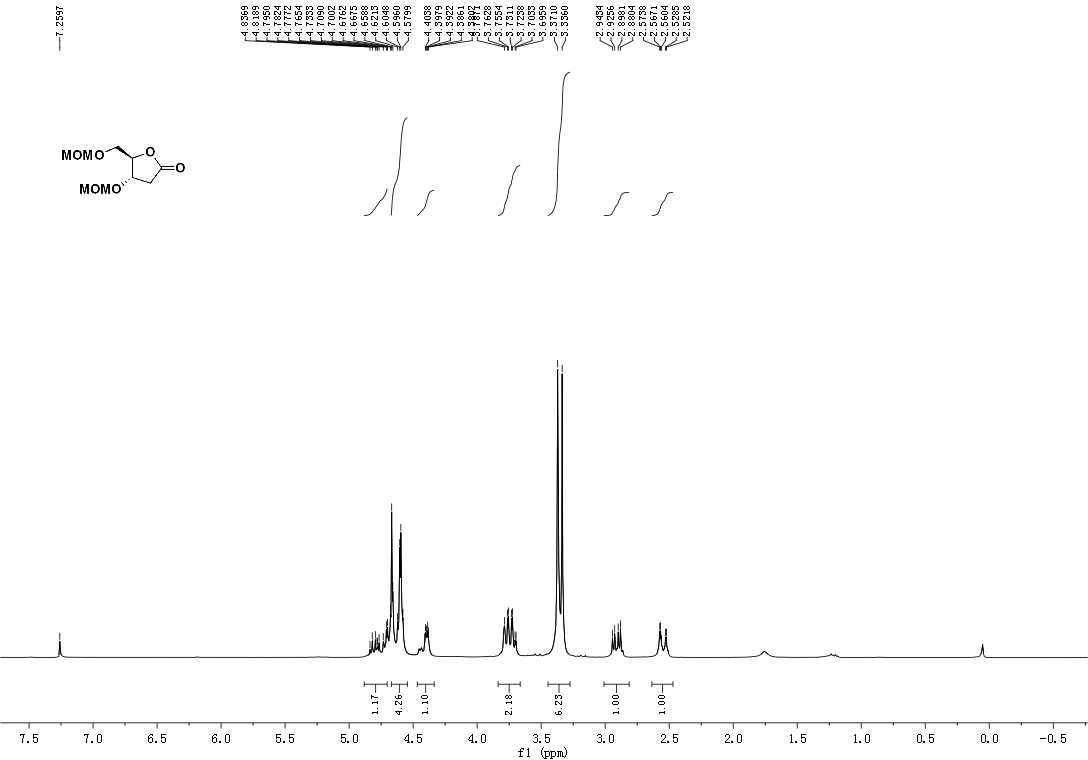


^1^H NMR spectra for **S30** (400 MHz, CDCl_3_)


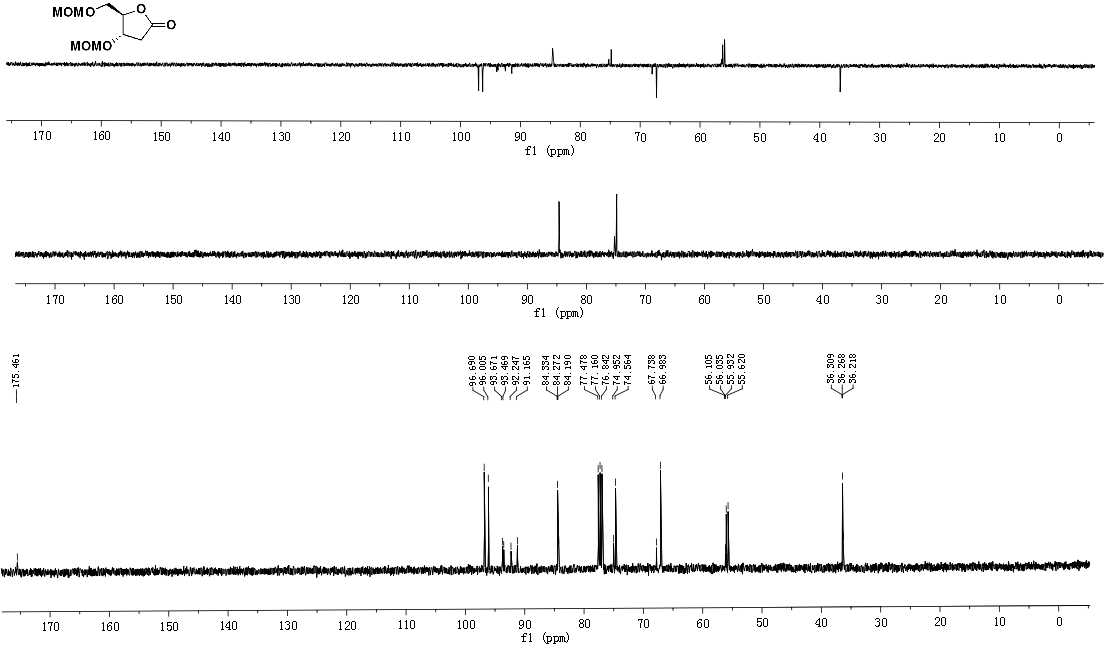


^13^C and DEPT NMR spectra for **S30** (100 MHz, CDCl_3_)


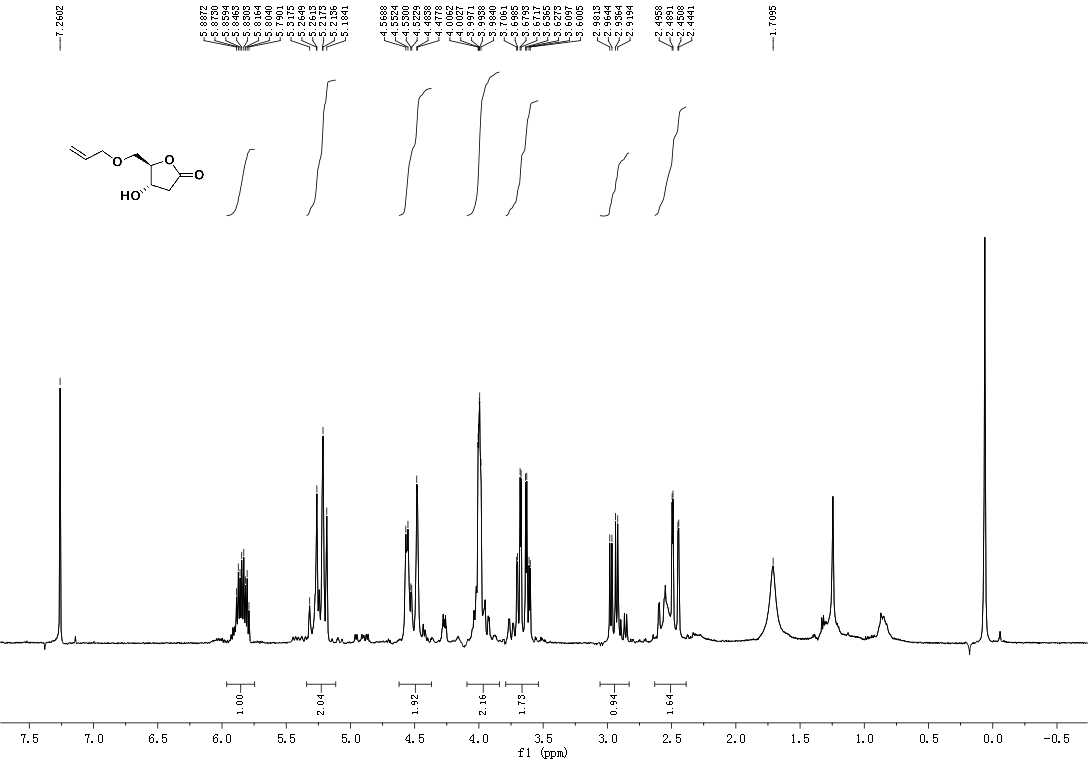


^1^H NMR spectra for **S31** (400 MHz, CDCl_3_)


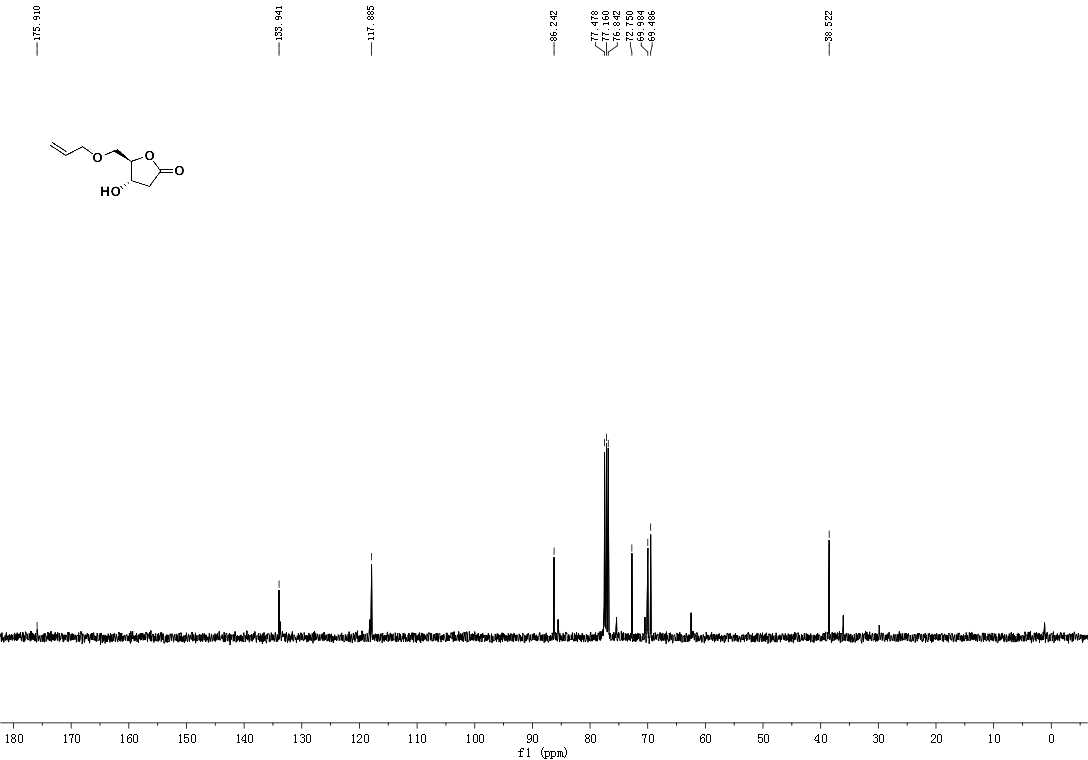


^13^C NMR spectra for **S31** (100 MHz, CDCl_3_)
